# Salt‑responsive transcriptome analysis of canola roots reveals candidate genes involved in the key metabolic pathway in response to salt stress

**DOI:** 10.1038/s41598-022-05700-2

**Published:** 2022-01-31

**Authors:** Weichao Wang, Jiayin Pang, Fenghua Zhang, Lupeng Sun, Lei Yang, Tingdong Fu, Liang Guo, Kadambot H. M. Siddique

**Affiliations:** 1 The Key Laboratory of Oasis Eco-Agriculture, Xinjiang Production and Construction Crops, Shihezi University, Xinjiang, 832003 China; 2 The UWA Institute of Agriculture and School of Agriculture and Environment, The University of Western Australia, Perth, WA 6001 Australia; 3 National Key Laboratory of Crop Genetic Improvement, Huazhong Agricultural University, Wuhan, China

**Keywords:** Plant sciences, Plant hormones, Plant molecular biology, Plant signalling

## Abstract

Salinity is a major constraint on crop growth and productivity, limiting sustainable agriculture in arid regions. Understanding the molecular mechanisms of salt-stress adaptation in canola is important to improve salt tolerance and promote its cultivation in saline lands. In this study, roots of control (no salt) and 200 mM NaCl-stressed canola seedlings were collected for RNA-Seq analysis and qRT-PCR validation. A total of 5385, 4268, and 7105 DEGs at the three time points of salt treatment compared to the control were identified, respectively. Several DEGs enriched in plant signal transduction pathways were highly expressed under salt stress, and these genes play an important role in signaling and scavenging of ROS in response to salt stress. Transcript expression in canola roots differed at different stages of salt stress, with the early-stages (2 h) of salt stress mainly related to oxidative stress response and sugar metabolism, while the late-stages (72 h) of salt stress mainly related to transmembrane movement, amino acid metabolism, glycerol metabolism and structural components of the cell wall. Several families of TFs that may be associated with salt tolerance were identified, including ERF, MYB, NAC, WRKY, and bHLH. These results provide a basis for further studies on the regulatory mechanisms of salt stress adaptation in canola.

## Introduction

Soil salinization is a major constraint on crop growth and productivity, severely affecting the sustainability of agriculture, especially in arid and semi-arid regions^1^. Globally, it is estimated that a total of approximately 1 billion hectares of land are affected by salinity, with approximately 6% of the world's terrestrial land being affected by primary salinization. In addition, about 20% of all cropland and between 1/4 and 1/3 of irrigated land are affected by secondary salinization^2,3^. In China, the total area of saline land is about 8.11 × 10^7^ ha, approximately 9% of the total land area^4^. Xinjiang had the largest and most widely distributed area of salinization in China^5,6^, with 1.263 × 10^6^ ha of arable land affected by salinization, accounting for 31.10% of the total arable land area in the region^7^. Land salinization has become an important factor limiting the sustainable development of agriculture in Xinjiang.

High salinity conditions produce a range of osmotic stress, oxidative damage, and ion toxicity in plants that affect plant growth and development^8^. Plants can reduce salt stress damage through physiological, biochemical, and molecular regulatory mechanisms, including signaling and induction of antioxidant enzyme activity, regulation of osmotic pressure and ion homeostasis, and scavenging of reactive oxygen species^9^. Salt tolerance in plants is a complex trait influenced by genetic factors. To uncover the molecular mechanisms of salt tolerance in plants, the screening of salt tolerance genes in salt tolerant species has been intensively investigated. In recent years, studies have shown that the transcription and expression of salt-responsive genes play important roles in transcriptional regulation of signal transduction, the activity of antioxidant enzyme systems, and the accumulation of osmoregulatory substances in plants^10–12^. Therefore the screening of salt-tolerant genes is important for a comprehensive understanding of the molecular mechanisms underlying salt tolerance in plants. Numerous studies have shown that gene regulation under abiotic stress is influenced by multiple transcriptional cascades^13–16^. Phytohormones such as abscisic acid (ABA) and gibberellin (GA), acting as endogenous signaling molecules, are important regulators of salt stress responses^17^. Salt oversensitive (SOS) signaling genes in plants are involved in salt adaptation and the maintenance of intracellular homeostasis^18^. Yan et al.^10^ showed that WRKY responds to salt stress through ABA signaling and regulation of ROS production in plant cells. Overexpression of MYB genes enhanced ROS scavenging and inhibited cell membrane damage in tomato, thereby improving salt tolerance^19^. Song et al.^20^ showed that MYB affected salt tolerance in wheat by regulating ion homeostasis to maintain osmotic balance and reduce ROS levels, while Zhang et al.^21^ also showed that the MYB transcription factor AtMYB49 regulated salt tolerance in *Arabidopsis* by regulating cuticle formation and antioxidant defense.

China is one of the four major producing regions in the world for canola cultivation. In 2017, the planted area reached 6.65 × 10^6^ ha, accounting for about 20% of the global canola planting area^6,22^. Canola is not only one of the most important oilseed crops, but also potentially an ideal plant for saline land restoration. Currently, there are limited studies on the molecular mechanisms of canola at the transcriptomics level under salt stress. So far, most studies on canola were undertaken at the leaf level, with limited studies on other tissues, particularly roots^23^. In the present study, we performed transcriptome analysis of canola roots after different duration of NaCl stress (2 h, 24 h, and 72 h) to reveal the transcriptomic responses in response to salt stress. This work brings new insights into the dynamics of major differential functional genes and related transcriptional regulatory pathways in canola under salt stress.

## Results

### Transcriptome analysis

Sequencing libraries were prepared from roots of *Brassica_napus* treated with 200 mM NaCl for 0, 2, 24, and 72 h to study gene expression characteristics. Seedling plants with five leaves were used for mRNA isolation and isolated mRNA was subjected to paired-end sequencing using the Illumina HiSeqTM2500 method. 12 libraries (4 salt stress stages × 3 biological replicates) were sequenced and a total of 664, 382, 242 raw reads were generated, with 91.64%-94.13% Q30 base percentages and 46.03%-48.80% GC content for all samples (Table S1). The raw reads were filtered to remove low quality reads and a total of 332,191,121 clean reads were finally used for comparison (Table S1). The clean reads were then mapped to the *Brassica_napus* reference transcriptome (*Brassica_napus* genome v4.1) with an average mapping rate of 63.14% (45.50%-79.66%).

**Figure 1:**
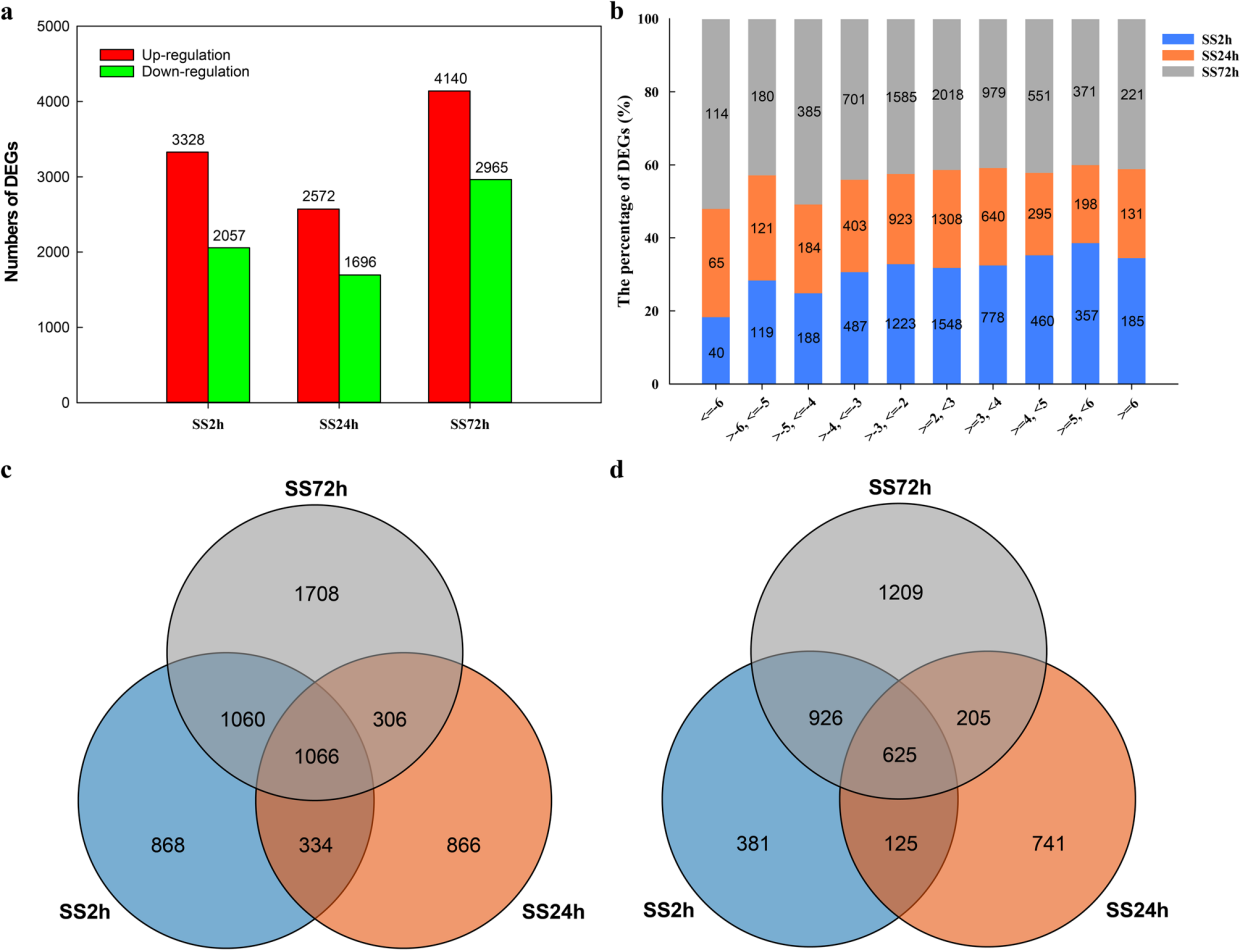
The number and distribution of differentially expressed genes (DEGs) in canola roots. (**a**) The number of DEGs that were up- or down-regulated at the three time points of NaCl stress. (**b**) The distribution of DEGs with different fold changes (Log_2_-transformed). (**c**) Venn diagram showing up-regulated DEGs overlapping at the three time points of NaCl stress or up-regulated DEGs unique to each NaCl stress time points. (**d**) Venn diagram showing down-regulated DEGs overlapping at the three time points of NaCl stress or down-regulated DEGs unique to each NaCl stress time points. SS2h, SS24h and SS72h in the figure indicate the comparison between NaCl stress for 2 h, 24 h, and 72 h and the control, respectively.

Differentially expressed genes (DEGs) for all samples were identified using R package DESeq2 with |log_2_FoldChange|> 2 and padj < 0.05. Compared with the control (0 h), the number of differentially expressed genes was 5385 at 2 h and 4268 at 24 h (reduced compared to 2 h), but as the duration of NaCl exposure increased to 72 h, more DEGs were identified (7105). The number of up- and down-regulated DEGS was 3328, 2572, 4140, and 2057, 1696, 2965 after 2 h, 24 h, and 72 h of NaCl treatment, respectively (Fig. 1a, Figure S1). These data indicate that there were more differential genes at the early stage of salt stress (2 h) and plateaued at 24 h (both up- and down-regulated DEGs decreased compared to 2 h), and DEGs numbers obviously increased at later stages of salt stress (72 h) (both up- and down-regulated DEGs were more than 2 h and 24 h), and more DEGs were up-regulated (3328, 2572, and 4140) than down-regulated (2057, 1696, and 2965, respectively) at all treatment stages of NaCl stress.

**Figure 2:**
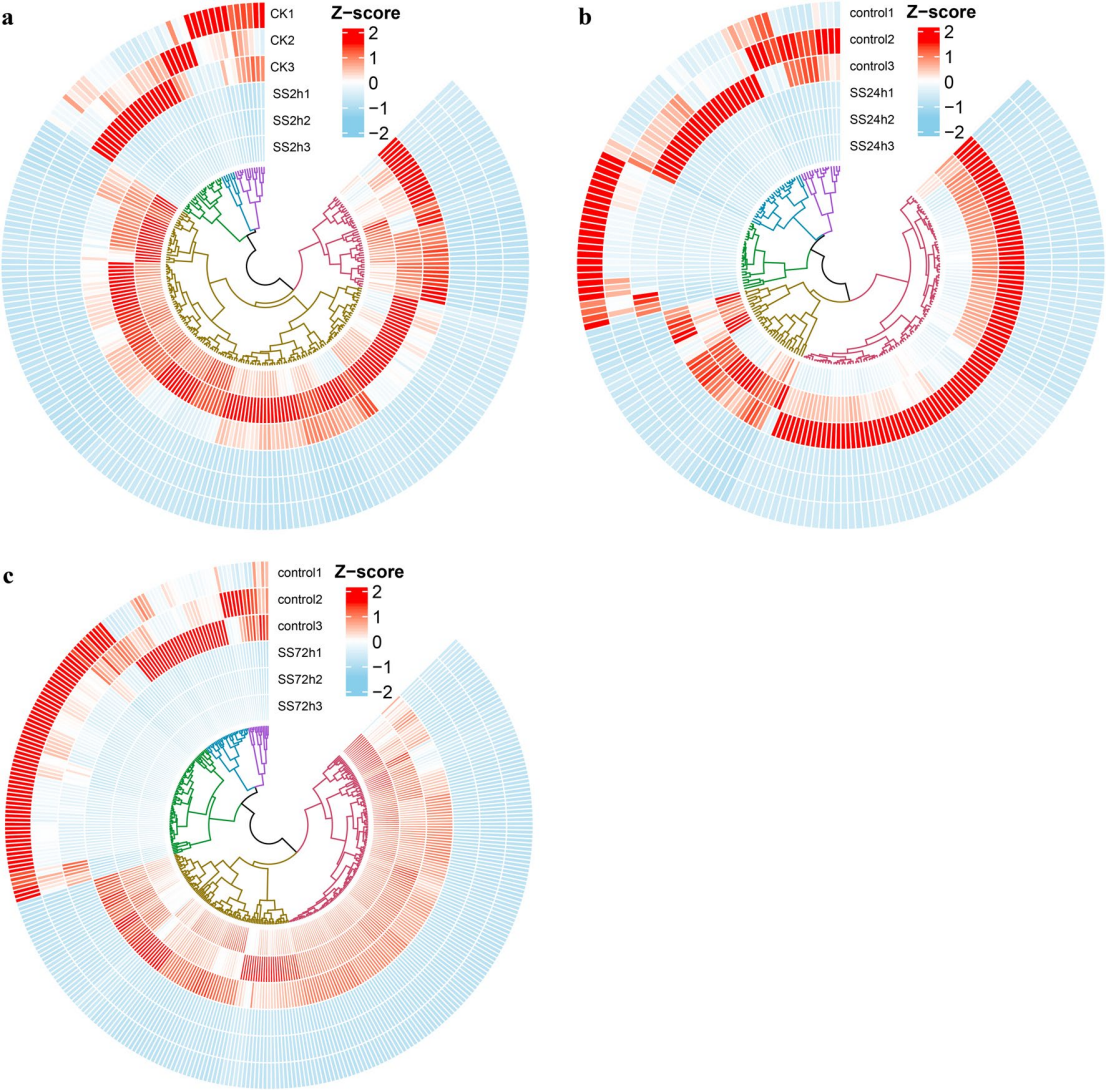
Hierarchical clustering analysis of DEGs with greater than sixfold change (Log_2_-transformed) in expression. (**a**) Hierarchical clustering analysis of differentially expressed genes (|log_2_FoldChange|> 6) in roots of canola seedlings treated with NaCl stress for 2 h. (**b**) Hierarchical clustering analysis of differentially expressed genes (|log_2_FoldChange|> 6) in roots of canola seedlings treated with NaCl stress for 24 h. (**c**) Hierarchical clustering analysis of differentially expressed genes (|log_2_FoldChange|> 6) in roots of canola seedlings treated with NaCl stress for 72 h.

Of the 16,758 DEGs, 5773 were unique, with 868, 866, and 1708 up-regulated unique DEGs and 381, 741, and 1209 down-regulated unique DEGs for NaCl stress at 2 h, 24 h, and 72 h, respectively, with 1066 up-regulated genes and 625 down-regulated genes showing differential expression in all three NaCl stress (Fig. 1c,d). Among the up-regulated DEGs in the root of NaCl-stressed canola seedlings, 1548 (46.51%), 1308 (50.86%), and 2018 (48.74%) genes showed a 2–threefold change (Log_2_-transformed) in expression at 2 h, 24 h, and 72 h of NaCl stress, respectively, while 185 (5.56%), 131 (5.09%), and 221 (5.34%) genes had more than sixfold changes (Log_2_-transformed) in expression; among the down-regulated DEGs, 1223 (59.46%), 923 (54.42%), and 1585 (53.46%) genes had 2–threefold (Log_2_-transformed) expression differences, while 40 (1.94%), 65 (3.83%), and 114 (3.85%) genes had > sixfold (Log_2_-transformed) expression differences, respectively (Fig. 1b). Hierarchical clustering analysis of DEGs with > sixfold changes (Log_2_-transformed) in gene expression showed high similarity in gene expression among biologically independent replicates of all treatments (Fig. 2), suggesting a high degree of replicability for each sample.

### Gene ontology analysis of DEGs

**Figure 3:**
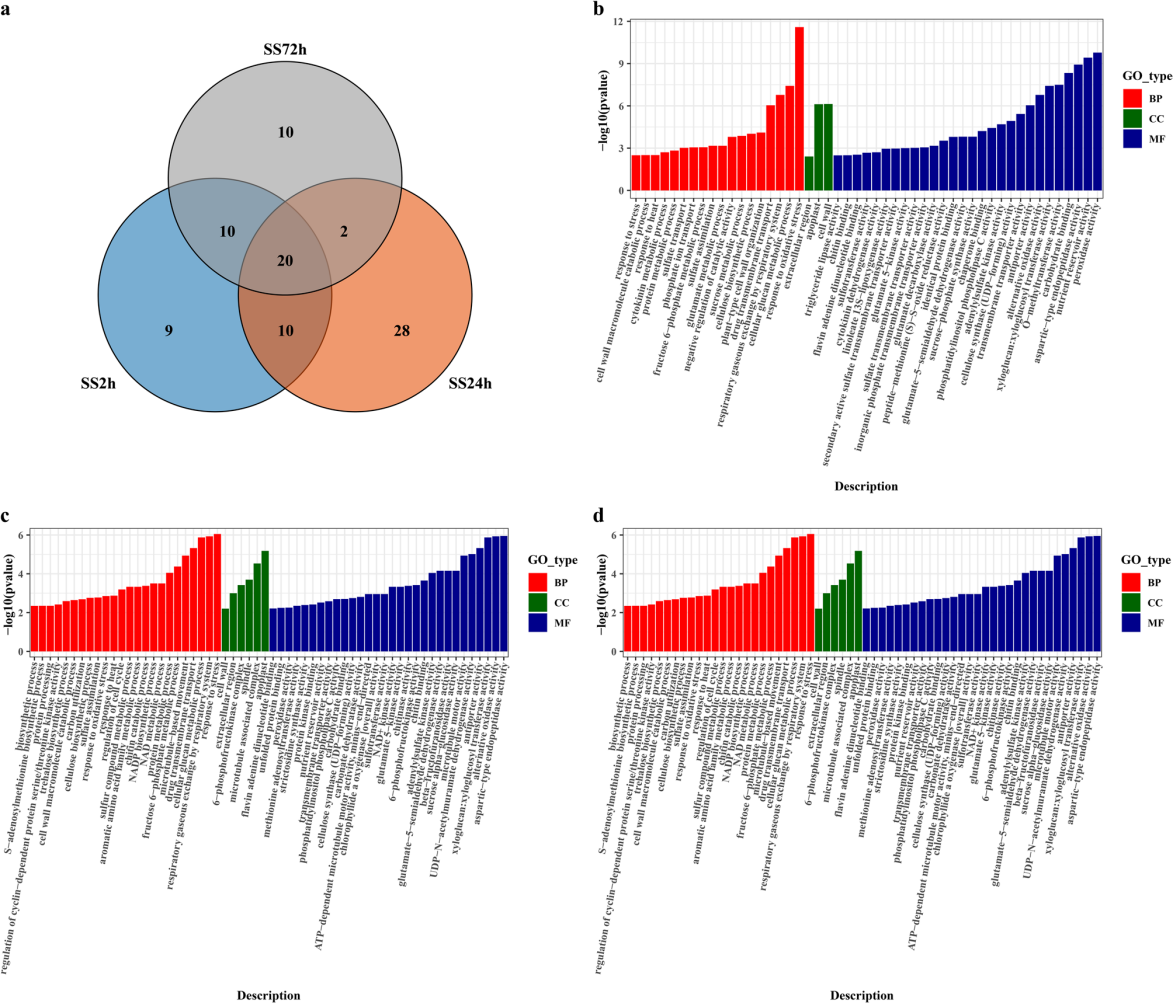
Gene ontology classification of DEGs between the three NaCl stress stages and the control. (**a**) Venn diagram showing the number of GO terms enriched in the root of canola at different NaCl stress times. (**b**) Analysis of GO enrichment in NaCl stress for 2 h. (**c**) GO enrichment analysis of NaCl stress for 24 h. (**d**) GO enrichment analysis of NaCl stress for 72 h. SS2h, NaCl stress for 2 h vs. control; SS24h, NaCl stress for 24 h vs. control; SS72h, NaCl stress for 72 h vs. control.

Differentially expressed genes (DEGs) were matched to *Brassica_napus* gene GO annotation data (*Brassica_napus*_GO.gz, https://www.genoscope.cns.fr/brassicanapus/data/GO/) using TBtools, followed by Gene Ontology (GO) enrichment analysis of the differentially expressed genes (DEGs) using the clusterProfiler R package. We found that 49, 60, and 42 GO terms were enriched under the 2 h, 24 h, and 72 h treatments of NaCl stress, respectively, with 9, 28, and 10 GO terms being unique at each corresponding stage (Fig. 3a). A total of 30 GO terms were commonly enriched for SS2h and SS24h, SS2h and SS72h were commonly enriched of 30 GO terms, SS24h and SS72h were commonly enriched of 22 GO terms, and 20 GO terms were found in all three NaCl stress. The number of GO terms enriched in the SS24h treatment (60) and the number of unique GO terms in the SS24h treatment (28) were significantly higher than in the other two treatments.

**Figure 4:**
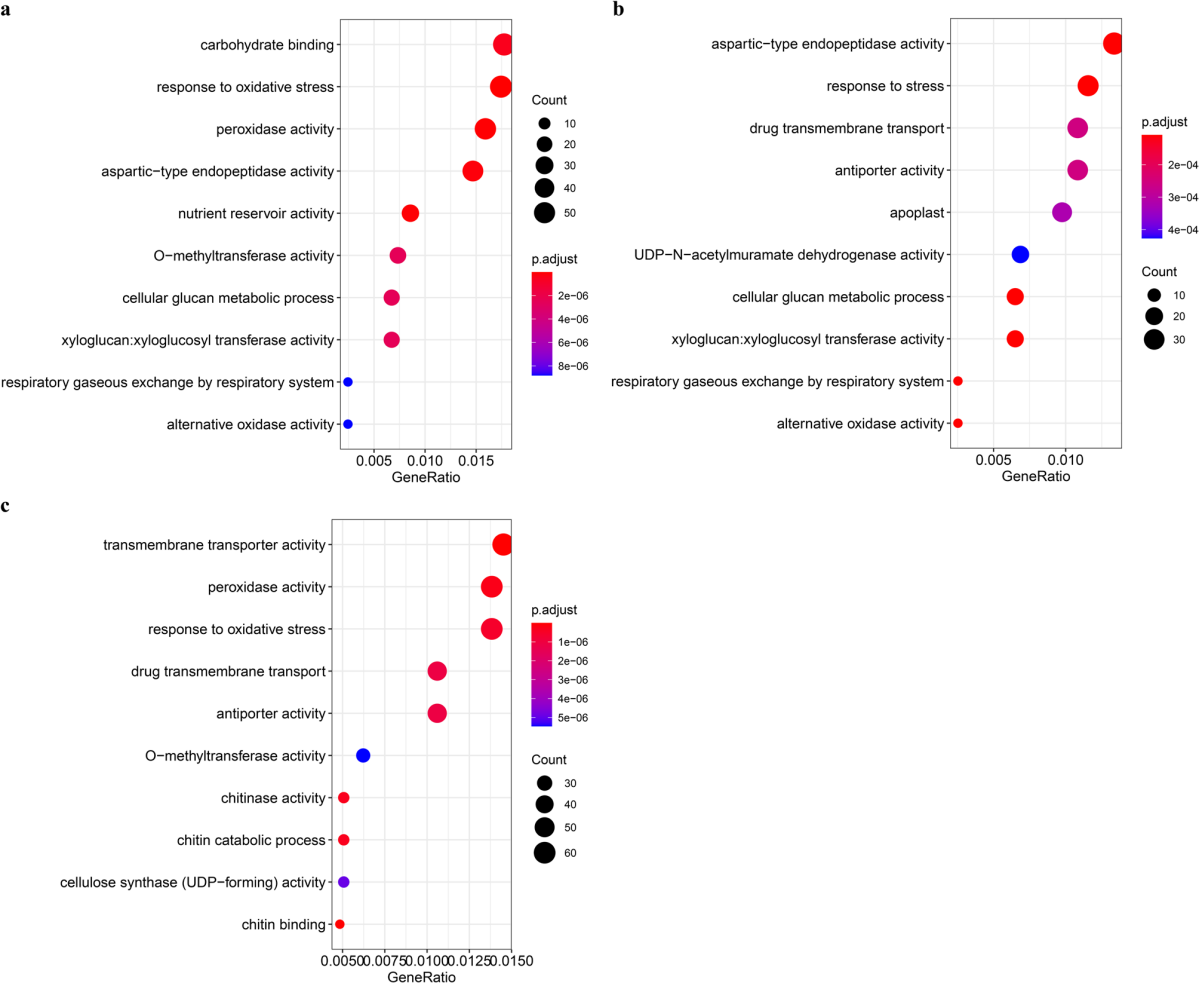
Dot plots of the top 10 GO terms in the GO enrichment analysis. (**a**) Dot plot of the top 10 GO terms in the GO enrichment analysis for the 2 h NaCl stress. (**b**) Dot plot of the top 10 GO terms in the GO enrichment analysis for the 24 h NaCl stress. (**c**) Dot plot of the top 10 GO terms in the GO enrichment analysis for the 72 h NaCl stress.

GO enrichment analysis of DEGs for all treatments showed that these annotated genes were classified into three main functional categories: biological process (BP), cellular component (CC) and molecular function (MF) (Fig. 3b,c,d), and the majority of transcripts from canola roots in all treatments were more enriched in the functional terms of biological process (18, 24, and 13 GO terms enriched for the 2 h, 24 h, and 72 h NaCl stress, respectively) and molecular function (28, 30, and 26 GO terms enriched for the 2 h, 24 h, and 72 h NaCl stress, respectively), but relatively few transcripts of cellular component (3, 6, and 3 GO terms enriched for the 2 h , 24 h, and 72 h NaCl stress, respectively) (Tables S2, S3, and S4). GO enrichment scatterplots show the top 10 (Padj) enriched functions for DEGs due to NaCl stress (Fig. 4). These results suggest that during the early stages of NaCl stress (SS2h), the enriched functions are mainly related to the response to oxidative stress and sugar metabolism (energy supply), during 24 h of NaCl stress the enriched functions are mainly related to the response to stress, alternating oxidase activity, sugar metabolism, transmembrane transport and cell membrane function, and during the late stages of salt stress (SS72h) the enriched functions are mainly related to transmembrane movement, sugar metabolism, amino acid metabolism, glycerol metabolism, and structural components of the cell wall.

### KEGG pathways of DEGs

**Figure 5:**
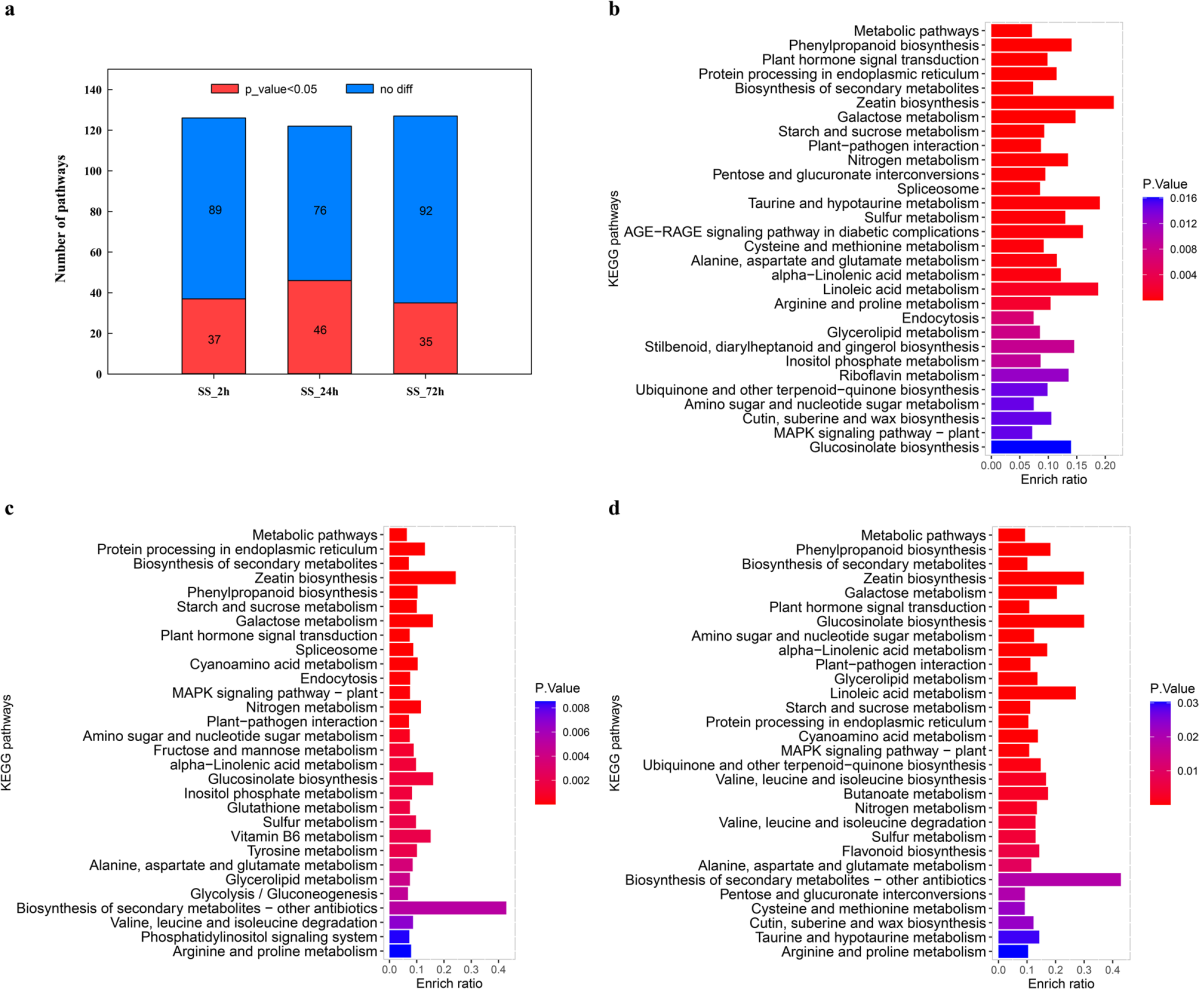
KEGG classification of differentially expressed genes (DEGs) in NaCl stressed canola roots. (**a**) Statistics on the number of KEGG pathways of differentially expressed genes in canola roots treated with NaCl stress at different times. (**b**) KEGG classification of DEGs in the comparison of NaCl stress at 2 h vs. control (top 30); (**c**) KEGG classification of DEGs in the comparison of NaCl stress at 24 h vs. control (top 30); (**d**) KEGG classification of DEGs in the comparison of NaCl stress at 72 h vs. control (top 30).

Gene enrichment analysis based on KOBAS 3.0 (http://kobas.cbi.pku.edu. cn/kobas3) revealed that 126, 122, and 127 KEGG pathways were enriched in canola roots exposed to NaCl stress at 2 h, 24 h, and 72 h (Tables S5, S6, and S7), respectively, with 37, 46, and 35 KEGG pathways were significantly enriched under NaCl stress at 2 h, 24 h, and 72 h, respectively (Fig. 5a). The top 30 significantly enriched pathways in NaCl-stressed canola roots (Figs. 5b,c,d, Tables S5, S6, S7) showed that the pathways with the most differentially expressed genes involvement were “Metabolic pathways (bna01100)” and “Biosynthesis of secondary metabolites (bna01110)”, followed by “Plant hormone signal transduction (bna04075)”, “Phenylpropanoid biosynthesis (bna00940)” and “Protein processing in endoplasmic reticulum (bna04141)”. Of the 37 significantly enriched pathways in 2 h NaCl-stressed canola roots, 26 pathways were dominated by up-regulated genes (Table S8). The 24 h NaCl stress significantly enriched 46 KEGG pathways in canola roots, 26 of which were dominated by up-regulated genes. Of the 35 significantly enriched pathways in 72 h NaCl-stressed canola roots, 25 pathways were dominated by up-regulated genes.

### Protein–protein interaction (PPI) analysis

**Figure 6:**
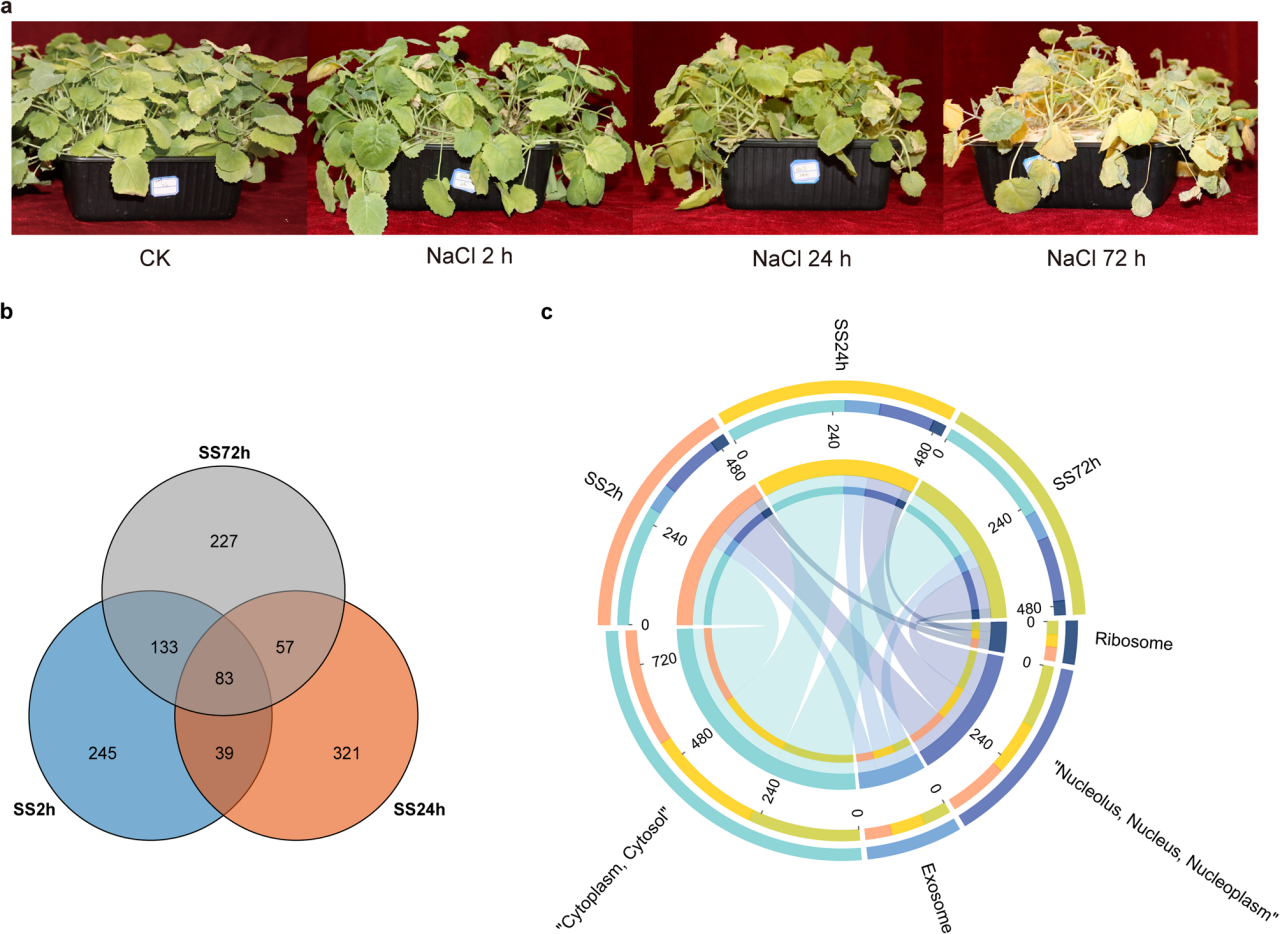
Veen plots and subcellular localization of the top 500 differentially expressed genes in roots of canola seedlings treated with 200 mM NaCl for 2 h, 24 h and 72 h. (**a**) Phenotypes of canola seedlings in control as well as in 200 mM NaCl NaCl stress for 2 h, 24 h and 72 h. (**b**) Venn diagram showing overlapping DEGs or unique DEGs in the top 500 differential genes for each NaCl stress time treatment. (**c**) Circos plot showing subcellular localization of the top 500 differential genes for each NaCl stress time points.

**Figure 7:**
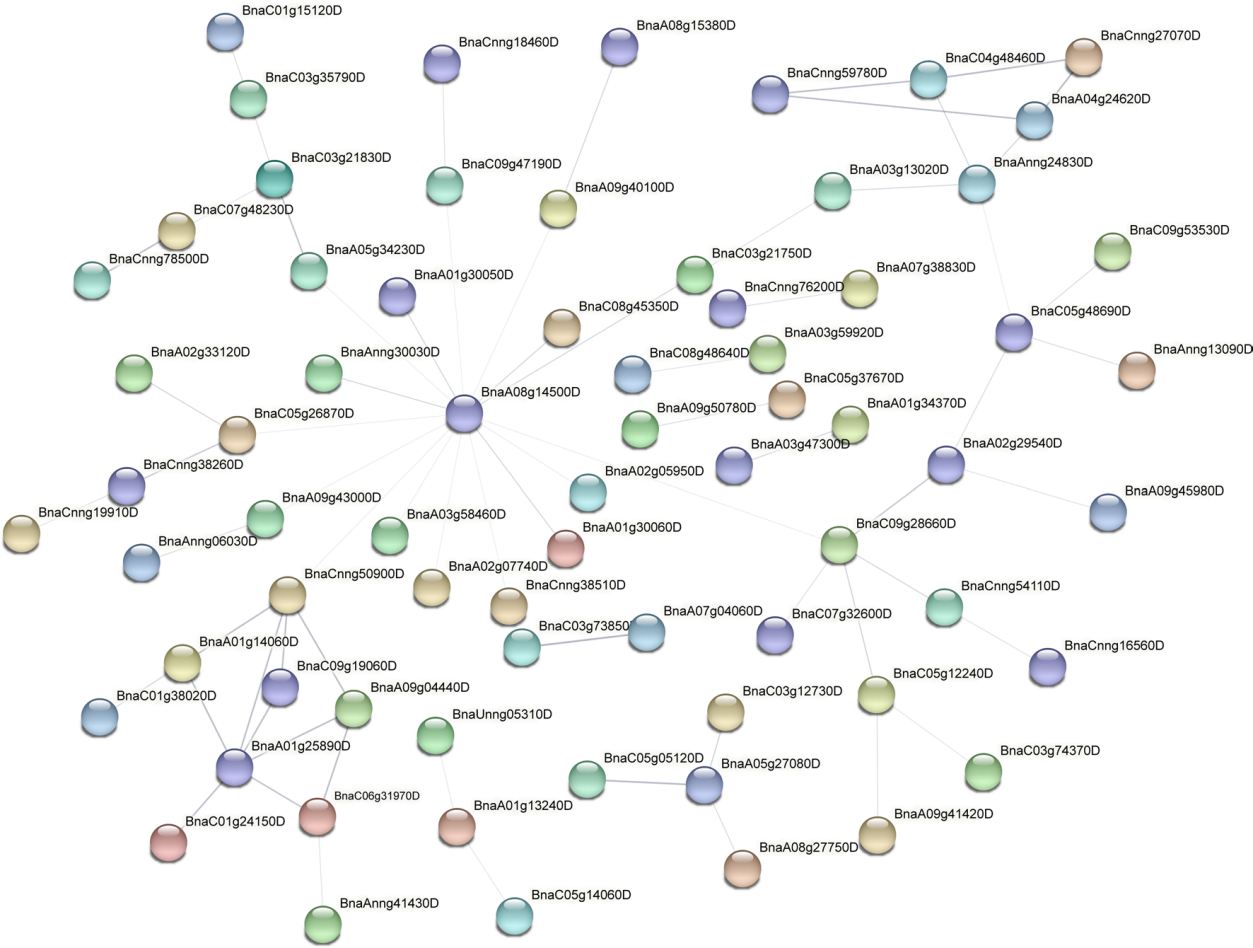
Protein–protein interaction (PPI) plots of the top 500 differentially expressed genes at 2 h of 200 mM NaCl treatment.

**Figure 8:**
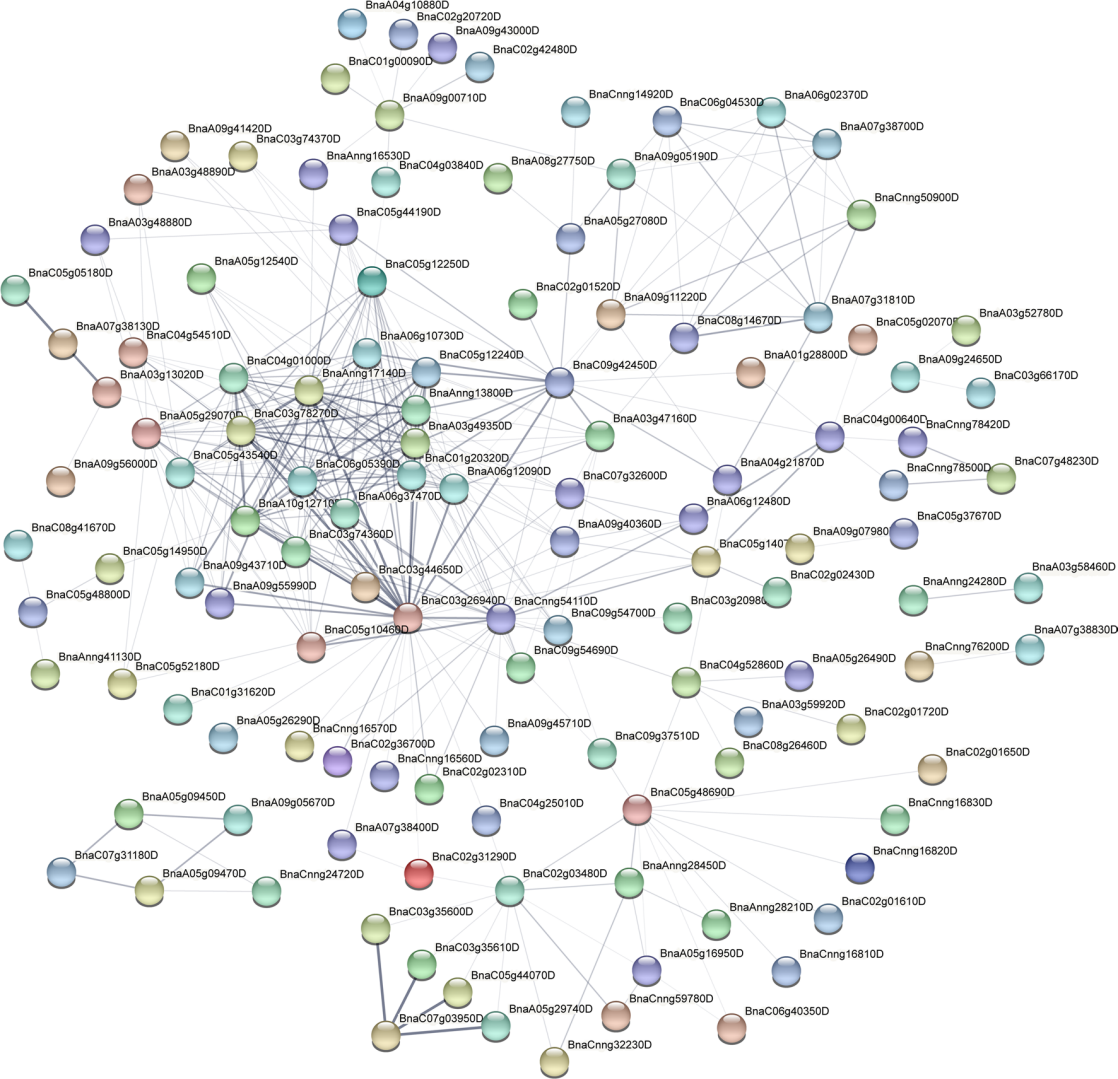
Protein–protein interaction (PPI) plots of the top 500 differentially expressed genes at 24 h of 200 mM NaCl stress.

**Figure 9:**
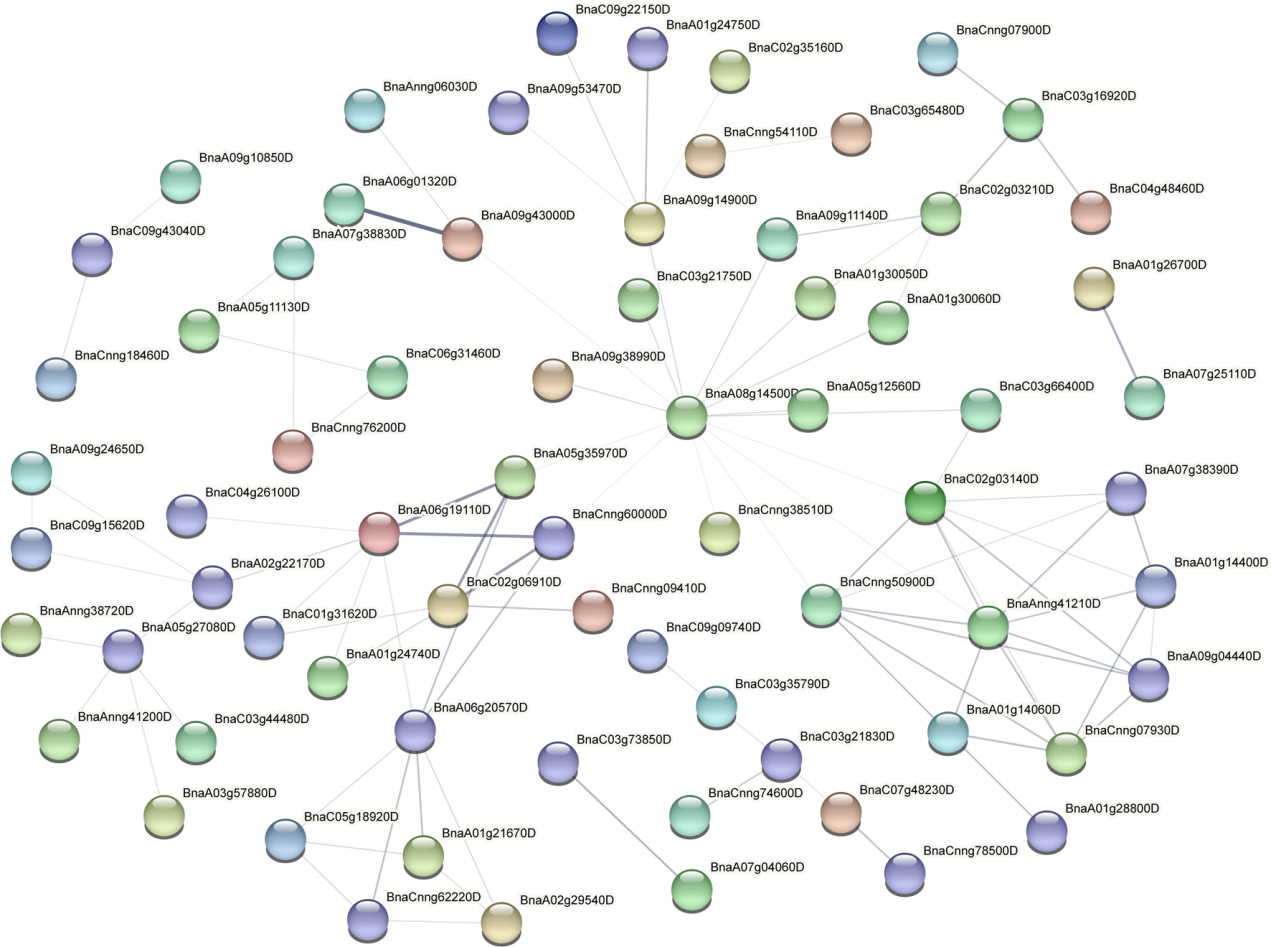
Protein–protein interaction (PPI) plots of the top 500 differentially expressed genes at 72 h of 200 mM NaCl stress.

To further analyse the function of differentially expressed genes, we screened the top 500 differentially expressed genes in the root systems of canola seedlings treated with 200 mM NaCl for 2 h, 24 h and 72 h. We found that 83 differentially expressed genes were common at the three time points of NaCl stress (Fig. 6b). Subcellular localisation of differentially expressed genes at the three time points showed that more than half of the genes were localised to the cytoplasm (54%, 54% and 52% at 2 h, 24 h and 72 h, respectively) (Fig. 6c). Furthermore, protein–protein interaction (PPI) analysis of the top 500 differentially expressed genes by STRING (https://string-db.org/) showed that *BnaA08g14500D* was located at the centre of the PPI network at 2 h of NaCl stress (Fig. 7). In addition, *BnaC09g28660D*, *BnaC09g50900D*, *BnaCnng50900D*, *BnaA01g25890D*, *BnaAnng24830D* genes were highly interconnected within the subnetwork. At 24 h of NaCl stress, *BnaC03g26940D*, *BnaAnng17140D* and *BnaC03g78270D* were located at the centre of the PPI network (Fig. 8). *BnaC09g42450D*, *BnaCnng54110D*, *BnaC04g01000D*, *BnaC05g12250D*, *BnaAnng17140D*, *BnaC05g12240D*, *BnaAnng13800D*, *BnaA03g49350D*, *BnaC01g20320D*, *BnaC06g05390D*, *BnaA06g37470D*, *BnaA10g12710D*, *BnaC03g74360D*, *BnaC05g10460D* genes were highly interconnected within the subnetwork. At 72 h of NaCl stress, *BnaA08g14500D* was located at the centre of the PPI network (Fig. 9); *BnaA06g19110D*, *BnaA06g20570D*, *BnaC02g03140D*, *BnaAnng41210D*, *BnaCnng50900D*, *BnaCnng07930D*, *BnaA09g04440D* genes were highly interconnected within the subnetwork, and were considered as possible key genes in the roots of canola seedlings in response to NaCl stress.

In addition, based on the DEGs identified in this study, we blasted these genes with assembled sequences on the NCBI website (https://blast.ncbi.nlm.nih.gov/Blast.cgi). At 2 h of NaCl stress, there were one hypersensitive-induced response protein homolog (*BnaC05g48690D*), two serine/threonine-protein kinase homologs (*BnaA04g24620D, BnaC04g48460D*), one CBL-interacting serine/threonine protein kinase homolog (*BnaAnng24830D*), one phosphomannomutase/phosphoglucomutase homolog (*BnaC06g31970D*) and one fatty acyl-CoA reductase homolog (*BnaA05g27080D*). At 24 h of NaCl stress, there were three serine/threonine-protein kinase homologs (*BnaA05g16950D, BnaCnng32230D, BnaC02g03480D*), one CBL-interacting serine/threonine-protein kinase homolog (*BnaCnng59780D*), one protein phosphatase 2C homolog (*BnaA03g13020D*), one E3 ubiquitin-protein ligase homolog (*BnaC05g10460D*), one fatty acyl-CoA reductase homolog gene (*BnaA05g27080D*), one aspartic proteinase nepenthesin homolog (*BnaC04g52860D*), three homologs related to sugar metabolism (sucrose synthase, *BnaA09g00710D*; galactinol–sucrose galactosyltransferase, *BnaC09g37510D*; bidirectional sugar transporter SWEET12, *BnaA09g05190D*), one pectate lyase homolog (*BnaC07g03950D*), one U-box domain-containing protein homolog (*BnaC03g74370D*) and one alpha-dioxygenase ( *BnaC05g48800D*). At 72 h of NaCl stress, the identified homologs included two ubiquinol oxidase homologs (*BnaA05g35970D, BnaCnng60000D*), one external alternative NAD(P)H-ubiquinone oxidoreductase homolog (*BnaC02g06910D*), one calcium-binding protein homolog (*BnaA06g20570D*), one serine/threonine-protein kinase homolog (*BnaC02g03210D*), one mitogen-activated protein kinase kinase kinase homolog (*BnaC03g16920D*), one fatty acyl-CoA reductase homolog (*BnaA05g27080D*), one alcohol dehydrogenase homolog (*BnaA09g43000D*), one peroxidase homolog (*BnaC03g21830D*), one mitochondrial phosphate carrier protein homolog (*BnaA06g19110D*), one inorganic phosphate transporter homolog (*BnaA02g22170D*) and one cytosolic sulfotransferase homolog (*BnaA09g14900D*).

### Changes of differential expression transcription factors (TFs) under salt stress

**Figure 10:**
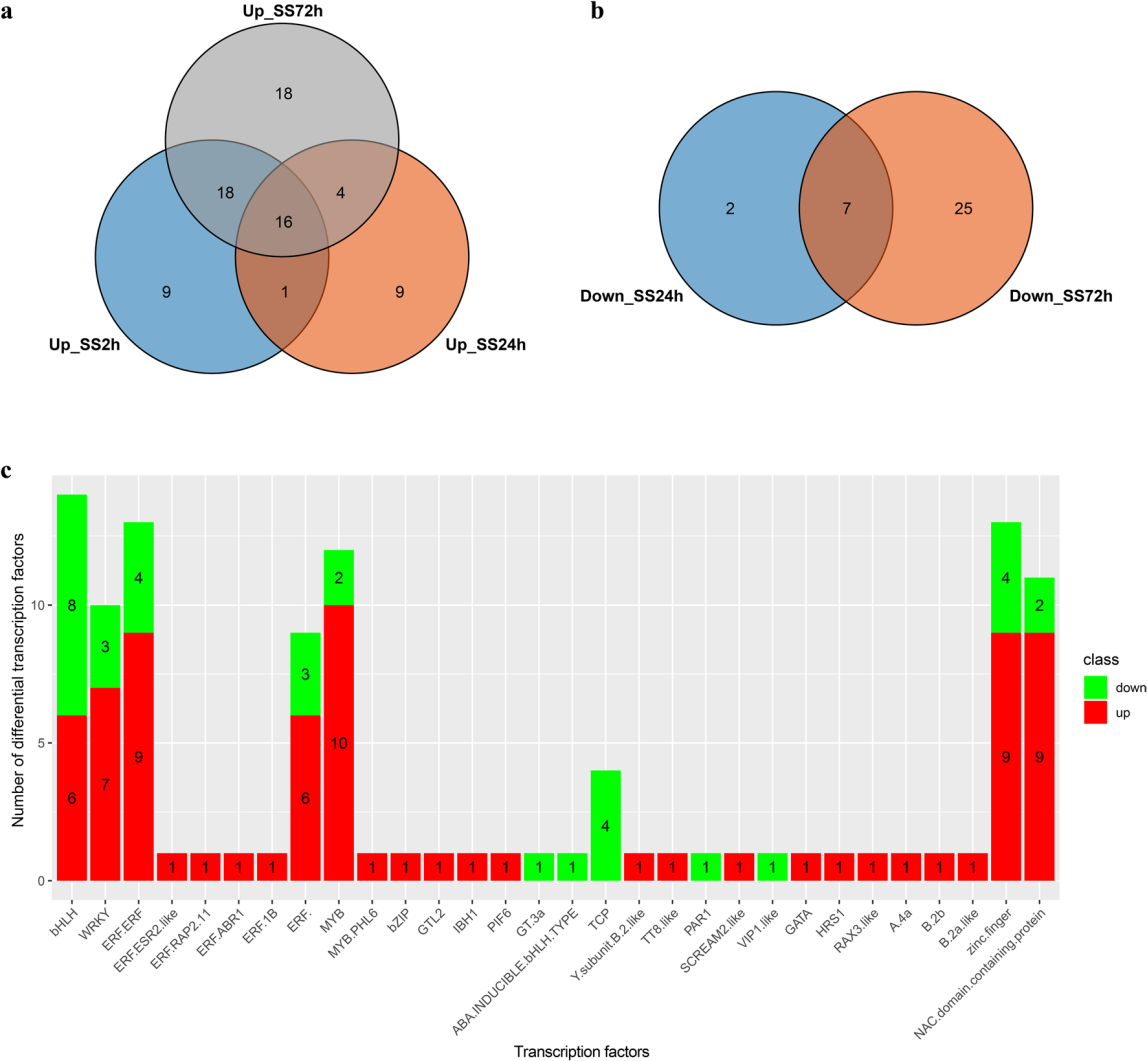
The distribution of differentially expressed transcription factors in canola roots under NaCl stress NaCl stress. (**a**) Venn diagram of the distribution of up-regulated transcription factors at different NaCl stress time points. (**b**) Venn diagram of the distribution of down-regulated transcription factors at different NaCl stress time points. (**c**) The histograms represent the number of up- and down-regulated transcription factors. SS2h, NaCl stress for 2 h vs. control; SS24h, NaCl stress for 24 h vs. control; SS72h, NaCl stress for 72 h vs. control.

Differentially expressed genes in roots of canola treated with NaCl stress were compared with the NCBI database (https://www.ncbi.nlm.nih.gov/gene/) using the R XML package, and 447, 698, and 1108 annotated differential genes were obtained at 2 h, 24 h, and 72 h of NaCl stress, respectively. Approximately 109 DEGs (75 up-regulated DEGs and 34 down-regulated DEGs) were identified as transcription factors (TFs) under NaCl stress. These transcription factors belonged to 24 transcription factor families (Fig. 10 and Table S9), and the families with the most transcription factors were ERF (26 transcription factors, including ERF-ERF, ERF-ESR2-like, ERF-RAP2, ERF-ABR1, ERF-1B, ERF-), followed by bHLH (14), MYB (13, including MYB, MYB-PHL6), zinc finger protein (13), NAC domain-containing protein (11), and WRKY (10) families. Most of the up-regulated TFs belonged to the ERF (19), MYB (11), zinc finger protein (9), NAC domain-containing protein (9), WRKY (7), and BHLH (6) families, and the most down-regulated TFs belonged to the bHLH (8) and ERF (7) families. To validate the results of the transcriptome data, 11 candidate DEGs (Table S10) were selected for quantitative reverse transcription-polymerase chain reaction (qRT-PCR) analysis of root samples treated with 200 mM NaCl for 2 h, 24 h, and 72 h and control (no salt stress), respectively. The results on the expression of the unigenes from qRT-PCR and RNA sequencing analysis were largely consistent (Figure S2). Our results confirmed the reliability of the transcriptome data which accurately reflected the response of canola roots to NaCl stress.

## Discussion

Salt stress leads to a rapid increase in ROS, including superoxide radicals (•O^2-^), hydrogen peroxide (H_2_O_2_) and hydroxyl radicals (•OH), which disrupt cellular redox homeostasis and lead to oxidative damage to many cellular components and structures^24^. Under salt stress, plants regulate ROS homeostasis in vivo through complex mechanisms such as enzymatic antioxidants (e.g., POD and CAT), non-enzymatic antioxidants e.g., glutathione (GSH)^25^. An et al.^26^ showed that glutathione S-transferase (GSTU) plays an important role in the antioxidant pathway, and previous studies have shown that GSTU could use GSH to reduce organic hydroperoxides of fatty acids and nucleic acids to the corresponding monohydroxy alcohols^27^. Dang et al.^28^ showed that the PrxR/Trx (peroxiredoxin/thioredoxin) defence system uses a thiol-based catalytic mechanism to reduce H_2_O_2_ and the genes encoding components of the PrxR/Trx defence system are considered to be candidate genes for salt resistance in plants. In this study, gene expression associated with peroxidase was up-regulated in all genes at early stage of salt stress (2 h), down-regulated at the intermediate stage (24 h) and late stage of salt stress (72 h); and gene expression associated with catalase up-regulated at 24 h of NaCl stress. Most of the genes associated with GSTUs were significantly up-regulated at both early (2 h) and late (72 h) stages of salt stress, and thioredoxin was more up-regulated at late stages of salt stress. The results for thioredoxin (*BnaA01g32310D*, *BnaC06g15930D*) and glutathione S-transferase (*BnaA06g11500D*, *BnaC02g24980D*, *BnaC03g30870D*, *BnaCnng19060D*, *BnaCnng35440D*, *BnaCnng76180D*) may be an important factor in the ROS scavenging system of canola roots.

Transcription factors (TFs) have previously been shown to play integral roles in regulating ion, osmotic and ROS homeostasis in plants in response to salt stress^12,29–31^. Previous studies have shown that WRKY responds to salt stress through ABA signaling and regulates ROS production in plant cells^10^, in addition, Wang et al.^32^ reported that the bZIP gene (ThbZIP1) improves salt resistance in tobacco by preventing the accumulation of cellular ROS. Overexpression of the B-box zinc finger protein MdBBX10 in Arabidopsis enhanced the ability of plants to scavenge reactive oxygen species under adversity, thereby enhancing tolerance to salt stress in Arabidopsis^33^. Previous studies have shown that MYB transcription factors enhance tolerance to salt and drought stress in crops by scavenging ROS and inhibiting cell membrane damage^19^, as well as regulating ion homeostasis^20^ and cuticle formation^21^. In addition, Wu et al.^30^ also showed that MYB transcription factors enhanced tolerance to drought and salt stress through an ABA-dependent pathway. Samira et al.^34^ showed that overexpression of the gene encoding bHLH (IAALEUCINE RESISTANT3) increased salt tolerance in *Arabidopsis*. Krishnamurthy et al.^35^ also showed that the transcription factor bHLH regulates the AtNHX gene in arabidopsis to mediate salinity responses. NAC may regulate the salinity response in soybean by activating the DREB/CBF-COR pathway to regulate salt stress tolerance in leaf and root of soybean and may control lateral root development by altering growth hormone signalling-related genes^29^. In the present study, the MYB- and NAC- encoding genes in canola roots were significantly up-regulated at 2 h, 24 h, and 72 h of NaCl stress (Table S9), indicating the important role of MYB and NAC TF families in the salt stress response of *Brassica napus*. Our results were consistent with the findings of Wang et al.^36^ showing that transcription factors such as MYB, NAC and AP2-EREBP, which act as major switches controlling downstream gene expression, are highly upregulated in industrial rapeseed (*Brassica napus*) under NaCl stress. Furthermore, we found differences in the expression profiles of transcription factors in the roots of canola seedlings at different times of NaCl stress, with transcription factors of the ERF, bHLH, MYB, zinc finger protein, NAC, WRKY, and HSF families all significantly up-regulated at 2 h of NaCl stress. In contrast, ERF and bHLH family transcription factors were significantly down-regulated at 24 h of NaCl stress, and bHLH, zinc finger protein, WRKY and TCP family transcription factors were significantly down-regulated under 72 h of NaCl stress. This suggests that these transcription factors play as positive regulators of plant defence in short-term stress, while the down-regulation of some transcription factors such as bHLH, zinc finger protein, WRKY, and TCP at 24 and 72 h of NaCl stress, suggests that these transcription factors may function as negative regulators of salt tolerance in canola. Our results were consistent with the down-regulation of zinc finger proteins in Caragana korshinskii under salt stress^37^. These findings support the possibility that zinc finger proteins, WRKY, may play a key role as positive and negative regulators of plant defence.

Abscisic acid (ABA) plays an important regulatory role in plant salt stress defence as a stress response hormone and plays a key role as a growth inhibitor^38–40^. In response to salinity and osmotic stress, endogenous ABA levels increase rapidly and the abscisic acid receptors PYR/PYL/RCARs bind to ABA and inhibit protein phosphatases 2C (PP2Cs), thereby activating SnRK2s (protein kinases). snRK2s phosphorylate ABA response transcription factors (AREB/ABFs), which in turn regulate stomatal closure in plants in response to osmotic stress^39^. It has also been shown that the downstream targets of ABA transcription are lipid metabolism and lipid transfer proteins, suggesting an interplay between ABA-dependent signaling and lipid metabolism pathways that work together to maintain the structural and functional integrity of the cell membrane^41^. Previous studies on metabolites in canola roots treated with 200 mM NaCl stress for 72 h by metabolomics also showed that lipids (including unsaturated fatty acids, glycerophospholipids, lecithin, and sphingolipids) were significantly increased under NaCl stress and were the most abundant metabolites increased in response to NaCl in canola roots^42^. Lipids are essential components of biological membranes and play an important role in ion transport and signal transduction^43^. Under salinity conditions, lipids affect membrane permeability, fluidity, integrity and the activity of transport proteins^44^. In addition, lipid metabolic reorganization is an important process for salt tolerance in plant cells^44–46^. Salt stress-induced production of polyunsaturated fatty acids (linoleic acid, oleic acid, and α-linolenic acid) contributes to the activation of Arabidopsis PM H^+^- atpase activity^47^, these studies suggest that lipid metabolism plays an important regulatory role in plant salt tolerance. PHOSPHOLIPASE Dα1 (PLDα1) functions as an ABA-regulated lipid messenger PA biosynthesis and ACBP1 (acyl-CoA binding protein 1) regulates PLDα1 expression^48^, suggesting that the regulation of cellular lipid structure is critical for the regulation of abiotic stress-related ABA signaling^28,48^. In the present study, some genes involved in ABA (epoxycarotenoid dioxygenase) and hormone signaling (TFs such as zinc finger, ERF, and gibberellin-regulated protein) were up-regulated under NaCl stress (Table S11). The 9-cis-epoxycarotenoid dioxygenase (e.g. *BnaA07g39250D*, *BnaCnng38510D*), genes involved in ABA synthesis, were significantly up-regulated in both the early (2 h) and late (72 h) stages of salt stress. In addition, the “abscisic acid receptor” (*BnaA07g38130D*, *BnaC05g00620D*, *BnaCnng68710D*) and “protein phosphatase 2C” (*BnaC01g18020D*) genes involved in the ABA signaling pathway were significantly up-regulated in both early (2 h) and late (72 h) stages of salt stress, which were consistent with the previous reports in winter rapeseed^17^.

In the GA signaling pathway, the gibberellin receptor GID1 protein binds to active gibberellin (GA), senses and transmits GA signals, and thus induces a series of downstream responses^49^. The GID1 protein can be ubiquitinated by the E3 ubiquitin ligase GARU (GA receptor RING E3 ubiquitin ligase) and degraded via the proteasome pathway^50^. The E3 ubiquitin ligase F-box protein SLEEPY1 (SLY1) interacts with DELLAs, leading to the degradation of DELLAs, thereby relieving the inhibitory effect of DELLA proteins and promoting plant growth and development^39,51^. In the present study, gibberellin-regulated proteins were significantly down-regulated in canola roots in the 24 h and 72 h of NaCl stress, while E3 ubiquitin ligase F-box proteins were significantly up-regulated in all three periods of salt stress, indicating that canola restricted plant growth by reducing GA levels under salt stress^39^. Feng et al.^52^ also showed higher expression levels and fold changes of genes involved in ABA biosynthesis through transcriptomic analysis of allotetraploid rapeseed under salt stress.

**Figure 11:**
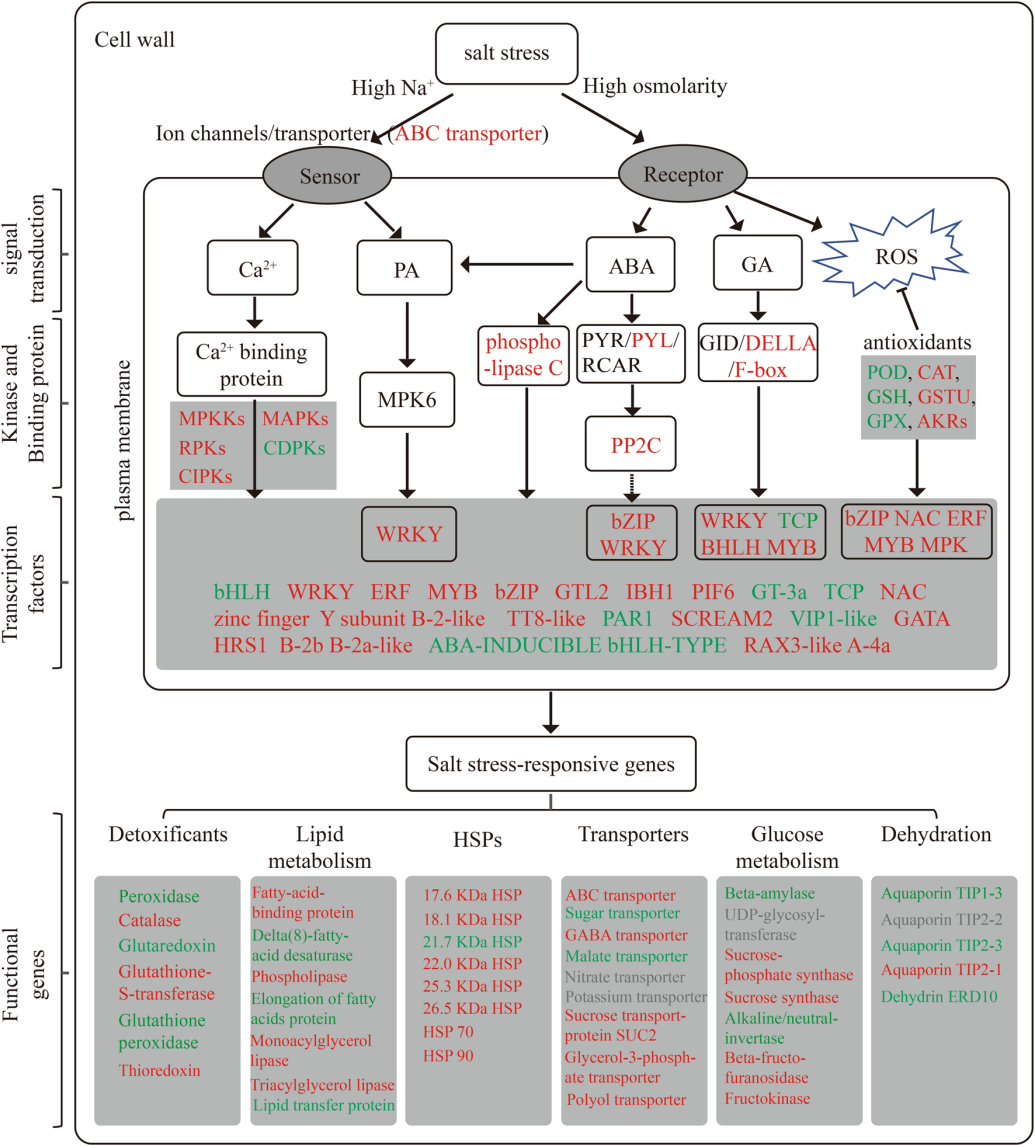
Possible molecular mechanisms of canola response to salt stress. Red font indicates that the gene was expressed up-regulated in all treatments or predominantly up-regulated, blue font indicates that the gene was predominantly down-regulated, and grey font indicates that the gene was up-regulated or down-regulated in the same number of genes in all treatments.

Furthermore, calcium-mediated up-regulation of various signaling pathways such as mitogen-activated protein kinase (MPKK), serine/threonine protein kinase (MAPK), receptor protein kinase (RPK) and CBL-interacting serine/threonine protein kinases (CIPKs) were also identified as regulators of salinity responses^40,53^. The calcium-mediated upregulation of various signaling pathways in this study, such as mitogen-activated protein kinases (e.g. *BnaA02g09290D*, *BnaC09g24030D*), serine/threonine protein kinases (e.g. *BnaA05g16950D*, *BnaCnng07900D*, *BnaC05g29750D*, *BnaC05g43810D BnaCnng07900D*), receptor protein kinases (e.g. *BnaC02g00270D*), and CBL-interacting serine/threonine protein kinases (e.g. *BnaC07g29570D*) were more up-regulated in both early (2 h) and late (72 h) stages of salt stress. Li et al.^37^ reported that in *Caragana korshinskii* seedlings, a large number of protein kinases may play crucial roles in sensing external drought and salt signals, and regulating gene expression at the cellular level, with S-receptor-like serine/threonine protein kinase GsSRK significantly increased the expression under Neutral salt (NaCl) and alkaline salt (NaHCO_3_) treatments. This is consistent with the results of the present study. The mitogen-activated protein kinase (MPKKs) cascade mediates the balance of ions, osmosis and ROS has been demonstrated in many studies^54^. Previous studies have shown that gene regulation under abiotic stress is influenced by multiple transcriptional cascades^14–16^, in which transcription factors may first be activated by upstream protein kinases (e.g. CDPK ) to regulate downstream target gene expression in response to stress^14^. WRKY transcription factors have been shown to play an important role in inhibiting the gibberellin (GA) signaling pathway and activating the abscisic acid (ABA) signaling pathway^15^. The regulatory functions of WRKY33 and bHLH122 in ROS detoxification by targeting peroxidase and glutathione-S-transferase suggest a function for transcription factors in linking ROS scavenging to osmotic stress and oxidative stress-induced signaling^14,24^. Studies on the saline plant *C. glaucum*^55^ further revealed the phosphorylation of CgbHLH001 as a substrate of CgCDPK, and the N-terminal interactions confirmed that the transcription factor CgbHLH001 may be regulated by post-translational modification (protein kinases such as CDPK phosphorylation) and enhances stress tolerance by controlling downstream related genes to enhance the scavenging of excess ROS. Passricha et al.^56^ also showed that overexpression of PsLecRLK (protein kinase) can increase the activity of ROS scavenging enzymes, reduce the ROS-induced membrane damage, and show enhanced tolerance to salt stress. Furthermore, MPK6-mediated phosphorylation of the plasma membrane Na^+^/H^+^ antiporter SOS1 is directly regulated by NaCl and phosphatidic acid (PA), supporting the relationship between lipid and MAPK signaling in response to plant salt stress^57^. Yue et al.^58^ also demonstrated an interaction between AtMPK6 (MPKK) and phosphatidic acid (PA), mediating the activity of the SOS1 Na^+^/H^+^ antiporter under salinity stress. These results suggest that the crosstalk between the above pathways regulates salt tolerance in canola. A putative model culminating our observations was presented in Fig. 11.

In summary, transcript expression in canola roots differed at different stages of salt stress. It was mainly related to oxidative stress response and sugar metabolism (energy supply) with the early stage of NaCl stress (2 h); while it was primarily related to stress response, alternating oxidase activity, sugar metabolism, transmembrane transport and cell membrane function at 24 h of NaCl stress. At late salt stress (72 h), it was more related to transmembrane movement, sugar metabolism, amino acid metabolism, glycerol metabolism and structural components of the cell wall. A number of differential expressed genes enriched in plant signal transduction pathways were highly expressed in the early (2 h) and late (72 h) stages of salt stress, and these genes play important roles in signaling and scavenging of ROS in canola in response to NaCl stress. In addition, several families of transcription factors possibly associated with salt tolerance were identified, including ERF, MYB, zinc finger protein, NAC, WRKY, and bHLH. Although more work is needed to elucidate the specific functions of these identified genes, our findings will contribute to the further identification of key genes for salt tolerance in canola.

## Methods

### Plant materials

Based on pre-experimental screening, the canola (*Brassica napus* L.) variety cv. Huayouza No. 62 is salinity tolerant and was therefore used as the material in this experiment. Seeds were provided by Huazhong Agricultural University, China. All experiments on these materials were performed in accordance with relevant institutional, national and international guidelines and legislation.

### Seedling culture and hydroponic experiment

Based on pre-experiments of seed germination tests and hydroponic trials of canola seedlings under salt stress, we screened 200 mM NaCl as the salt stress concentration for hydroponic trials of canola seedlings. The specific steps of the canola seedling culture and hydroponic test were as follows: uniformly sized seeds were selected and placed in 96-well plastic germination trays filled with vermiculite for germination, one seed per hole, with an incubation temperature of 25 °C and a light period of 14 h. 7 days later, seedlings of uniform size were randomly selected from the germination trays and transplanted into pots (13.5 cm diameter at the top, 10.5 cm diameter at the bottom and 12.5 cm height) filled with vermiculite, with 6 seedlings per pot. Every two days they were watered with 100 ml of 1/4 strength Hoagland nutrient solution. When the seedlings were at the three true-leaf stage, they were transferred to hydroponics with 1/4 strength Hoagland nutrient solution, which was changed every other day. When seedlings were at the five true-leaf stage, the nutrient solution was replaced with 1/2 strength Hoagland nutrient solution. After 3 days of acclimatisation, treatments were carried out: control (1/2 strength Hoagland nutrient solution) and 200 mM NaCl (the salt solution was prepared with 1/2 strength Hoagland nutrient solution, NaCl concentration in the nutrient solution increased by 50 mM every 24 h until the concentration of NaCl in the nutrient solution reached 200 mM) salt stress for 2 h, 24 h, and 72 h (Referring to the results of Yao et al.^59^ and Shao et al.^60^, as the short-term (1 h, 2 h) salt stress was a fast-acting physiological response and immediate, with more up-regulated DEI than down-regulated DEI, stabilized at 24 h and with a slowed metabolic regulatory response; at the later stages of salt stress (72 h), the down-regulated DEI increased significantly. In our preliminary experiments, we also found significant change in shoot morphology after 2 h (at early stage), 24 h (at intermediate stage) and 72 h (at late stage) of 200 mM NaCl stress as added in Fig. 6a. Therefore, we sampled roots for transcriptome analysis at those three time points, and the samples without salt stress were used as the control). 12 replicates (12 pots) were set up for each treatment. After 2 h, 24 h, and 72 h of incubation at the final salt concentration (200 mM NaCl), the roots of the treatment and control groups were quickly washed with Milli-Q water (the collection of roots from all treatments was collected at the same time). Roots from 6 plants were collected from each pot, pooled into one replicate and rapidly frozen in liquid nitrogen and stored at − 80 °C for transcriptome sequencing analysis. Three such replicates were used for transcriptomic analysis (3 × 6 × 4 = 72 plants in total).

### RNA isolation and sequencing

RNA was isolated and sequenced by reference to the method of Wang et al.^6^. Briefly, total RNA was extracted from canola root tissue using TRIzol reagent according to the manufacturer's instructions. mRNA was purified from total RNA using poly-T oligo-attached magnetic beads and fragmented using divalent cations in NEBNext first-strand synthesis reaction buffer. First-strand cDNA was synthesized using random hexamer primers and M-MuLV reverse transcriptase. second-strand cDNA synthesis was subsequently performed using DNA polymerase I and RNase H. The purified double-stranded cDNA product was processed through magnetic beads, end-repaired and ligated to the aptamer. Transcriptome libraries were generated using the NEBNext® Ultra™ RNA Library Prep Kit for Illumina® (NEB, USA) and sequenced by the Illumina HiSeq 2500 platform. Low quality reads were removed from the resulting raw data (raw reads) in fastq format to obtain clean data (clean reads). All downstream analyses were based on high quality clean data. Referring to the method of Wang et al.^6^, these clean reads were compared to the reference genome sequence (Brassica_napus genome v4.1: https://www.genoscope.cns.fr/brassicanapus/data/) using the Hisat2 tool software. The two groups were also analysed for differential expression using DEseq, and corrected p-values (padj) < 0.05 and absolute values of fold change (|log_2_Fold Change|) > 2 were then used as thresholds for screening differentially expressed genes (DEGs). Selected DEGs were matched against the *Brassica napus* gene annotation database (http://www.genoscope.cns.fr/brassicanapus/data/Brassica_napus.annotation_v5.cds.fa.gz) using TBtools. Gene ontology (GO) enrichment analysis of DEGs was achieved by the GOseq R package, and Kyoto Encyclopedia of Genes and Genomes (KEGG) pathway analysis and functional annotation of DEGs was performed using KOBAS 3.0 (https://kobas.cbi.pku.edu.cn).

### Validation of RNA-Seq data

Based on the results of RNA expression profiles, we screened for differentially expressed genes (DEGs) under NaCl stress at 2 h, 24 h and 72 h. To validate the accuracy and reproducibility of the transcriptome data analysis, we screened for differentially expressed genes common to NaCl stress at 2 h, 24 h and 72 h. For the reliability of the data, we randomly selected 11 genes that were significantly differentially expressed at all time points under NaCl stress for qRT-PCR analysis to validate the results of the RNA-Seq data (see Table S10 for a list of genes and primers). qRT-PCR validation procedures used the same extracted RNA as for the transcriptome. cDNA for qRT-PCR was synthesized by using MMLV reverse transcriptase (TaKaRa, Dalian, China) synthesized from 2.5 µg of total RNA. The qRT-PCR assays were performed using the SYBR Premix Ex Taq II Kit (TaKaRa, Dalian, China) on a 7500 Fast Real-Time PCR System (Applied Biosystems, USA). Three technical replicates were performed for each sample. The ACTIN (Table S10) was used as an internal control. Relative gene expression levels were calculated using the 2^−△△Ct^ method^61^.

### Data analysis

Data were analysed using Microsoft Excel (Microsoft Corp., Redmond, WA, USA) and R (4.0.3, Vienna, Austria. URL https://www.R-project.org/). Differentially expressed genes (DEGs) were annotated using the clusterProfiler R package, gene ontology (GO) enrichment analysis of DEGs was performed using the GOseq R package, and graphing was performed using the ggplot2 and circlize R packages.

## Supplementary Information

Supplementary Information 1.

Supplementary Information 2.

## Data Availability

All Gene ID and annotation files could be obtained from *Brassica napus* genome v4.1 (https://www.genoscope.cns.fr/brassicanapus/data/). Raw data and other data generated or analyzed were included in this published article in this study (Table S1, Table S2, Table S3, Table S4, Table S5, Table S6, Table S7, Table S8, Table S9, Table S10, Table S11, Figure S1, Figure S2). The datasets used and/or analysed have been deposited in the National Center for Biotechnology Information (NCBI) Sequence Read Archive (SRA, https://www.ncbi.nlm.nih.gov/sra). The accession number is PRJNA721127 (https://www.ncbi.nlm.nih.gov/bioproject/PRJNA721127), which includes 21 accession items (SAMN18700206 – SAMN18700226).

## Abbreviations

SS2h: NaCl stress (200 mM) for 2 h vs. control
SS24h: NaCl stress (200 mM) for 24 h vs. control
SS72h: NaCl stress (200 mM) for 72 h vs. control
DEGs: Differentially expressed genes
qRT-PCR: Quantitative real-time polymerase chain reaction
RNA-seq: RNA sequencing
GO: Gene ontology
KEGG: Kyoto Encyclopedia of Genes and Genomes
TF: Transcription factor
MPKKs: Mitogen-activated protein kinase
MAPKs: Serine/threonine-protein kinase
RPKs: Receptor-like protein kinase
CDPKs: Calcium-dependent protein kinase
CIPKs: CBL-interacting serine/threonine-protein kinase
PP2C: Protein phosphatase 2C
PYL: Abscisic acid receptor
POD: Peroxidase
CAT: Catalase
GSH: Glutaredoxin
GSTU: Glutathione S-transferase
GPX: Glutathione peroxidase.
AKRs: Aldo–keto reductase
