# Supplementary material for: Salt‑responsive transcriptome analysis of canola roots reveals candidate genes involved in the key metabolic pathway in response to salt stress
Source: Sci Rep. 2022 Jan 31;12:1666. doi: 10.1038/s41598-022-05700-2 (PMC8803978; doi:10.1038/s41598-022-05700-2)
Supplement: Supplementary file 1 — Supplementary Information 1. [file 41598_2022_5700_MOESM1_ESM.docx]

**Figure S1** Volcano plot of differentially expressed genes (DEGs) in canola roots exposed to different times of salt stress.

**Figure S2** qRT-PCR validation of transcript levels evaluated by RNA-Seq in canola roots under 200 mM NaCl stress.


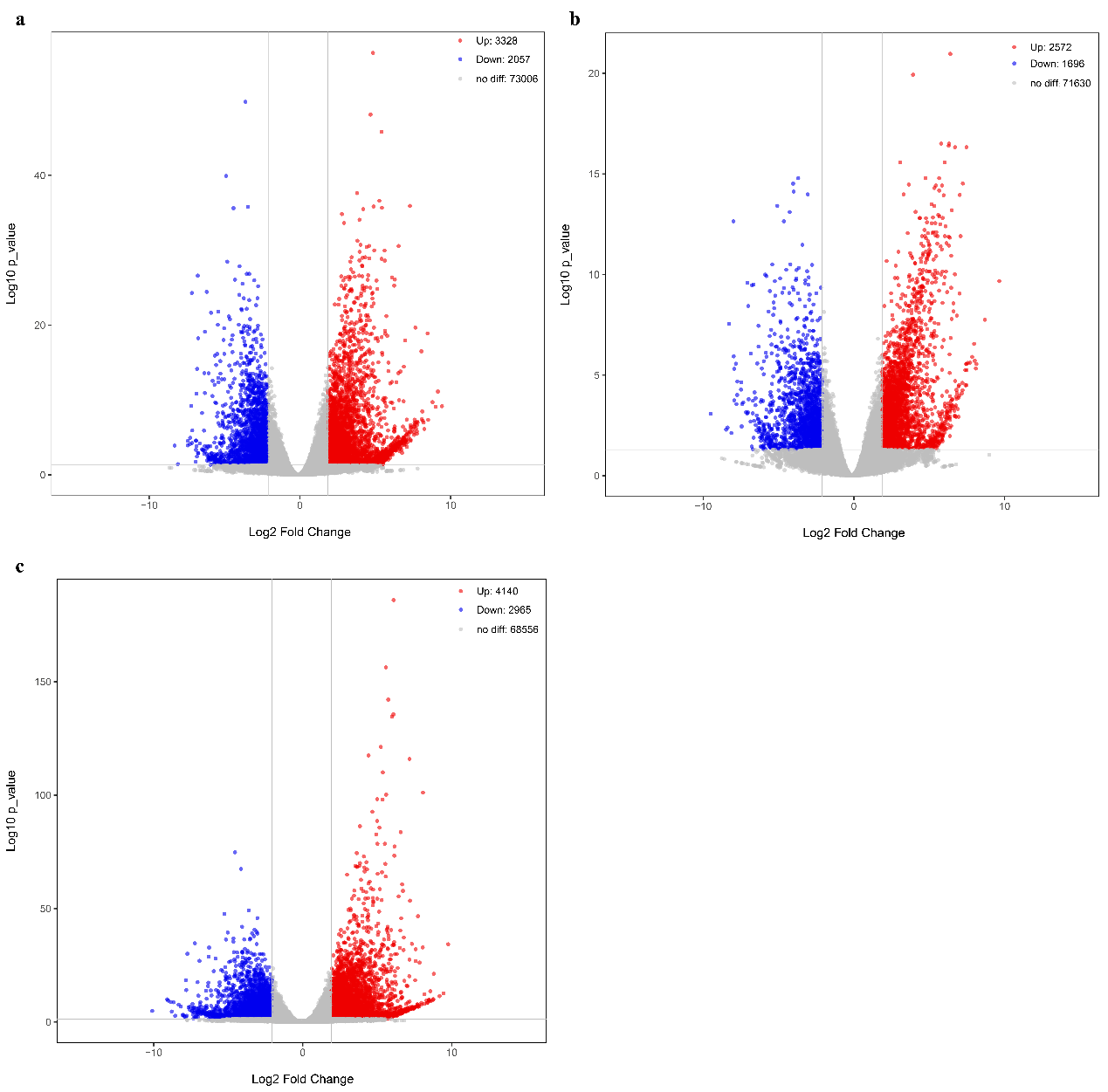


Fig. S1 Volcano plot of differentially expressed genes (DEGs) in canola roots exposed to different times of salt stress. **(a)** Volcano plot of DEGs between the 2 h NaCl stress treatment and the control (0 h). **(b)** Volcano plot of DEGs between the 24 h NaCl stress treatment and the control (0 h). **(c)** Volcano plot of DEGs between the 72 h NaCl stress treatment and the control (0 h).


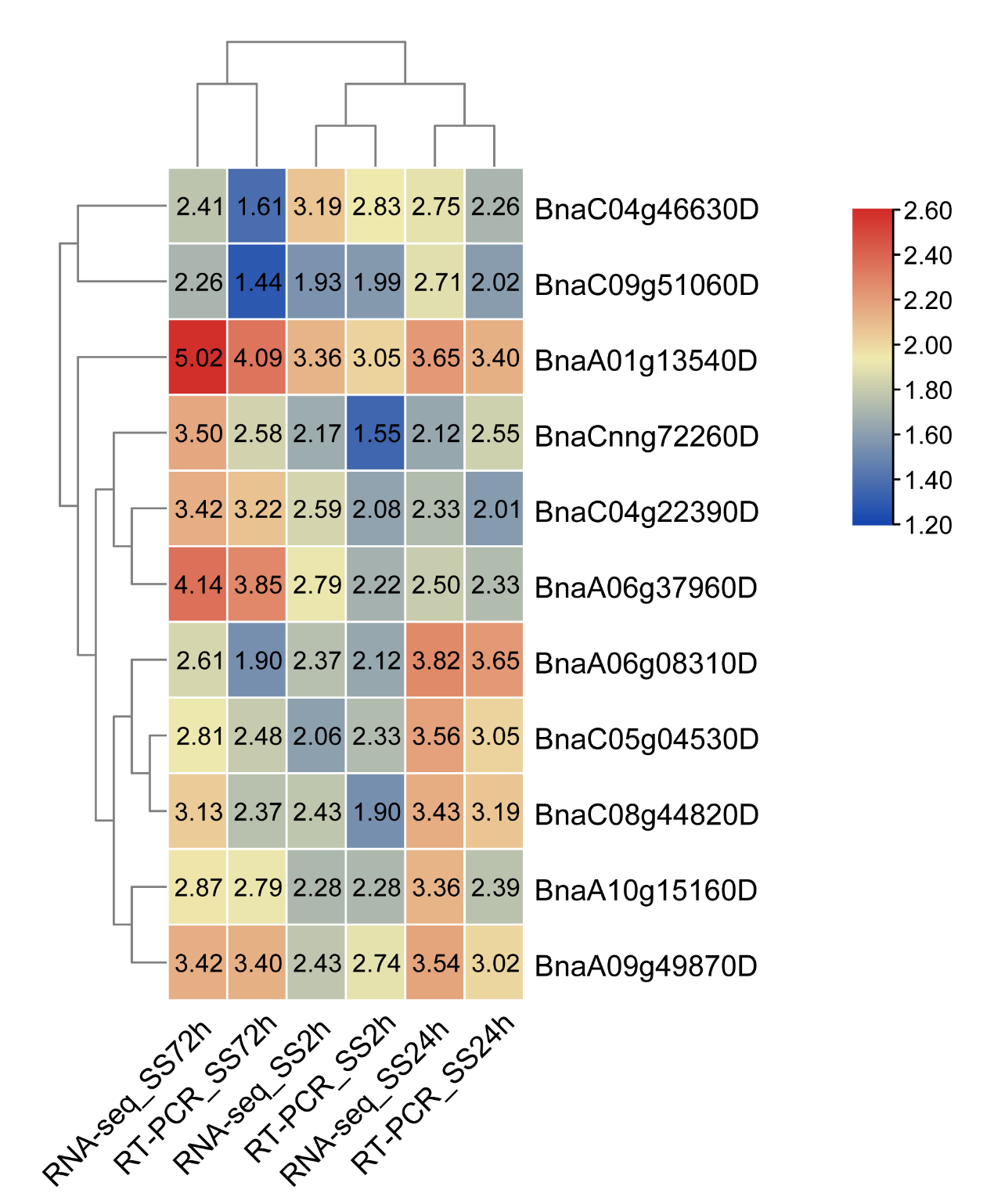


Fig. S2 qRT-PCR validation of transcript levels evaluated by RNA-Seq in canola roots under 200 mM NaCl stress.
