# Supplementary material for: Salt‑responsive transcriptome analysis of canola roots reveals candidate genes involved in the key metabolic pathway in response to salt stress
Source: Sci Rep. 2022 Jan 31;12:1666. doi: 10.1038/s41598-022-05700-2 (PMC8803978; doi:10.1038/s41598-022-05700-2)
Supplement: Supplementary file 2 — Supplementary Information 2. [file 41598_2022_5700_MOESM2_ESM.docx]

**Table S1** Statistics results of transcriptome sequencing in roots of canola under salt stress.

**Table S2** GO enrichment analysis of DEGs in roots of canola seedlings subjected to 2 h salt stress treatment compared to control (0 h).

**Table S3** GO enrichment analysis of DEGs in roots of canola seedlings subjected to 24 h salt stress treatment compared to control (0 h).

**Table S4** GO enrichment analysis of DEGs in roots of canola seedlings subjected to 72 h salt stress treatment compared to control (0 h).

**Table S5** KEGG pathway analysis of differentially expressed genes in roots of canola seedlings subjected to 2 h salt stress treatment compared to control (0 h).

**Table S6** KEGG pathway analysis of differentially expressed genes in roots of canola seedlings subjected to 24 h salt stress treatment compared to control (0 h).

**Table S7** KEGG pathway analysis of differentially expressed genes in roots of canola seedlings subjected to 72 h salt stress treatment compared to control (0 h).

**Table S8** Significant enrichment pathways of DEGs KEGG pathway analysis in roots of canola seedlings subjected to 2 h, 24 h, 72 h salt stress treatment compared to control (0 h).

**Table S9** Differential expression of transcription factors (TFs) in roots of canola seedlings under salt stress.

**Table S10** Primers used for qRT-PCR analysis of genes.

**Table S11** Genes involved in plant hormone signal transduction in canola roots under salt stress.

Table S1 Statistics results of transcriptome sequencing in roots of canola under salt stress.

| Samples | Total Reads | Clean reads | Clean bases | Mapped Reads | Uniq Mapped Reads | Multiple Map Reads | GC  Content (%) | Q30 (%) |
| --- | --- | --- | --- | --- | --- | --- | --- | --- |
| CK1 | 54,959,470 | 27,479,735 | 8,184,940,930 | 34,653,799 (63.05%) | 28,200,860 (51.31%) | 6,452,939 (11.74%) | 48.80% | 91.87% |
| CK2 | 50,111,156 | 25,055,578 | 7,453,488,152 | 38,500,421 (76.83%) | 31,232,143 (62.33%) | 7,268,278 (14.50%) | 47.57% | 91.64% |
| CK3 | 54,372,036 | 27,186,018 | 8,063,115,676 | 38,724,902 (71.22%) | 31,479,468 (57.90%) | 7,245,434 (13.33%) | 46.03% | 92.04% |
| SS-2h1 | 71,171,584 | 35,585,792 | 10,596,262,530 | 50,256,635 (70.61%) | 40,709,380 (57.20%) | 7,920,762 (13.60%) | 46.78% | 94.03% |
| SS-2h2 | 59,239,156 | 29,619,578 | 8,834,416,284 | 32,959,501 (55.64%) | 26,732,736 (45.13%) | 7,390,150 (14.19%) | 46.35% | 94.13% |
| SS-2h3 | 58,463,116 | 29,231,558 | 8,700,632,468 | 31,701,546 (54.22%) | 25,685,377 (43.93%) | 10,588,676 (15.58%) | 46.31% | 93.87% |
| SS-24h1 | 58,247,110 | 29,123,555 | 8,690,569,100 | 42,972,532 (73.78%) | 35,051,770 (60.18%) | 9,547,255 (13.41%) | 47.85% | 93.61% |
| SS-24h2 | 52,078,882 | 26,039,441 | 7,765,189,098 | 39,078,388 (75.04%) | 31,688,238 (60.85%) | 6,226,765 (10.51%) | 47.56% | 93.40% |
| SS-24h3 | 67,967,460 | 33,983,730 | 10,085,496,664 | 54,145,602 (79.66%) | 43,556,926 (64.08%) | 6,016,169 (10.29%) | 47.81% | 93.42% |
| SS-72h1 | 46,520,952 | 23,260,476 | 6,945,526,042 | 21,165,824 (45.50%) | 17,264,256 (37.11%) | 3,901,568 (8.39%) | 46.94% | 93.78% |
| SS-72h2 | 45,882,436 | 22,941,218 | 6,853,925,328 | 21,310,972 (46.45%) | 17,408,379 (37.94%) | 3,902,593 (8.51%) | 47.02% | 93.85% |
| SS-72h3 | 45,368,884 | 22,684,442 | 6,780,463,412 | 20,733,828 (45.70%) | 16,954,148 (37.37%) | 3,779,680 (8.33%) | 47.07% | 93.22% |
| Total | 664,382,242 | 332,191,121 | 98,954,025,684 | 426,203,950（63.14%） | 345,963,681（51.28%） | 80,240,269（11.87%） | 47.17% | 93.24% |

Table S2 GO enrichment analysis of DEGs in roots of canola seedlings subjected to 2 h salt stress treatment compared to control (0 h).

| ID | Description | GeneRatio | BgRatio | pvalue | p.adjust | qvalue | Count | GO_type |
| --- | --- | --- | --- | --- | --- | --- | --- | --- |
| GO:0006979 | response to oxidative stress | 57/3269 | 340/54264 | 2.57E-12 | 1.34E-09 | 1.18E-09 | 57 | biological process |
| GO:0006073 | cellular glucan metabolic process | 22/3269 | 95/54264 | 3.81E-08 | 2.49E-06 | 2.18E-06 | 22 | biological process |
| GO:0007585 | respiratory gaseous exchange by respiratory system | 8/3269 | 13/54264 | 1.68E-07 | 8.81E-06 | 7.71E-06 | 8 | biological process |
| GO:0006855 | drug transmembrane transport | 35/3269 | 237/54264 | 9.12E-07 | 3.18E-05 | 2.78E-05 | 35 | biological process |
| GO:0009664 | plant-type cell wall organization | 18/3269 | 108/54264 | 7.89E-05 | 0.001966 | 0.001721 | 18 | biological process |
| GO:0030244 | cellulose biosynthetic process | 17/3269 | 100/54264 | 9.58E-05 | 0.002276 | 0.001993 | 17 | biological process |
| GO:0005985 | sucrose metabolic process | 8/3269 | 27/54264 | 0.000136 | 0.00307 | 0.002688 | 8 | biological process |
| GO:0043086 | negative regulation of catalytic activity | 20/3269 | 134/54264 | 0.000158 | 0.00307 | 0.002688 | 20 | biological process |
| GO:0000103 | sulfate assimilation | 7/3269 | 26/54264 | 0.00068 | 0.011117 | 0.009733 | 7 | biological process |
| GO:0006536 | glutamate metabolic process | 7/3269 | 26/54264 | 0.00068 | 0.011117 | 0.009733 | 7 | biological process |
| GO:0006002 | fructose 6-phosphate metabolic process | 7/3269 | 27/54264 | 0.000871 | 0.013215 | 0.01157 | 7 | biological process |
| GO:0006817 | phosphate ion transport | 6/3269 | 20/54264 | 0.000884 | 0.013215 | 0.01157 | 6 | biological process |
| GO:0008272 | sulfate transport | 10/3269 | 52/54264 | 0.000954 | 0.013485 | 0.011807 | 10 | biological process |
| GO:0019538 | protein metabolic process | 10/3269 | 55/54264 | 0.001497 | 0.01909 | 0.016714 | 10 | biological process |
| GO:0009690 | cytokinin metabolic process | 6/3269 | 23/54264 | 0.001971 | 0.023972 | 0.020988 | 6 | biological process |
| GO:0009408 | response to heat | 6/3269 | 25/54264 | 0.003118 | 0.034118 | 0.029871 | 6 | biological process |
| GO:0016998 | cell wall macromolecule catabolic process | 13/3269 | 91/54264 | 0.003133 | 0.034118 | 0.029871 | 13 | biological process |
| GO:0006950 | response to stress | 26/3269 | 242/54264 | 0.003199 | 0.034118 | 0.029871 | 26 | biological process |
| GO:0005618 | cell wall | 44/3269 | 330/54264 | 7.35E-07 | 3.18E-05 | 2.78E-05 | 44 | cellular component |
| GO:0048046 | apoplast | 32/3269 | 205/54264 | 7.64E-07 | 3.18E-05 | 2.78E-05 | 32 | cellular component |
| GO:0005576 | extracellular region | 24/3269 | 221/54264 | 0.003921 | 0.040212 | 0.035206 | 24 | cellular component |
| GO:0004601 | peroxidase activity | 52/3269 | 327/54264 | 1.67E-10 | 4.37E-08 | 3.82E-08 | 52 | molecular function |
| GO:0045735 | nutrient reservoir activity | 28/3269 | 119/54264 | 3.80E-10 | 6.62E-08 | 5.80E-08 | 28 | molecular function |
| GO:0004190 | aspartic-type endopeptidase activity | 48/3269 | 305/54264 | 1.18E-09 | 1.54E-07 | 1.35E-07 | 48 | molecular function |
| GO:0030246 | carbohydrate binding | 58/3269 | 422/54264 | 4.62E-09 | 4.84E-07 | 4.24E-07 | 58 | molecular function |
| GO:0008171 | O-methyltransferase activity | 24/3269 | 110/54264 | 3.21E-08 | 2.49E-06 | 2.18E-06 | 24 | molecular function |
| GO:0016762 | xyloglucan:xyloglucosyl transferase activity | 22/3269 | 95/54264 | 3.81E-08 | 2.49E-06 | 2.18E-06 | 22 | molecular function |
| GO:0009916 | alternative oxidase activity | 8/3269 | 13/54264 | 1.68E-07 | 8.81E-06 | 7.71E-06 | 8 | molecular function |
| GO:0015297 | antiporter activity | 35/3269 | 237/54264 | 9.12E-07 | 3.18E-05 | 2.78E-05 | 35 | molecular function |
| GO:0022857 | transmembrane transporter activity | 39/3269 | 295/54264 | 3.74E-06 | 0.000122 | 0.000107 | 39 | molecular function |
| GO:0016760 | cellulose synthase (UDP-forming) activity | 16/3269 | 77/54264 | 1.18E-05 | 0.000362 | 0.000317 | 16 | molecular function |
| GO:0004020 | adenylylsulfate kinase activity | 7/3269 | 16/54264 | 2.02E-05 | 0.000586 | 0.000513 | 7 | molecular function |
| GO:0004435 | phosphatidylinositol phospholipase C activity | 10/3269 | 36/54264 | 3.70E-05 | 0.001019 | 0.000892 | 10 | molecular function |
| GO:0051087 | chaperone binding | 10/3269 | 38/54264 | 6.16E-05 | 0.001612 | 0.001411 | 10 | molecular function |
| GO:0004350 | glutamate-5-semialdehyde dehydrogenase activity | 5/3269 | 10/54264 | 0.000154 | 0.00307 | 0.002688 | 5 | molecular function |
| GO:0046524 | sucrose-phosphate synthase activity | 5/3269 | 10/54264 | 0.000154 | 0.00307 | 0.002688 | 5 | molecular function |
| GO:0042802 | identical protein binding | 20/3269 | 134/54264 | 0.000158 | 0.00307 | 0.002688 | 20 | molecular function |
| GO:0008113 | peptide-methionine (S)-S-oxide reductase activity | 7/3269 | 23/54264 | 0.000297 | 0.005554 | 0.004862 | 7 | molecular function |
| GO:0004351 | glutamate decarboxylase activity | 7/3269 | 26/54264 | 0.00068 | 0.011117 | 0.009733 | 7 | molecular function |
| GO:0005315 | inorganic phosphate transmembrane transporter activity | 6/3269 | 20/54264 | 0.000884 | 0.013215 | 0.01157 | 6 | molecular function |
| GO:0015116 | sulfate transmembrane transporter activity | 10/3269 | 52/54264 | 0.000954 | 0.013485 | 0.011807 | 10 | molecular function |
| GO:0004349 | glutamate 5-kinase activity | 5/3269 | 14/54264 | 0.001 | 0.013762 | 0.012049 | 5 | molecular function |
| GO:0008271 | secondary active sulfate transmembrane transporter activity | 9/3269 | 44/54264 | 0.00107 | 0.014347 | 0.012561 | 9 | molecular function |
| GO:0016165 | linoleate 13S-lipoxygenase activity | 7/3269 | 28/54264 | 0.001101 | 0.014398 | 0.012606 | 7 | molecular function |
| GO:0019139 | cytokinin dehydrogenase activity | 6/3269 | 23/54264 | 0.001971 | 0.023972 | 0.020988 | 6 | molecular function |
| GO:0008146 | sulfotransferase activity | 16/3269 | 119/54264 | 0.002126 | 0.025273 | 0.022127 | 16 | molecular function |
| GO:0050660 | flavin adenine dinucleotide binding | 39/3269 | 406/54264 | 0.002935 | 0.034113 | 0.029867 | 39 | molecular function |
| GO:0008061 | chitin binding | 8/3269 | 42/54264 | 0.003217 | 0.034118 | 0.029871 | 8 | molecular function |
| GO:0004806 | triglyceride lipase activity | 15/3269 | 113/54264 | 0.003262 | 0.034118 | 0.029871 | 15 | molecular function |

Table S3 GO enrichment analysis of DEGs in roots of canola seedlings subjected to 24 h salt stress treatment compared to control (0 h).

| ID | Description | GeneRatio | BgRatio | pvalue | p.adjust | qvalue | Count | GO_type |
| --- | --- | --- | --- | --- | --- | --- | --- | --- |
| GO:0006950 | response to stress | 32/2769 | 242/54264 | 8.92E-07 | 0.000108 | 9.20E-05 | 32 | biological process |
| GO:0004190 | aspartic-type endopeptidase activity | 37/2769 | 305/54264 | 1.12E-06 | 0.000108 | 9.20E-05 | 37 | molecular function |
| GO:0007585 | respiratory gaseous exchange by respiratory system | 7/2769 | 13/54264 | 1.17E-06 | 0.000108 | 9.20E-05 | 7 | biological process |
| GO:0009916 | alternative oxidase activity | 7/2769 | 13/54264 | 1.17E-06 | 0.000108 | 9.20E-05 | 7 | molecular function |
| GO:0006073 | cellular glucan metabolic process | 18/2769 | 95/54264 | 1.34E-06 | 0.000108 | 9.20E-05 | 18 | biological process |
| GO:0016762 | xyloglucan:xyloglucosyl transferase activity | 18/2769 | 95/54264 | 1.34E-06 | 0.000108 | 9.20E-05 | 18 | molecular function |
| GO:0006855 | drug transmembrane transport | 30/2769 | 237/54264 | 4.73E-06 | 0.000255 | 0.000217 | 30 | biological process |
| GO:0015297 | antiporter activity | 30/2769 | 237/54264 | 4.73E-06 | 0.000255 | 0.000217 | 30 | molecular function |
| GO:0048046 | apoplast | 27/2769 | 205/54264 | 6.60E-06 | 0.000321 | 0.000272 | 27 | cellular component |
| GO:0008762 | UDP-N-acetylmuramate dehydrogenase activity | 19/2769 | 119/54264 | 9.65E-06 | 0.000426 | 0.000362 | 19 | molecular function |
| GO:0003777 | microtubule motor activity | 24/2769 | 176/54264 | 1.16E-05 | 0.000435 | 0.000369 | 24 | molecular function |
| GO:0007018 | microtubule-based movement | 24/2769 | 176/54264 | 1.16E-05 | 0.000435 | 0.000369 | 24 | biological process |
| GO:0005875 | microtubule associated complex | 25/2769 | 198/54264 | 2.94E-05 | 0.001021 | 0.000867 | 25 | cellular component |
| GO:0006002 | fructose 6-phosphate metabolic process | 8/2769 | 27/54264 | 4.22E-05 | 0.001366 | 0.00116 | 8 | biological process |
| GO:0004350 | glutamate-5-semialdehyde dehydrogenase activity | 5/2769 | 10/54264 | 7.00E-05 | 0.00189 | 0.001604 | 5 | molecular function |
| GO:0004564 | beta-fructofuranosidase activity | 5/2769 | 10/54264 | 7.00E-05 | 0.00189 | 0.001604 | 5 | molecular function |
| GO:0004575 | sucrose alpha-glucosidase activity | 5/2769 | 10/54264 | 7.00E-05 | 0.00189 | 0.001604 | 5 | molecular function |
| GO:0019538 | protein metabolic process | 11/2769 | 55/54264 | 8.93E-05 | 0.002192 | 0.001861 | 11 | biological process |
| GO:0004020 | adenylylsulfate kinase activity | 6/2769 | 16/54264 | 9.02E-05 | 0.002192 | 0.001861 | 6 | molecular function |
| GO:0005819 | spindle | 5/2769 | 12/54264 | 0.000202 | 0.00467 | 0.003965 | 5 | cellular component |
| GO:0008061 | chitin binding | 9/2769 | 42/54264 | 0.000223 | 0.004924 | 0.00418 | 9 | molecular function |
| GO:0006741 | NADP biosynthetic process | 5/2769 | 13/54264 | 0.000314 | 0.006361 | 0.005401 | 5 | biological process |
| GO:0019674 | NAD metabolic process | 5/2769 | 13/54264 | 0.000314 | 0.006361 | 0.005401 | 5 | biological process |
| GO:0003872 | 6-phosphofructokinase activity | 8/2769 | 36/54264 | 0.000381 | 0.00711 | 0.006036 | 8 | molecular function |
| GO:0005945 | 6-phosphofructokinase complex | 8/2769 | 36/54264 | 0.000381 | 0.00711 | 0.006036 | 8 | cellular component |
| GO:0004568 | chitinase activity | 11/2769 | 65/54264 | 0.000418 | 0.00711 | 0.006036 | 11 | molecular function |
| GO:0006032 | chitin catabolic process | 11/2769 | 65/54264 | 0.000418 | 0.00711 | 0.006036 | 11 | biological process |
| GO:0003951 | NAD+ kinase activity | 5/2769 | 14/54264 | 0.000468 | 0.00711 | 0.006036 | 5 | molecular function |
| GO:0004349 | glutamate 5-kinase activity | 5/2769 | 14/54264 | 0.000468 | 0.00711 | 0.006036 | 5 | molecular function |
| GO:0006790 | sulfur compound metabolic process | 5/2769 | 14/54264 | 0.000468 | 0.00711 | 0.006036 | 5 | biological process |
| GO:0009072 | aromatic amino acid family metabolic process | 5/2769 | 14/54264 | 0.000468 | 0.00711 | 0.006036 | 5 | biological process |
| GO:0051726 | regulation of cell cycle | 12/2769 | 79/54264 | 0.000646 | 0.009516 | 0.00808 | 12 | biological process |
| GO:0005576 | extracellular region | 23/2769 | 221/54264 | 0.001005 | 0.014372 | 0.012202 | 23 | cellular component |
| GO:0008146 | sulfotransferase activity | 15/2769 | 119/54264 | 0.001101 | 0.014556 | 0.012359 | 15 | molecular function |
| GO:0008569 | ATP-dependent microtubule motor activity, minus-end-directed | 4/2769 | 10/54264 | 0.001108 | 0.014556 | 0.012359 | 4 | molecular function |
| GO:0010277 | chlorophyllide a oxygenase [overall] activity | 4/2769 | 10/54264 | 0.001108 | 0.014556 | 0.012359 | 4 | molecular function |
| GO:0009408 | response to heat | 6/2769 | 25/54264 | 0.001342 | 0.016729 | 0.014204 | 6 | biological process |
| GO:0006979 | response to oxidative stress | 31/2769 | 340/54264 | 0.001413 | 0.01717 | 0.014578 | 31 | biological process |
| GO:0004089 | carbonate dehydratase activity | 8/2769 | 44/54264 | 0.001554 | 0.018415 | 0.015635 | 8 | molecular function |
| GO:0000103 | sulfate assimilation | 6/2769 | 26/54264 | 0.001671 | 0.01933 | 0.016412 | 6 | biological process |
| GO:0030244 | cellulose biosynthetic process | 13/2769 | 100/54264 | 0.001734 | 0.019598 | 0.016639 | 13 | biological process |
| GO:0016760 | cellulose synthase (UDP-forming) activity | 11/2769 | 77/54264 | 0.001786 | 0.019732 | 0.016753 | 11 | molecular function |
| GO:0030246 | carbohydrate binding | 36/2769 | 422/54264 | 0.001984 | 0.02126 | 0.01805 | 36 | molecular function |
| GO:0004435 | phosphatidylinositol phospholipase C activity | 7/2769 | 36/54264 | 0.002021 | 0.02126 | 0.01805 | 7 | molecular function |
| GO:0015976 | carbon utilization | 6/2769 | 27/54264 | 0.002056 | 0.02126 | 0.01805 | 6 | biological process |
| GO:0016998 | cell wall macromolecule catabolic process | 12/2769 | 91/54264 | 0.002271 | 0.022991 | 0.01952 | 12 | biological process |
| GO:0005992 | trehalose biosynthetic process | 9/2769 | 58/54264 | 0.002558 | 0.025369 | 0.021539 | 9 | biological process |
| GO:0022857 | transmembrane transporter activity | 27/2769 | 295/54264 | 0.002612 | 0.02539 | 0.021557 | 27 | molecular function |
| GO:0045735 | nutrient reservoir activity | 14/2769 | 119/54264 | 0.003044 | 0.02901 | 0.024631 | 14 | molecular function |
| GO:0000079 | regulation of cyclin-dependent protein serine/threonine kinase activity | 14/2769 | 122/54264 | 0.003823 | 0.035057 | 0.029764 | 14 | biological process |
| GO:0019901 | protein kinase binding | 14/2769 | 122/54264 | 0.003823 | 0.035057 | 0.029764 | 14 | molecular function |
| GO:0016844 | strictosidine synthase activity | 8/2769 | 51/54264 | 0.004072 | 0.036647 | 0.031114 | 8 | molecular function |
| GO:0004478 | methionine adenosyltransferase activity | 4/2769 | 14/54264 | 0.004483 | 0.037863 | 0.032147 | 4 | molecular function |
| GO:0006556 | S-adenosylmethionine biosynthetic process | 4/2769 | 14/54264 | 0.004483 | 0.037863 | 0.032147 | 4 | biological process |
| GO:0016485 | protein processing | 4/2769 | 14/54264 | 0.004483 | 0.037863 | 0.032147 | 4 | biological process |
| GO:0009058 | biosynthetic process | 38/2769 | 475/54264 | 0.004519 | 0.037863 | 0.032147 | 38 | biological process |
| GO:0004601 | peroxidase activity | 28/2769 | 327/54264 | 0.005557 | 0.045771 | 0.038861 | 28 | molecular function |
| GO:0051082 | unfolded protein binding | 24/2769 | 268/54264 | 0.005693 | 0.04611 | 0.039149 | 24 | molecular function |
| GO:0050660 | flavin adenine dinucleotide binding | 33/2769 | 406/54264 | 0.006135 | 0.048877 | 0.041498 | 33 | molecular function |
| GO:0005618 | cell wall | 28/2769 | 330/54264 | 0.006272 | 0.049166 | 0.041744 | 28 | cellular component |

Table S4 GO enrichment analysis of DEGs in roots of canola seedlings subjected to 72 h salt stress treatment compared to control (0 h).

| ID | Description | GeneRatio | BgRatio | pvalue | p.adjust | qvalue | Count | GO_type |
| --- | --- | --- | --- | --- | --- | --- | --- | --- |
| GO:0022857 | transmembrane transporter activity | 63/4339 | 295/54264 | 5.77E-13 | 2.61E-10 | 2.31E-10 | 63 | molecular function |
| GO:0008061 | chitin binding | 21/4339 | 42/54264 | 8.96E-13 | 2.61E-10 | 2.31E-10 | 21 | molecular function |
| GO:0004601 | peroxidase activity | 60/4339 | 327/54264 | 1.25E-09 | 2.43E-07 | 2.14E-07 | 60 | molecular function |
| GO:0004568 | chitinase activity | 22/4339 | 65/54264 | 2.84E-09 | 3.31E-07 | 2.92E-07 | 22 | molecular function |
| GO:0006032 | chitin catabolic process | 22/4339 | 65/54264 | 2.84E-09 | 3.31E-07 | 2.92E-07 | 22 | biological process |
| GO:0006979 | response to oxidative stress | 60/4339 | 340/54264 | 5.76E-09 | 5.60E-07 | 4.94E-07 | 60 | biological process |
| GO:0006855 | drug transmembrane transport | 46/4339 | 237/54264 | 1.71E-08 | 1.11E-06 | 9.77E-07 | 46 | biological process |
| GO:0015297 | antiporter activity | 46/4339 | 237/54264 | 1.71E-08 | 1.11E-06 | 9.77E-07 | 46 | molecular function |
| GO:0016760 | cellulose synthase (UDP-forming) activity | 22/4339 | 77/54264 | 9.26E-08 | 4.91E-06 | 4.33E-06 | 22 | molecular function |
| GO:0008171 | O-methyltransferase activity | 27/4339 | 110/54264 | 1.12E-07 | 5.46E-06 | 4.82E-06 | 27 | molecular function |
| GO:0016998 | cell wall macromolecule catabolic process | 24/4339 | 91/54264 | 1.31E-07 | 5.90E-06 | 5.21E-06 | 24 | biological process |
| GO:0006073 | cellular glucan metabolic process | 23/4339 | 95/54264 | 1.23E-06 | 4.79E-05 | 4.23E-05 | 23 | biological process |
| GO:0016762 | xyloglucan:xyloglucosyl transferase activity | 23/4339 | 95/54264 | 1.23E-06 | 4.79E-05 | 4.23E-05 | 23 | molecular function |
| GO:0007585 | respiratory gaseous exchange by respiratory system | 8/4339 | 13/54264 | 1.48E-06 | 5.08E-05 | 4.48E-05 | 8 | biological process |
| GO:0009916 | alternative oxidase activity | 8/4339 | 13/54264 | 1.48E-06 | 5.08E-05 | 4.48E-05 | 8 | molecular function |
| GO:0008146 | sulfotransferase activity | 26/4339 | 119/54264 | 2.10E-06 | 6.81E-05 | 6.02E-05 | 26 | molecular function |
| GO:0016887 | ATPase activity | 58/4339 | 395/54264 | 5.50E-06 | 0.000169 | 0.000149 | 58 | molecular function |
| GO:0030244 | cellulose biosynthetic process | 22/4339 | 100/54264 | 1.10E-05 | 0.000322 | 0.000284 | 22 | biological process |
| GO:0000976 | transcription regulatory region sequence-specific DNA binding | 17/4339 | 69/54264 | 2.28E-05 | 0.000633 | 0.000559 | 17 | molecular function |
| GO:0004190 | aspartic-type endopeptidase activity | 46/4339 | 305/54264 | 2.47E-05 | 0.000653 | 0.000577 | 46 | molecular function |
| GO:0005618 | cell wall | 48/4339 | 330/54264 | 4.28E-05 | 0.001085 | 0.000958 | 48 | cellular component |
| GO:0030246 | carbohydrate binding | 57/4339 | 422/54264 | 7.42E-05 | 0.001802 | 0.001591 | 57 | molecular function |
| GO:0048046 | apoplast | 33/4339 | 205/54264 | 9.22E-05 | 0.002149 | 0.001898 | 33 | cellular component |
| GO:0008271 | secondary active sulfate transmembrane transporter activity | 12/4339 | 44/54264 | 0.000125 | 0.002813 | 0.002483 | 12 | molecular function |
| GO:0004013 | adenosylhomocysteinase activity | 6/4339 | 12/54264 | 0.000158 | 0.003403 | 0.003004 | 6 | molecular function |
| GO:0008272 | sulfate transport | 13/4339 | 52/54264 | 0.000174 | 0.003505 | 0.003095 | 13 | biological process |
| GO:0015116 | sulfate transmembrane transporter activity | 13/4339 | 52/54264 | 0.000174 | 0.003505 | 0.003095 | 13 | molecular function |
| GO:0016165 | linoleate 13S-lipoxygenase activity | 9/4339 | 28/54264 | 0.000225 | 0.004366 | 0.003855 | 9 | molecular function |
| GO:0003950 | NAD+ ADP-ribosyltransferase activity | 9/4339 | 29/54264 | 0.000303 | 0.005693 | 0.005027 | 9 | molecular function |
| GO:0000151 | ubiquitin ligase complex | 30/4339 | 200/54264 | 0.000638 | 0.011631 | 0.010269 | 30 | cellular component |
| GO:0004351 | glutamate decarboxylase activity | 8/4339 | 26/54264 | 0.000698 | 0.011971 | 0.010569 | 8 | molecular function |
| GO:0006536 | glutamate metabolic process | 8/4339 | 26/54264 | 0.000698 | 0.011971 | 0.010569 | 8 | biological process |
| GO:0009664 | plant-type cell wall organization | 19/4339 | 108/54264 | 0.000893 | 0.014874 | 0.013133 | 19 | biological process |
| GO:0004020 | adenylylsulfate kinase activity | 6/4339 | 16/54264 | 0.001032 | 0.016714 | 0.014757 | 6 | molecular function |
| GO:0004806 | triglyceride lipase activity | 19/4339 | 113/54264 | 0.001557 | 0.02453 | 0.021658 | 19 | molecular function |
| GO:0008113 | peptide-methionine (S)-S-oxide reductase activity | 7/4339 | 23/54264 | 0.001619 | 0.024842 | 0.021933 | 7 | molecular function |
| GO:0004084 | branched-chain-amino-acid transaminase activity | 8/4339 | 31/54264 | 0.00246 | 0.03581 | 0.031617 | 8 | molecular function |
| GO:0009081 | branched-chain amino acid metabolic process | 8/4339 | 31/54264 | 0.00246 | 0.03581 | 0.031617 | 8 | biological process |
| GO:0006071 | glycerol metabolic process | 12/4339 | 60/54264 | 0.002518 | 0.03581 | 0.031617 | 12 | biological process |
| GO:0005199 | structural constituent of cell wall | 6/4339 | 19/54264 | 0.002838 | 0.039394 | 0.034781 | 6 | molecular function |
| GO:0006950 | response to stress | 32/4339 | 242/54264 | 0.003504 | 0.046557 | 0.041106 | 32 | biological process |
| GO:0003978 | UDP-glucose 4-epimerase activity | 7/4339 | 26/54264 | 0.003514 | 0.046557 | 0.041106 | 7 | molecular function |

Table S5 KEGG pathway analysis of differentially expressed genes in roots of canola seedlings subjected to 2 h salt stress treatment compared to control (0 h).

| #Term | ID | Input number | Background number | Enrich ratio | P-Value | Corrected P-Value |
| --- | --- | --- | --- | --- | --- | --- |
| Metabolic pathways | bna01100 | 566 | 7939 | 0.071294 | 2.65E-18 | 3.34E-16 |
| Phenylpropanoid biosynthesis | bna00940 | 89 | 632 | 0.140823 | 1.09E-16 | 6.85E-15 |
| Plant hormone signal transduction | bna04075 | 117 | 1187 | 0.098568 | 1.70E-11 | 7.15E-10 |
| Protein processing in endoplasmic reticulum | bna04141 | 81 | 707 | 0.114569 | 3.80E-11 | 1.20E-09 |
| Biosynthesis of secondary metabolites | bna01110 | 287 | 3914 | 0.073327 | 6.70E-11 | 1.69E-09 |
| Zeatin biosynthesis | bna00908 | 23 | 107 | 0.214953 | 3.42E-08 | 7.19E-07 |
| Galactose metabolism | bna00052 | 26 | 176 | 0.147727 | 2.90E-06 | 5.22E-05 |
| Starch and sucrose metabolism | bna00500 | 51 | 549 | 0.092896 | 3.20E-05 | 0.000504 |
| Plant-pathogen interaction | bna04626 | 56 | 643 | 0.087092 | 6.90E-05 | 0.000966 |
| Nitrogen metabolism | bna00910 | 21 | 156 | 0.134615 | 8.32E-05 | 0.001049 |
| Pentose and glucuronate interconversions | bna00040 | 42 | 443 | 0.094808 | 0.0001 | 0.001148 |
| Spliceosome | bna03040 | 53 | 619 | 0.085622 | 0.000156 | 0.001639 |
| Taurine and hypotaurine metabolism | bna00430 | 12 | 63 | 0.190476 | 0.00017 | 0.001651 |
| Sulfur metabolism | bna00920 | 20 | 154 | 0.12987 | 0.000187 | 0.001682 |
| AGE-RAGE signaling pathway in diabetic complications | bna04933 | 14 | 87 | 0.16092 | 0.000244 | 0.002019 |
| Cysteine and methionine metabolism | bna00270 | 40 | 435 | 0.091954 | 0.000256 | 0.002019 |
| Alanine, aspartate and glutamate metabolism | bna00250 | 23 | 200 | 0.115 | 0.000324 | 0.002402 |
| alpha-Linolenic acid metabolism | bna00592 | 20 | 164 | 0.121951 | 0.000392 | 0.002745 |
| Linoleic acid metabolism | bna00591 | 9 | 48 | 0.1875 | 0.001192 | 0.007906 |
| Arginine and proline metabolism | bna00330 | 21 | 202 | 0.10396 | 0.001822 | 0.011477 |
| Endocytosis | bna04144 | 44 | 593 | 0.074199 | 0.006434 | 0.038602 |
| Glycerolipid metabolism | bna00561 | 25 | 293 | 0.085324 | 0.007831 | 0.044848 |
| Stilbenoid, diarylheptanoid and gingerol biosynthesis | bna00945 | 8 | 55 | 0.145455 | 0.008576 | 0.046983 |
| Inositol phosphate metabolism | bna00562 | 23 | 266 | 0.086466 | 0.009073 | 0.047631 |
| Riboflavin metabolism | bna00740 | 8 | 59 | 0.135593 | 0.012281 | 0.061896 |
| Ubiquinone and other terpenoid-quinone biosynthesis | bna00130 | 14 | 142 | 0.098592 | 0.014364 | 0.063582 |
| Amino sugar and nucleotide sugar metabolism | bna00520 | 34 | 457 | 0.074398 | 0.014499 | 0.063582 |
| Cutin, suberine and wax biosynthesis | bna00073 | 12 | 114 | 0.105263 | 0.014602 | 0.063582 |
| MAPK signaling pathway - plant | bna04016 | 40 | 558 | 0.071685 | 0.014634 | 0.063582 |
| Glucosinolate biosynthesis | bna00966 | 7 | 50 | 0.14 | 0.016107 | 0.067648 |
| Circadian rhythm - plant | bna04712 | 13 | 131 | 0.099237 | 0.017095 | 0.069482 |
| Flavonoid biosynthesis | bna00941 | 11 | 105 | 0.104762 | 0.019413 | 0.076439 |
| Vitamin B6 metabolism | bna00750 | 7 | 53 | 0.132075 | 0.020885 | 0.079743 |
| Cyanoamino acid metabolism | bna00460 | 19 | 232 | 0.081897 | 0.026195 | 0.097077 |
| Phosphatidylinositol signaling system | bna04070 | 21 | 275 | 0.076364 | 0.037261 | 0.134139 |
| Glycolysis / Gluconeogenesis | bna00010 | 30 | 429 | 0.06993 | 0.039832 | 0.139413 |
| Sesquiterpenoid and triterpenoid biosynthesis | bna00909 | 6 | 50 | 0.12 | 0.044948 | 0.153065 |
| ABC transporters | bna02010 | 8 | 82 | 0.097561 | 0.058083 | 0.192591 |
| Biosynthesis of secondary metabolites - other antibiotics | bna00998 | 2 | 7 | 0.285714 | 0.062511 | 0.20196 |
| Biosynthesis of secondary metabolites - unclassified | bna00999 | 6 | 57 | 0.105263 | 0.071943 | 0.226622 |
| Butanoate metabolism | bna00650 | 7 | 75 | 0.093333 | 0.086814 | 0.266794 |
| Tyrosine metabolism | bna00350 | 11 | 139 | 0.079137 | 0.091149 | 0.273448 |
| Pyruvate metabolism | bna00620 | 20 | 294 | 0.068027 | 0.097617 | 0.286041 |
| Phenylalanine metabolism | bna00360 | 9 | 117 | 0.076923 | 0.132897 | 0.380568 |
| Brassinosteroid biosynthesis | bna00905 | 4 | 40 | 0.1 | 0.146642 | 0.410597 |
| Glutathione metabolism | bna00480 | 22 | 360 | 0.061111 | 0.178543 | 0.489054 |
| Valine, leucine and isoleucine degradation | bna00280 | 11 | 162 | 0.067901 | 0.182993 | 0.490576 |
| Fructose and mannose metabolism | bna00051 | 14 | 225 | 0.062222 | 0.22394 | 0.577054 |
| Carbon fixation in photosynthetic organisms | bna00710 | 16 | 262 | 0.061069 | 0.22441 | 0.577054 |
| Glycosphingolipid biosynthesis - lacto and neolacto series | bna00601 | 2 | 18 | 0.111111 | 0.237522 | 0.594287 |
| Valine, leucine and isoleucine biosynthesis | bna00290 | 6 | 84 | 0.071429 | 0.240545 | 0.594287 |
| Arachidonic acid metabolism | bna00590 | 5 | 69 | 0.072464 | 0.260318 | 0.630376 |
| Fatty acid elongation | bna00062 | 7 | 105 | 0.066667 | 0.265158 | 0.630376 |
| Tryptophan metabolism | bna00380 | 12 | 200 | 0.06 | 0.283024 | 0.650832 |
| Sulfur relay system | bna04122 | 3 | 37 | 0.081081 | 0.284093 | 0.650832 |
| Folate biosynthesis | bna00790 | 5 | 76 | 0.065789 | 0.323877 | 0.723378 |
| Porphyrin and chlorophyll metabolism | bna00860 | 10 | 170 | 0.058824 | 0.327242 | 0.723378 |
| beta-Alanine metabolism | bna00410 | 10 | 172 | 0.05814 | 0.339525 | 0.737588 |
| Glycosaminoglycan degradation | bna00531 | 2 | 25 | 0.08 | 0.359101 | 0.766895 |
| Glycerophospholipid metabolism | bna00564 | 20 | 380 | 0.052632 | 0.400583 | 0.841224 |
| 2-Oxocarboxylic acid metabolism | bna01210 | 14 | 266 | 0.052632 | 0.427955 | 0.883974 |
| Monoterpenoid biosynthesis | bna00902 | 2 | 30 | 0.066667 | 0.441638 | 0.897522 |
| Steroid biosynthesis | bna00100 | 7 | 136 | 0.051471 | 0.498451 | 0.943888 |
| Base excision repair | bna03410 | 6 | 116 | 0.051724 | 0.502415 | 0.943888 |
| Biosynthesis of unsaturated fatty acids | bna01040 | 4 | 76 | 0.052632 | 0.512967 | 0.943888 |
| Glycosphingolipid biosynthesis - globo and isoglobo series | bna00603 | 2 | 35 | 0.057143 | 0.517958 | 0.943888 |
| Isoquinoline alkaloid biosynthesis | bna00950 | 4 | 77 | 0.051948 | 0.52279 | 0.943888 |
| Tropane, piperidine and pyridine alkaloid biosynthesis | bna00960 | 6 | 119 | 0.05042 | 0.526205 | 0.943888 |
| Non-homologous end-joining | bna03450 | 1 | 15 | 0.066667 | 0.532919 | 0.943888 |
| Photosynthesis | bna00195 | 12 | 245 | 0.04898 | 0.534281 | 0.943888 |
| Carotenoid biosynthesis | bna00906 | 5 | 100 | 0.05 | 0.541751 | 0.943888 |
| Biosynthesis of amino acids | bna01230 | 43 | 893 | 0.048152 | 0.552196 | 0.943888 |
| Propanoate metabolism | bna00640 | 6 | 123 | 0.04878 | 0.557173 | 0.943888 |
| Phosphonate and phosphinate metabolism | bna00440 | 2 | 38 | 0.052632 | 0.560288 | 0.943888 |
| Ascorbate and aldarate metabolism | bna00053 | 9 | 187 | 0.048128 | 0.561838 | 0.943888 |
| Basal transcription factors | bna03022 | 8 | 171 | 0.046784 | 0.594617 | 0.983678 |
| Pantothenate and CoA biosynthesis | bna00770 | 5 | 108 | 0.046296 | 0.607299 | 0.983678 |
| Arginine biosynthesis | bna00220 | 6 | 130 | 0.046154 | 0.608943 | 0.983678 |
| Nicotinate and nicotinamide metabolism | bna00760 | 3 | 65 | 0.046154 | 0.617471 | 0.984827 |
| Thiamine metabolism | bna00730 | 3 | 69 | 0.043478 | 0.655967 | 1 |
| Diterpenoid biosynthesis | bna00904 | 4 | 93 | 0.043011 | 0.664986 | 1 |
| Peroxisome | bna04146 | 14 | 318 | 0.044025 | 0.681753 | 1 |
| Pentose phosphate pathway | bna00030 | 8 | 187 | 0.042781 | 0.688954 | 1 |
| Purine metabolism | bna00230 | 14 | 324 | 0.04321 | 0.70661 | 1 |
| Other glycan degradation | bna00511 | 2 | 51 | 0.039216 | 0.712257 | 1 |
| Photosynthesis - antenna proteins | bna00196 | 3 | 78 | 0.038462 | 0.732113 | 1 |
| Fatty acid metabolism | bna01212 | 10 | 245 | 0.040816 | 0.750477 | 1 |
| Glycine, serine and threonine metabolism | bna00260 | 10 | 247 | 0.040486 | 0.758871 | 1 |
| Pyrimidine metabolism | bna00240 | 8 | 201 | 0.039801 | 0.759109 | 1 |
| Phenylalanine, tyrosine and tryptophan biosynthesis | bna00400 | 7 | 178 | 0.039326 | 0.760447 | 1 |
| Limonene and pinene degradation | bna00903 | 1 | 30 | 0.033333 | 0.771228 | 1 |
| Fatty acid biosynthesis | bna00061 | 6 | 159 | 0.037736 | 0.782967 | 1 |
| C5-Branched dibasic acid metabolism | bna00660 | 1 | 32 | 0.03125 | 0.791998 | 1 |
| One carbon pool by folate | bna00670 | 2 | 65 | 0.030769 | 0.824071 | 1 |
| Fatty acid degradation | bna00071 | 6 | 169 | 0.035503 | 0.827109 | 1 |
| Ether lipid metabolism | bna00565 | 3 | 94 | 0.031915 | 0.833922 | 1 |
| Biotin metabolism | bna00780 | 2 | 67 | 0.029851 | 0.83639 | 1 |
| Carbon metabolism | bna01200 | 40 | 972 | 0.041152 | 0.871883 | 1 |
| RNA degradation | bna03018 | 16 | 425 | 0.037647 | 0.873619 | 1 |
| Other types of O-glycan biosynthesis | bna00514 | 1 | 44 | 0.022727 | 0.882499 | 1 |
| Sphingolipid metabolism | bna00600 | 2 | 86 | 0.023256 | 0.919718 | 1 |
| Aminoacyl-tRNA biosynthesis | bna00970 | 8 | 254 | 0.031496 | 0.922928 | 1 |
| Lysine biosynthesis | bna00300 | 1 | 57 | 0.017544 | 0.936713 | 1 |
| Lysine degradation | bna00310 | 3 | 134 | 0.022388 | 0.956087 | 1 |
| Selenocompound metabolism | bna00450 | 1 | 65 | 0.015385 | 0.956755 | 1 |
| Glyoxylate and dicarboxylate metabolism | bna00630 | 8 | 282 | 0.028369 | 0.961479 | 1 |
| Glycosylphosphatidylinositol (GPI)-anchor biosynthesis | bna00563 | 1 | 69 | 0.014493 | 0.964253 | 1 |
| Histidine metabolism | bna00340 | 1 | 74 | 0.013514 | 0.971825 | 1 |
| Terpenoid backbone biosynthesis | bna00900 | 4 | 181 | 0.022099 | 0.974417 | 1 |
| Protein export | bna03060 | 4 | 186 | 0.021505 | 0.978358 | 1 |
| Citrate cycle (TCA cycle) | bna00020 | 5 | 222 | 0.022523 | 0.98166 | 1 |
| SNARE interactions in vesicular transport | bna04130 | 3 | 163 | 0.018405 | 0.984589 | 1 |
| N-Glycan biosynthesis | bna00510 | 3 | 172 | 0.017442 | 0.98898 | 1 |
| RNA polymerase | bna03020 | 2 | 156 | 0.012821 | 0.995289 | 1 |
| mRNA surveillance pathway | bna03015 | 11 | 454 | 0.024229 | 0.99626 | 1 |
| Ubiquitin mediated proteolysis | bna04120 | 11 | 477 | 0.023061 | 0.998029 | 1 |
| Mismatch repair | bna03430 | 4 | 268 | 0.014925 | 0.998846 | 1 |
| DNA replication | bna03030 | 5 | 313 | 0.015974 | 0.999174 | 1 |
| Autophagy - other | bna04136 | 1 | 151 | 0.006623 | 0.99928 | 1 |
| Homologous recombination | bna03440 | 5 | 324 | 0.015432 | 0.999445 | 1 |
| Nucleotide excision repair | bna03420 | 5 | 342 | 0.01462 | 0.999713 | 1 |
| Phagosome | bna04145 | 6 | 379 | 0.015831 | 0.999725 | 1 |
| Oxidative phosphorylation | bna00190 | 12 | 594 | 0.020202 | 0.999845 | 1 |
| Ribosome biogenesis in eukaryotes | bna03008 | 4 | 331 | 0.012085 | 0.999898 | 1 |
| RNA transport | bna03013 | 9 | 621 | 0.014493 | 0.999998 | 1 |
| Ribosome | bna03010 | 8 | 1341 | 0.005966 | 1 | 1 |

Table S6 KEGG pathway analysis of differentially expressed genes in roots of canola seedlings subjected to 24 h salt stress treatment compared to control (0 h).

| Pathway | ID | count | Background number | P-Value | Corrected P-Value | Enrich ratio |
| --- | --- | --- | --- | --- | --- | --- |
| Metabolic pathways | bna01100 | 506 | 7939 | 6.28E-26 | 7.66E-24 | 0.063736 |
| Protein processing in endoplasmic reticulum | bna04141 | 92 | 707 | 3.86E-21 | 2.35E-19 | 0.130127 |
| Biosynthesis of secondary metabolites | bna01110 | 278 | 3914 | 5.47E-20 | 2.22E-18 | 0.071027 |
| Zeatin biosynthesis | bna00908 | 26 | 107 | 4.72E-12 | 1.44E-10 | 0.242991 |
| Phenylpropanoid biosynthesis | bna00940 | 65 | 632 | 3.11E-11 | 7.59E-10 | 0.102848 |
| Starch and sucrose metabolism | bna00500 | 55 | 549 | 2.28E-09 | 4.64E-08 | 0.100182 |
| Galactose metabolism | bna00052 | 28 | 176 | 3.85E-09 | 6.71E-08 | 0.159091 |
| Plant hormone signal transduction | bna04075 | 88 | 1187 | 5.18E-08 | 7.90E-07 | 0.074136 |
| Spliceosome | bna03040 | 54 | 619 | 2.10E-07 | 2.85E-06 | 0.087237 |
| Cyanoamino acid metabolism | bna00460 | 24 | 232 | 4.22E-05 | 0.000515 | 0.103448 |
| Endocytosis | bna04144 | 45 | 593 | 5.07E-05 | 0.000563 | 0.075885 |
| MAPK signaling pathway - plant | bna04016 | 42 | 558 | 0.000104 | 0.000997 | 0.075269 |
| Nitrogen metabolism | bna00910 | 18 | 156 | 0.000106 | 0.000997 | 0.115385 |
| Plant-pathogen interaction | bna04626 | 46 | 643 | 0.000151 | 0.001314 | 0.07154 |
| Amino sugar and nucleotide sugar metabolism | bna00520 | 34 | 457 | 0.00054 | 0.004391 | 0.074398 |
| Fructose and mannose metabolism | bna00051 | 20 | 225 | 0.001035 | 0.007894 | 0.088889 |
| alpha-Linolenic acid metabolism | bna00592 | 16 | 164 | 0.001315 | 0.009138 | 0.097561 |
| Glucosinolate biosynthesis | bna00966 | 8 | 50 | 0.001397 | 0.009138 | 0.16 |
| Inositol phosphate metabolism | bna00562 | 22 | 266 | 0.001423 | 0.009138 | 0.082707 |
| Glutathione metabolism | bna00480 | 27 | 360 | 0.001698 | 0.010351 | 0.075 |
| Sulfur metabolism | bna00920 | 15 | 154 | 0.001858 | 0.010351 | 0.097403 |
| Vitamin B6 metabolism | bna00750 | 8 | 53 | 0.001944 | 0.010351 | 0.150943 |
| Tyrosine metabolism | bna00350 | 14 | 139 | 0.001951 | 0.010351 | 0.100719 |
| Alanine, aspartate and glutamate metabolism | bna00250 | 17 | 200 | 0.003611 | 0.018355 | 0.085 |
| Glycerolipid metabolism | bna00561 | 22 | 293 | 0.004239 | 0.020688 | 0.075085 |
| Glycolysis / Gluconeogenesis | bna00010 | 29 | 429 | 0.004757 | 0.022322 | 0.067599 |
| Biosynthesis of secondary metabolites - other antibiotics | bna00998 | 3 | 7 | 0.005183 | 0.023421 | 0.428571 |
| Valine, leucine and isoleucine degradation | bna00280 | 14 | 162 | 0.006833 | 0.029773 | 0.08642 |
| Phosphatidylinositol signaling system | bna04070 | 20 | 275 | 0.00845 | 0.03476 | 0.072727 |
| Arginine and proline metabolism | bna00330 | 16 | 202 | 0.008548 | 0.03476 | 0.079208 |
| Cysteine and methionine metabolism | bna00270 | 28 | 435 | 0.009846 | 0.038418 | 0.064368 |
| AGE-RAGE signaling pathway in diabetic complications | bna04933 | 9 | 87 | 0.010077 | 0.038418 | 0.103448 |
| Isoflavonoid biosynthesis | bna00943 | 3 | 10 | 0.011357 | 0.041985 | 0.3 |
| Folate biosynthesis | bna00790 | 8 | 76 | 0.013563 | 0.048666 | 0.105263 |
| Biosynthesis of amino acids | bna01230 | 49 | 893 | 0.014703 | 0.05125 | 0.054871 |
| Tryptophan metabolism | bna00380 | 15 | 200 | 0.016268 | 0.05513 | 0.075 |
| Sesquiterpenoid and triterpenoid biosynthesis | bna00909 | 6 | 50 | 0.018143 | 0.059821 | 0.12 |
| ABC transporters | bna02010 | 8 | 82 | 0.019855 | 0.062663 | 0.097561 |
| Pentose phosphate pathway | bna00030 | 14 | 187 | 0.020032 | 0.062663 | 0.074866 |
| Porphyrin and chlorophyll metabolism | bna00860 | 13 | 170 | 0.021145 | 0.064492 | 0.076471 |
| Tropane, piperidine and pyridine alkaloid biosynthesis | bna00960 | 10 | 119 | 0.023742 | 0.070646 | 0.084034 |
| Ubiquinone and other terpenoid-quinone biosynthesis | bna00130 | 11 | 142 | 0.029826 | 0.086637 | 0.077465 |
| Caffeine metabolism | bna00232 | 2 | 7 | 0.042457 | 0.120459 | 0.285714 |
| Taurine and hypotaurine metabolism | bna00430 | 6 | 63 | 0.044702 | 0.123946 | 0.095238 |
| Phenylalanine metabolism | bna00360 | 9 | 117 | 0.048072 | 0.128624 | 0.076923 |
| Pyruvate metabolism | bna00620 | 18 | 294 | 0.048497 | 0.128624 | 0.061224 |
| Pentose and glucuronate interconversions | bna00040 | 25 | 443 | 0.050693 | 0.131587 | 0.056433 |
| Biotin metabolism | bna00780 | 6 | 67 | 0.056112 | 0.142618 | 0.089552 |
| Carbon fixation in photosynthetic organisms | bna00710 | 16 | 262 | 0.061112 | 0.152156 | 0.061069 |
| Ascorbate and aldarate metabolism | bna00053 | 12 | 187 | 0.072936 | 0.177964 | 0.064171 |
| Histidine metabolism | bna00340 | 6 | 74 | 0.079925 | 0.191193 | 0.081081 |
| Riboflavin metabolism | bna00740 | 5 | 59 | 0.091893 | 0.215596 | 0.084746 |
| Steroid biosynthesis | bna00100 | 9 | 136 | 0.09615 | 0.221326 | 0.066176 |
| 2-Oxocarboxylic acid metabolism | bna01210 | 15 | 266 | 0.109881 | 0.24825 | 0.056391 |
| Nicotinate and nicotinamide metabolism | bna00760 | 5 | 65 | 0.122198 | 0.271058 | 0.076923 |
| Linoleic acid metabolism | bna00591 | 4 | 48 | 0.130283 | 0.28383 | 0.083333 |
| Thiamine metabolism | bna00730 | 5 | 69 | 0.144658 | 0.309618 | 0.072464 |
| Glycosphingolipid biosynthesis - lacto and neolacto series | bna00601 | 2 | 18 | 0.171535 | 0.358189 | 0.111111 |
| Glycerophospholipid metabolism | bna00564 | 19 | 380 | 0.173223 | 0.358189 | 0.05 |
| Butanoate metabolism | bna00650 | 5 | 75 | 0.181322 | 0.368689 | 0.066667 |
| Biosynthesis of unsaturated fatty acids | bna01040 | 5 | 76 | 0.187743 | 0.375486 | 0.065789 |
| Isoquinoline alkaloid biosynthesis | bna00950 | 5 | 77 | 0.194245 | 0.382223 | 0.064935 |
| Purine metabolism | bna00230 | 16 | 324 | 0.210538 | 0.407709 | 0.049383 |
| Brassinosteroid biosynthesis | bna00905 | 3 | 40 | 0.217804 | 0.415188 | 0.075 |
| Carbon metabolism | bna01200 | 43 | 972 | 0.226834 | 0.42575 | 0.044239 |
| Valine, leucine and isoleucine biosynthesis | bna00290 | 5 | 84 | 0.241737 | 0.446846 | 0.059524 |
| Fatty acid metabolism | bna01212 | 12 | 245 | 0.259593 | 0.472691 | 0.04898 |
| Fatty acid biosynthesis | bna00061 | 8 | 159 | 0.290585 | 0.521344 | 0.050314 |
| Cutin, suberine and wax biosynthesis | bna00073 | 6 | 114 | 0.295211 | 0.521967 | 0.052632 |
| Glyoxylate and dicarboxylate metabolism | bna00630 | 13 | 282 | 0.315796 | 0.550387 | 0.046099 |
| Other glycan degradation | bna00511 | 3 | 51 | 0.330089 | 0.567195 | 0.058824 |
| Propanoate metabolism | bna00640 | 6 | 123 | 0.354808 | 0.599217 | 0.04878 |
| Carotenoid biosynthesis | bna00906 | 5 | 100 | 0.358548 | 0.599217 | 0.05 |
| Peroxisome | bna04146 | 14 | 318 | 0.365221 | 0.602122 | 0.044025 |
| Flavonoid biosynthesis | bna00941 | 5 | 105 | 0.39588 | 0.629451 | 0.047619 |
| Fatty acid elongation | bna00062 | 5 | 105 | 0.39588 | 0.629451 | 0.047619 |
| Phenylalanine, tyrosine and tryptophan biosynthesis | bna00400 | 8 | 178 | 0.397277 | 0.629451 | 0.044944 |
| Glycosphingolipid biosynthesis - globo and isoglobo series | bna00603 | 2 | 35 | 0.406333 | 0.635547 | 0.057143 |
| Lysine degradation | bna00310 | 6 | 134 | 0.428497 | 0.66173 | 0.044776 |
| Phosphonate and phosphinate metabolism | bna00440 | 2 | 38 | 0.445306 | 0.679091 | 0.052632 |
| Synthesis and degradation of ketone bodies | bna00072 | 1 | 16 | 0.477793 | 0.719639 | 0.0625 |
| Fatty acid degradation | bna00071 | 7 | 169 | 0.490835 | 0.730267 | 0.04142 |
| Glycine, serine and threonine metabolism | bna00260 | 10 | 247 | 0.497417 | 0.730901 | 0.040486 |
| Ether lipid metabolism | bna00565 | 4 | 94 | 0.503243 | 0.730901 | 0.042553 |
| Pyrimidine metabolism | bna00240 | 8 | 201 | 0.526324 | 0.75543 | 0.039801 |
| Monobactam biosynthesis | bna00261 | 2 | 46 | 0.541667 | 0.768411 | 0.043478 |
| Arginine biosynthesis | bna00220 | 5 | 130 | 0.573881 | 0.804716 | 0.038462 |
| Circadian rhythm - plant | bna04712 | 5 | 131 | 0.580451 | 0.804716 | 0.038168 |
| Photosynthesis - antenna proteins | bna00196 | 3 | 78 | 0.589511 | 0.808094 | 0.038462 |
| Glycosaminoglycan degradation | bna00531 | 1 | 25 | 0.629791 | 0.851837 | 0.04 |
| Stilbenoid, diarylheptanoid and gingerol biosynthesis | bna00945 | 2 | 55 | 0.635387 | 0.851837 | 0.036364 |
| Biosynthesis of secondary metabolites - unclassified | bna00999 | 2 | 57 | 0.654052 | 0.866286 | 0.035088 |
| beta-Alanine metabolism | bna00410 | 6 | 172 | 0.660365 | 0.866286 | 0.034884 |
| Limonene and pinene degradation | bna00903 | 1 | 30 | 0.694195 | 0.900976 | 0.033333 |
| Diterpenoid biosynthesis | bna00904 | 3 | 93 | 0.702669 | 0.902375 | 0.032258 |
| C5-Branched dibasic acid metabolism | bna00660 | 1 | 32 | 0.716703 | 0.906987 | 0.03125 |
| Selenocompound metabolism | bna00450 | 2 | 65 | 0.721129 | 0.906987 | 0.030769 |
| Sulfur relay system | bna04122 | 1 | 37 | 0.76599 | 0.944803 | 0.027027 |
| RNA degradation | bna03018 | 14 | 425 | 0.766685 | 0.944803 | 0.032941 |
| Pantothenate and CoA biosynthesis | bna00770 | 3 | 108 | 0.790347 | 0.964223 | 0.027778 |
| Photosynthesis | bna00195 | 7 | 245 | 0.836472 | 1 | 0.028571 |
| SNARE interactions in vesicular transport | bna04130 | 4 | 163 | 0.87565 | 1 | 0.02454 |
| Lysine biosynthesis | bna00300 | 1 | 57 | 0.891066 | 1 | 0.017544 |
| One carbon pool by folate | bna00670 | 1 | 65 | 0.919773 | 1 | 0.015385 |
| Arachidonic acid metabolism | bna00590 | 1 | 69 | 0.931152 | 1 | 0.014493 |
| Aminoacyl-tRNA biosynthesis | bna00970 | 4 | 254 | 0.988123 | 1 | 0.015748 |
| Citrate cycle (TCA cycle) | bna00020 | 3 | 222 | 0.991193 | 1 | 0.013514 |
| Terpenoid backbone biosynthesis | bna00900 | 2 | 181 | 0.99257 | 1 | 0.01105 |
| Protein export | bna03060 | 2 | 186 | 0.993717 | 1 | 0.010753 |
| Ubiquitin mediated proteolysis | bna04120 | 9 | 477 | 0.994572 | 1 | 0.018868 |
| Autophagy - other | bna04136 | 1 | 151 | 0.99701 | 1 | 0.006623 |
| Homologous recombination | bna03440 | 4 | 324 | 0.998434 | 1 | 0.012346 |
| Basal transcription factors | bna03022 | 1 | 171 | 0.998609 | 1 | 0.005848 |
| DNA replication | bna03030 | 3 | 313 | 0.9995 | 1 | 0.009585 |
| RNA transport | bna03013 | 10 | 621 | 0.99957 | 1 | 0.016103 |
| Mismatch repair | bna03430 | 2 | 268 | 0.999623 | 1 | 0.007463 |
| Nucleotide excision repair | bna03420 | 3 | 342 | 0.999806 | 1 | 0.008772 |
| Phagosome | bna04145 | 3 | 379 | 0.999943 | 1 | 0.007916 |
| mRNA surveillance pathway | bna03015 | 4 | 454 | 0.999973 | 1 | 0.008811 |
| Ribosome biogenesis in eukaryotes | bna03008 | 1 | 331 | 0.999997 | 1 | 0.003021 |
| Oxidative phosphorylation | bna00190 | 3 | 594 | 1 | 1 | 0.005051 |
| Ribosome | bna03010 | 11 | 1341 | 1 | 1 | 0.008203 |

Table S7 KEGG pathway analysis of differentially expressed genes in roots of canola seedlings subjected to 72 h salt stress treatment compared to control (0 h).

| Pathway | ID | Count | Background number | P-Value | Corrected P-Value | Enrich ratio |
| --- | --- | --- | --- | --- | --- | --- |
| Metabolic pathways | bna01100 | 740 | 7939 | 7.08E-22 | 8.99E-20 | 0.093211 |
| Phenylpropanoid biosynthesis | bna00940 | 115 | 632 | 5.57E-20 | 3.53E-18 | 0.181962 |
| Biosynthesis of secondary metabolites | bna01110 | 397 | 3914 | 6.60E-17 | 2.79E-15 | 0.101431 |
| Zeatin biosynthesis | bna00908 | 32 | 107 | 4.73E-11 | 1.50E-09 | 0.299065 |
| Galactose metabolism | bna00052 | 36 | 176 | 2.10E-08 | 5.33E-07 | 0.204545 |
| Plant hormone signal transduction | bna04075 | 128 | 1187 | 1.15E-07 | 2.44E-06 | 0.107835 |
| Glucosinolate biosynthesis | bna00966 | 15 | 50 | 6.03E-06 | 0.000109 | 0.3 |
| Amino sugar and nucleotide sugar metabolism | bna00520 | 57 | 457 | 9.32E-06 | 0.000148 | 0.124726 |
| alpha-Linolenic acid metabolism | bna00592 | 28 | 164 | 1.51E-05 | 0.000213 | 0.170732 |
| Plant-pathogen interaction | bna04626 | 72 | 643 | 2.01E-05 | 0.000255 | 0.111975 |
| Glycerolipid metabolism | bna00561 | 40 | 293 | 3.19E-05 | 0.000368 | 0.136519 |
| Linoleic acid metabolism | bna00591 | 13 | 48 | 6.07E-05 | 0.000642 | 0.270833 |
| Starch and sucrose metabolism | bna00500 | 61 | 549 | 9.92E-05 | 0.000969 | 0.111111 |
| Protein processing in endoplasmic reticulum | bna04141 | 74 | 707 | 0.000111 | 0.001003 | 0.104668 |
| Cyanoamino acid metabolism | bna00460 | 32 | 232 | 0.000157 | 0.001326 | 0.137931 |
| MAPK signaling pathway - plant | bna04016 | 60 | 558 | 0.000247 | 0.001959 | 0.107527 |
| Ubiquinone and other terpenoid-quinone biosynthesis | bna00130 | 21 | 142 | 0.000896 | 0.006691 | 0.147887 |
| Valine, leucine and isoleucine biosynthesis | bna00290 | 14 | 84 | 0.002322 | 0.015997 | 0.166667 |
| Butanoate metabolism | bna00650 | 13 | 75 | 0.002421 | 0.015997 | 0.173333 |
| Nitrogen metabolism | bna00910 | 21 | 156 | 0.002519 | 0.015997 | 0.134615 |
| Valine, leucine and isoleucine degradation | bna00280 | 21 | 162 | 0.003748 | 0.022667 | 0.12963 |
| Sulfur metabolism | bna00920 | 20 | 154 | 0.004508 | 0.026025 | 0.12987 |
| Flavonoid biosynthesis | bna00941 | 15 | 105 | 0.006024 | 0.033265 | 0.142857 |
| Alanine, aspartate and glutamate metabolism | bna00250 | 23 | 200 | 0.009114 | 0.048228 | 0.115 |
| Biosynthesis of secondary metabolites - other antibiotics | bna00998 | 3 | 7 | 0.019265 | 0.096698 | 0.428571 |
| Pentose and glucuronate interconversions | bna00040 | 41 | 443 | 0.019796 | 0.096698 | 0.092551 |
| Cysteine and methionine metabolism | bna00270 | 40 | 435 | 0.022961 | 0.105156 | 0.091954 |
| Cutin, suberine and wax biosynthesis | bna00073 | 14 | 114 | 0.023184 | 0.105156 | 0.122807 |
| Taurine and hypotaurine metabolism | bna00430 | 9 | 63 | 0.029361 | 0.128456 | 0.142857 |
| Arginine and proline metabolism | bna00330 | 21 | 202 | 0.030344 | 0.128456 | 0.10396 |
| 2-Oxocarboxylic acid metabolism | bna01210 | 26 | 266 | 0.032495 | 0.133126 | 0.097744 |
| Glycosphingolipid biosynthesis - globo and isoglobo series | bna00603 | 6 | 35 | 0.035643 | 0.141458 | 0.171429 |
| Glutathione metabolism | bna00480 | 33 | 360 | 0.037109 | 0.142815 | 0.091667 |
| Endocytosis | bna04144 | 50 | 593 | 0.043358 | 0.161954 | 0.084317 |
| Tryptophan metabolism | bna00380 | 20 | 200 | 0.046182 | 0.167575 | 0.1 |
| Inositol phosphate metabolism | bna00562 | 25 | 266 | 0.050392 | 0.176843 | 0.093985 |
| ABC transporters | bna02010 | 10 | 82 | 0.051521 | 0.176843 | 0.121951 |
| Sesquiterpenoid and triterpenoid biosynthesis | bna00909 | 7 | 50 | 0.0555 | 0.185486 | 0.14 |
| Glycolysis / Gluconeogenesis | bna00010 | 37 | 429 | 0.057371 | 0.186823 | 0.086247 |
| Glycerophospholipid metabolism | bna00564 | 33 | 380 | 0.064688 | 0.203081 | 0.086842 |
| Carbon fixation in photosynthetic organisms | bna00710 | 24 | 262 | 0.067135 | 0.203081 | 0.091603 |
| beta-Alanine metabolism | bna00410 | 17 | 172 | 0.067161 | 0.203081 | 0.098837 |
| AGE-RAGE signaling pathway in diabetic complications | bna04933 | 10 | 87 | 0.06889 | 0.203465 | 0.114943 |
| Carotenoid biosynthesis | bna00906 | 11 | 100 | 0.073171 | 0.211199 | 0.11 |
| Circadian rhythm - plant | bna04712 | 13 | 131 | 0.097501 | 0.27517 | 0.099237 |
| Tropane, piperidine and pyridine alkaloid biosynthesis | bna00960 | 12 | 119 | 0.099846 | 0.275663 | 0.10084 |
| Riboflavin metabolism | bna00740 | 7 | 59 | 0.103395 | 0.279386 | 0.118644 |
| Pantothenate and CoA biosynthesis | bna00770 | 11 | 108 | 0.106509 | 0.281804 | 0.101852 |
| Pyruvate metabolism | bna00620 | 25 | 294 | 0.113365 | 0.293824 | 0.085034 |
| Glycosphingolipid biosynthesis - lacto and neolacto series | bna00601 | 3 | 18 | 0.13085 | 0.33236 | 0.166667 |
| Brassinosteroid biosynthesis | bna00905 | 5 | 40 | 0.134376 | 0.334623 | 0.125 |
| Vitamin B6 metabolism | bna00750 | 6 | 53 | 0.14556 | 0.355501 | 0.113208 |
| Fructose and mannose metabolism | bna00051 | 19 | 225 | 0.157227 | 0.376751 | 0.084444 |
| Stilbenoid, diarylheptanoid and gingerol biosynthesis | bna00945 | 6 | 55 | 0.162663 | 0.38256 | 0.109091 |
| C5-Branched dibasic acid metabolism | bna00660 | 4 | 32 | 0.171238 | 0.388666 | 0.125 |
| Ether lipid metabolism | bna00565 | 9 | 94 | 0.17138 | 0.388666 | 0.095745 |
| Biosynthesis of secondary metabolites - unclassified | bna00999 | 6 | 57 | 0.180577 | 0.402338 | 0.105263 |
| Phosphatidylinositol signaling system | bna04070 | 22 | 275 | 0.192166 | 0.420778 | 0.08 |
| Steroid biosynthesis | bna00100 | 11 | 136 | 0.277426 | 0.597172 | 0.080882 |
| Spliceosome | bna03040 | 43 | 619 | 0.337632 | 0.714655 | 0.069467 |
| Photosynthesis - antenna proteins | bna00196 | 6 | 78 | 0.398998 | 0.8307 | 0.076923 |
| Fatty acid degradation | bna00071 | 12 | 169 | 0.414839 | 0.849752 | 0.071006 |
| Ascorbate and aldarate metabolism | bna00053 | 13 | 187 | 0.436067 | 0.86532 | 0.069519 |
| Pentose phosphate pathway | bna00030 | 13 | 187 | 0.436067 | 0.86532 | 0.069519 |
| Arachidonic acid metabolism | bna00590 | 5 | 69 | 0.466534 | 0.911536 | 0.072464 |
| Base excision repair | bna03410 | 8 | 116 | 0.478187 | 0.920147 | 0.068966 |
| Glycosaminoglycan degradation | bna00531 | 2 | 25 | 0.492249 | 0.933069 | 0.08 |
| Lysine biosynthesis | bna00300 | 4 | 57 | 0.508555 | 0.949801 | 0.070175 |
| Fatty acid elongation | bna00062 | 7 | 105 | 0.520385 | 0.95781 | 0.066667 |
| Oxidative phosphorylation | bna00190 | 38 | 594 | 0.539944 | 0.965096 | 0.063973 |
| Tyrosine metabolism | bna00350 | 9 | 139 | 0.542536 | 0.965096 | 0.064748 |
| Folate biosynthesis | bna00790 | 5 | 76 | 0.547141 | 0.965096 | 0.065789 |
| Monobactam biosynthesis | bna00261 | 3 | 46 | 0.575819 | 1 | 0.065217 |
| Phenylalanine metabolism | bna00360 | 7 | 117 | 0.628859 | 1 | 0.059829 |
| Biotin metabolism | bna00780 | 4 | 67 | 0.629551 | 1 | 0.059701 |
| Non-homologous end-joining | bna03450 | 1 | 15 | 0.631671 | 1 | 0.066667 |
| Sphingolipid metabolism | bna00600 | 5 | 86 | 0.650923 | 1 | 0.05814 |
| Thiamine metabolism | bna00730 | 4 | 69 | 0.651323 | 1 | 0.057971 |
| Synthesis and degradation of ketone bodies | bna00072 | 1 | 16 | 0.653962 | 1 | 0.0625 |
| Porphyrin and chlorophyll metabolism | bna00860 | 10 | 170 | 0.654388 | 1 | 0.058824 |
| Propanoate metabolism | bna00640 | 7 | 123 | 0.677552 | 1 | 0.056911 |
| Biosynthesis of amino acids | bna01230 | 54 | 893 | 0.693609 | 1 | 0.06047 |
| RNA degradation | bna03018 | 25 | 425 | 0.698507 | 1 | 0.058824 |
| Nicotinate and nicotinamide metabolism | bna00760 | 3 | 65 | 0.787385 | 1 | 0.046154 |
| Photosynthesis | bna00195 | 13 | 245 | 0.788432 | 1 | 0.053061 |
| Arginine biosynthesis | bna00220 | 6 | 130 | 0.837439 | 1 | 0.046154 |
| Other glycan degradation | bna00511 | 2 | 51 | 0.83861 | 1 | 0.039216 |
| DNA replication | bna03030 | 16 | 313 | 0.847103 | 1 | 0.051118 |
| Histidine metabolism | bna00340 | 3 | 74 | 0.852084 | 1 | 0.040541 |
| Monoterpenoid biosynthesis | bna00902 | 1 | 30 | 0.855614 | 1 | 0.033333 |
| Limonene and pinene degradation | bna00903 | 1 | 30 | 0.855614 | 1 | 0.033333 |
| Lysine degradation | bna00310 | 6 | 134 | 0.856569 | 1 | 0.044776 |
| Biosynthesis of unsaturated fatty acids | bna01040 | 3 | 76 | 0.863883 | 1 | 0.039474 |
| Carbon metabolism | bna01200 | 53 | 972 | 0.897166 | 1 | 0.054527 |
| Fatty acid metabolism | bna01212 | 11 | 245 | 0.910664 | 1 | 0.044898 |
| Phosphonate and phosphinate metabolism | bna00440 | 1 | 38 | 0.912383 | 1 | 0.026316 |
| Glycine, serine and threonine metabolism | bna00260 | 11 | 247 | 0.91537 | 1 | 0.044534 |
| Autophagy - other | bna04136 | 6 | 151 | 0.918261 | 1 | 0.039735 |
| Selenocompound metabolism | bna00450 | 2 | 65 | 0.918914 | 1 | 0.030769 |
| Diterpenoid biosynthesis | bna00904 | 3 | 93 | 0.934879 | 1 | 0.032258 |
| Peroxisome | bna04146 | 14 | 318 | 0.942186 | 1 | 0.044025 |
| Isoquinoline alkaloid biosynthesis | bna00950 | 2 | 77 | 0.956103 | 1 | 0.025974 |
| Glyoxylate and dicarboxylate metabolism | bna00630 | 11 | 282 | 0.969399 | 1 | 0.039007 |
| Pyrimidine metabolism | bna00240 | 7 | 201 | 0.970925 | 1 | 0.034826 |
| Fatty acid biosynthesis | bna00061 | 5 | 159 | 0.972855 | 1 | 0.031447 |
| Aminoacyl-tRNA biosynthesis | bna00970 | 9 | 254 | 0.98001 | 1 | 0.035433 |
| Basal transcription factors | bna03022 | 5 | 171 | 0.983463 | 1 | 0.02924 |
| One carbon pool by folate | bna00670 | 1 | 65 | 0.98377 | 1 | 0.015385 |
| N-Glycan biosynthesis | bna00510 | 5 | 172 | 0.984143 | 1 | 0.02907 |
| Purine metabolism | bna00230 | 12 | 324 | 0.984492 | 1 | 0.037037 |
| Glycosylphosphatidylinositol (GPI)-anchor biosynthesis | bna00563 | 1 | 69 | 0.987358 | 1 | 0.014493 |
| Phenylalanine, tyrosine and tryptophan biosynthesis | bna00400 | 5 | 178 | 0.987701 | 1 | 0.02809 |
| Mismatch repair | bna03430 | 9 | 268 | 0.987755 | 1 | 0.033582 |
| RNA polymerase | bna03020 | 4 | 156 | 0.988861 | 1 | 0.025641 |
| Protein export | bna03060 | 5 | 186 | 0.991282 | 1 | 0.026882 |
| Ubiquitin mediated proteolysis | bna04120 | 18 | 477 | 0.993954 | 1 | 0.037736 |
| Citrate cycle (TCA cycle) | bna00020 | 6 | 222 | 0.994771 | 1 | 0.027027 |
| Terpenoid backbone biosynthesis | bna00900 | 4 | 181 | 0.996532 | 1 | 0.022099 |
| Homologous recombination | bna03440 | 9 | 324 | 0.998528 | 1 | 0.027778 |
| Phagosome | bna04145 | 11 | 379 | 0.99895 | 1 | 0.029024 |
| Nucleotide excision repair | bna03420 | 9 | 342 | 0.999288 | 1 | 0.026316 |
| SNARE interactions in vesicular transport | bna04130 | 2 | 163 | 0.999611 | 1 | 0.01227 |
| Ribosome biogenesis in eukaryotes | bna03008 | 5 | 331 | 0.999992 | 1 | 0.015106 |
| mRNA surveillance pathway | bna03015 | 9 | 454 | 0.999995 | 1 | 0.019824 |
| Proteasome | bna03050 | 1 | 224 | 0.999999 | 1 | 0.004464 |
| RNA transport | bna03013 | 7 | 621 | 1 | 1 | 0.011272 |
| Ribosome | bna03010 | 24 | 1341 | 1 | 1 | 0.017897 |

Table S8 Significant enrichment pathways of DEGs KEGG pathway analysis in roots of canola seedlings subjected to 2 h, 24 h, 72 h salt stress treatment compared to control (0 h).

|  | #Term | ID | Input number | Back-  ground number | Enrich  ratio | P-Value | Number of  up-regulated genes | Up-regulated  gene ratio |
| --- | --- | --- | --- | --- | --- | --- | --- | --- |
| 2 h | Metabolic pathways | bna01100 | 566 | 7939 | 0.071 | 2.65E-18 | 366 | 0.647 |
|  | Phenylpropanoid biosynthesis | bna00940 | 89 | 632 | 0.141 | 1.09E-16 | 37 | 0.416 |
|  | Plant hormone signal transduction | bna04075 | 117 | 1187 | 0.099 | 1.70E-11 | 48 | 0.41 |
|  | Protein processing in endoplasmic reticulum | bna04141 | 81 | 707 | 0.115 | 3.80E-11 | 79 | 0.975 |
|  | Biosynthesis of secondary metabolites | bna01110 | 287 | 3914 | 0.073 | 6.70E-11 | 177 | 0.617 |
|  | Zeatin biosynthesis | bna00908 | 23 | 107 | 0.215 | 3.42E-08 | 18 | 0.783 |
|  | Galactose metabolism | bna00052 | 26 | 176 | 0.148 | 2.90E-06 | 25 | 0.962 |
|  | Starch and sucrose metabolism | bna00500 | 51 | 549 | 0.093 | 3.20E-05 | 39 | 0.765 |
|  | Plant-pathogen interaction | bna04626 | 56 | 643 | 0.087 | 6.90E-05 | 24 | 0.429 |
|  | Nitrogen metabolism | bna00910 | 21 | 156 | 0.135 | 8.32E-05 | 6 | 0.286 |
|  | Pentose and glucuronate interconversions | bna00040 | 42 | 443 | 0.095 | 0.0001 | 27 | 0.643 |
|  | Spliceosome | bna03040 | 53 | 619 | 0.086 | 0.00016 | 53 | 1 |
|  | Taurine and hypotaurine metabolism | bna00430 | 12 | 63 | 0.19 | 0.00017 | 10 | 0.833 |
|  | Sulfur metabolism | bna00920 | 20 | 154 | 0.13 | 0.00019 | 6 | 0.3 |
|  | AGE-RAGE signaling pathway in diabetic complications | bna04933 | 14 | 87 | 0.161 | 0.00024 | 8 | 0.571 |
|  | Cysteine and methionine metabolism | bna00270 | 40 | 435 | 0.092 | 0.00026 | 30 | 0.75 |
|  | Alanine, aspartate and glutamate metabolism | bna00250 | 23 | 200 | 0.115 | 0.00032 | 19 | 0.826 |
|  | alpha-Linolenic acid metabolism | bna00592 | 20 | 164 | 0.122 | 0.00039 | 6 | 0.3 |
|  | Linoleic acid metabolism | bna00591 | 9 | 48 | 0.188 | 0.00119 | 2 | 0.222 |
|  | Arginine and proline metabolism | bna00330 | 21 | 202 | 0.104 | 0.00182 | 16 | 0.762 |
|  | Endocytosis | bna04144 | 44 | 593 | 0.074 | 0.00643 | 36 | 0.818 |
|  | Glycerolipid metabolism | bna00561 | 25 | 293 | 0.085 | 0.00783 | 17 | 0.68 |
|  | Stilbenoid, diarylheptanoid and gingerol biosynthesis | bna00945 | 8 | 55 | 0.145 | 0.00858 | 7 | 0.875 |
|  | Inositol phosphate metabolism | bna00562 | 23 | 266 | 0.086 | 0.00907 | 10 | 0.435 |
|  | Riboflavin metabolism | bna00740 | 8 | 59 | 0.136 | 0.01228 | 6 | 0.75 |
|  | Ubiquinone and other terpenoid-quinone biosynthesis | bna00130 | 14 | 142 | 0.099 | 0.01436 | 8 | 0.571 |
|  | Amino sugar and nucleotide sugar metabolism | bna00520 | 34 | 457 | 0.074 | 0.0145 | 20 | 0.588 |
|  | Cutin, suberine and wax biosynthesis | bna00073 | 12 | 114 | 0.105 | 0.0146 | 7 | 0.583 |
|  | MAPK signaling pathway - plant | bna04016 | 40 | 558 | 0.072 | 0.01463 | 27 | 0.675 |
|  | Glucosinolate biosynthesis | bna00966 | 7 | 50 | 0.14 | 0.01611 | 0 | 0 |
|  | Circadian rhythm - plant | bna04712 | 13 | 131 | 0.099 | 0.01709 | 4 | 0.308 |
|  | Flavonoid biosynthesis | bna00941 | 11 | 105 | 0.105 | 0.01941 | 10 | 0.909 |
|  | Vitamin B6 metabolism | bna00750 | 7 | 53 | 0.132 | 0.02089 | 7 | 1 |
|  | Cyanoamino acid metabolism | bna00460 | 19 | 232 | 0.082 | 0.0262 | 12 | 0.632 |
|  | Phosphatidylinositol signaling system | bna04070 | 21 | 275 | 0.076 | 0.03726 | 11 | 0.524 |
|  | Glycolysis / Gluconeogenesis | bna00010 | 30 | 429 | 0.07 | 0.03983 | 27 | 0.9 |
|  | Sesquiterpenoid and triterpenoid biosynthesis | bna00909 | 6 | 50 | 0.12 | 0.04495 | 3 | 0.5 |
| 24 h | Metabolic pathways | bna01100 | 506 | 7939 | 6.28E-26 | 0.064 | 286 | 0.565 |
|  | Protein processing in endoplasmic reticulum | bna04141 | 92 | 707 | 3.86E-21 | 0.13 | 86 | 0.935 |
|  | Biosynthesis of secondary metabolites | bna01110 | 278 | 3914 | 5.47E-20 | 0.071 | 162 | 0.583 |
|  | Zeatin biosynthesis | bna00908 | 26 | 107 | 4.72E-12 | 0.243 | 21 | 0.808 |
|  | Phenylpropanoid biosynthesis | bna00940 | 65 | 632 | 3.11E-11 | 0.103 | 25 | 0.385 |
|  | Starch and sucrose metabolism | bna00500 | 55 | 549 | 2.28E-09 | 0.1 | 26 | 0.473 |
|  | Galactose metabolism | bna00052 | 28 | 176 | 3.85E-09 | 0.159 | 22 | 0.786 |
|  | Plant hormone signal transduction | bna04075 | 88 | 1187 | 5.18E-08 | 0.074 | 37 | 0.42 |
|  | Spliceosome | bna03040 | 54 | 619 | 2.10E-07 | 0.087 | 53 | 0.981 |
|  | Cyanoamino acid metabolism | bna00460 | 24 | 232 | 4.22E-05 | 0.103 | 9 | 0.375 |
|  | Endocytosis | bna04144 | 45 | 593 | 5.07E-05 | 0.076 | 33 | 0.733 |
|  | MAPK signaling pathway - plant | bna04016 | 42 | 558 | 0.0001 | 0.075 | 27 | 0.643 |
|  | Nitrogen metabolism | bna00910 | 18 | 156 | 0.00011 | 0.115 | 3 | 0.167 |
|  | Plant-pathogen interaction | bna04626 | 46 | 643 | 0.00015 | 0.072 | 13 | 0.283 |
|  | Amino sugar and nucleotide sugar metabolism | bna00520 | 34 | 457 | 0.00054 | 0.074 | 25 | 0.735 |
|  | Fructose and mannose metabolism | bna00051 | 20 | 225 | 0.00104 | 0.089 | 18 | 0.9 |
|  | alpha-Linolenic acid metabolism | bna00592 | 16 | 164 | 0.00132 | 0.098 | 5 | 0.313 |
|  | Glucosinolate biosynthesis | bna00966 | 8 | 50 | 0.0014 | 0.16 | 0 | 0 |
|  | Inositol phosphate metabolism | bna00562 | 22 | 266 | 0.00142 | 0.083 | 5 | 0.227 |
|  | Glutathione metabolism | bna00480 | 27 | 360 | 0.0017 | 0.075 | 13 | 0.481 |
|  | Sulfur metabolism | bna00920 | 15 | 154 | 0.00186 | 0.097 | 3 | 0.2 |
|  | Vitamin B6 metabolism | bna00750 | 8 | 53 | 0.00194 | 0.151 | 4 | 0.5 |
|  | Tyrosine metabolism | bna00350 | 14 | 139 | 0.00195 | 0.101 | 10 | 0.714 |
|  | Alanine, aspartate and glutamate metabolism | bna00250 | 17 | 200 | 0.00361 | 0.085 | 14 | 0.824 |
|  | Glycerolipid metabolism | bna00561 | 22 | 293 | 0.00424 | 0.075 | 14 | 0.636 |
|  | Glycolysis / Gluconeogenesis | bna00010 | 29 | 429 | 0.00476 | 0.068 | 23 | 0.793 |
|  | Biosynthesis of secondary metabolites - other antibiotics | bna00998 | 3 | 7 | 0.00518 | 0.429 | 3 | 1 |
|  | Valine, leucine and isoleucine degradation | bna00280 | 14 | 162 | 0.00683 | 0.086 | 13 | 0.929 |
|  | Phosphatidylinositol signaling system | bna04070 | 20 | 275 | 0.00845 | 0.073 | 5 | 0.25 |
|  | Arginine and proline metabolism | bna00330 | 16 | 202 | 0.00855 | 0.079 | 13 | 0.813 |
|  | Cysteine and methionine metabolism | bna00270 | 28 | 435 | 0.00985 | 0.064 | 13 | 0.464 |
|  | AGE-RAGE signaling pathway in diabetic complications | bna04933 | 9 | 87 | 0.01008 | 0.103 | 3 | 0.333 |
|  | Isoflavonoid biosynthesis | bna00943 | 3 | 10 | 0.01136 | 0.3 | 3 | 1 |
|  | Folate biosynthesis | bna00790 | 8 | 76 | 0.01356 | 0.105 | 3 | 0.375 |
|  | Biosynthesis of amino acids | bna01230 | 49 | 893 | 0.0147 | 0.055 | 35 | 0.714 |
|  | Tryptophan metabolism | bna00380 | 15 | 200 | 0.01627 | 0.075 | 9 | 0.6 |
|  | Sesquiterpenoid and triterpenoid biosynthesis | bna00909 | 6 | 50 | 0.01814 | 0.12 | 3 | 0.5 |
|  | ABC transporters | bna02010 | 8 | 82 | 0.01985 | 0.098 | 4 | 0.5 |
|  | Pentose phosphate pathway | bna00030 | 14 | 187 | 0.02003 | 0.075 | 13 | 0.929 |
|  | Porphyrin and chlorophyll metabolism | bna00860 | 13 | 170 | 0.02115 | 0.076 | 12 | 0.923 |
|  | Tropane, piperidine and pyridine alkaloid biosynthesis | bna00960 | 10 | 119 | 0.02374 | 0.084 | 4 | 0.4 |
|  | Ubiquinone and other terpenoid-quinone biosynthesis | bna00130 | 11 | 142 | 0.02983 | 0.077 | 6 | 0.545 |
|  | Caffeine metabolism | bna00232 | 2 | 7 | 0.04246 | 0.286 | 2 | 1 |
|  | Taurine and hypotaurine metabolism | bna00430 | 6 | 63 | 0.0447 | 0.095 | 3 | 0.5 |
|  | Phenylalanine metabolism | bna00360 | 9 | 117 | 0.04807 | 0.077 | 5 | 0.556 |
|  | Pyruvate metabolism | bna00620 | 18 | 294 | 0.0485 | 0.061 | 12 | 0.667 |
| 72 h | Metabolic pathways | bna01100 | 740 | 7939 | 7.08E-22 | 0.093 | 449 | 0.607 |
|  | Phenylpropanoid biosynthesis | bna00940 | 115 | 632 | 5.57E-20 | 0.182 | 45 | 0.391 |
|  | Biosynthesis of secondary metabolites | bna01110 | 397 | 3914 | 6.60E-17 | 0.101 | 235 | 0.592 |
|  | Zeatin biosynthesis | bna00908 | 32 | 107 | 4.73E-11 | 0.299 | 22 | 0.688 |
|  | Galactose metabolism | bna00052 | 36 | 176 | 2.10E-08 | 0.205 | 30 | 0.833 |
|  | Plant hormone signal transduction | bna04075 | 128 | 1187 | 1.15E-07 | 0.108 | 64 | 0.5 |
|  | Glucosinolate biosynthesis | bna00966 | 15 | 50 | 6.03E-06 | 0.3 | 0 | 0 |
|  | Amino sugar and nucleotide sugar metabolism | bna00520 | 57 | 457 | 9.32E-06 | 0.125 | 30 | 0.526 |
|  | alpha-Linolenic acid metabolism | bna00592 | 28 | 164 | 1.51E-05 | 0.171 | 12 | 0.429 |
|  | Plant-pathogen interaction | bna04626 | 72 | 643 | 2.01E-05 | 0.112 | 31 | 0.431 |
|  | Glycerolipid metabolism | bna00561 | 40 | 293 | 3.19E-05 | 0.137 | 32 | 0.8 |
|  | Linoleic acid metabolism | bna00591 | 13 | 48 | 6.07E-05 | 0.271 | 5 | 0.385 |
|  | Starch and sucrose metabolism | bna00500 | 61 | 549 | 9.92E-05 | 0.111 | 39 | 0.639 |
|  | Protein processing in endoplasmic reticulum | bna04141 | 74 | 707 | 0.00011 | 0.105 | 72 | 0.973 |
|  | Cyanoamino acid metabolism | bna00460 | 32 | 232 | 0.00016 | 0.138 | 21 | 0.656 |
|  | MAPK signaling pathway - plant | bna04016 | 60 | 558 | 0.00025 | 0.108 | 38 | 0.633 |
|  | Ubiquinone and other terpenoid-quinone biosynthesis | bna00130 | 21 | 142 | 0.0009 | 0.148 | 16 | 0.762 |
|  | Valine, leucine and isoleucine biosynthesis | bna00290 | 14 | 84 | 0.00232 | 0.167 | 10 | 0.714 |
|  | Butanoate metabolism | bna00650 | 13 | 75 | 0.00242 | 0.173 | 10 | 0.769 |
|  | Nitrogen metabolism | bna00910 | 21 | 156 | 0.00252 | 0.135 | 7 | 0.333 |
|  | Valine, leucine and isoleucine degradation | bna00280 | 21 | 162 | 0.00375 | 0.13 | 19 | 0.905 |
|  | Sulfur metabolism | bna00920 | 20 | 154 | 0.00451 | 0.13 | 8 | 0.4 |
|  | Flavonoid biosynthesis | bna00941 | 15 | 105 | 0.00602 | 0.143 | 8 | 0.533 |
|  | Alanine, aspartate and glutamate metabolism | bna00250 | 23 | 200 | 0.00911 | 0.115 | 18 | 0.783 |
|  | Biosynthesis of secondary metabolites - other antibiotics | bna00998 | 3 | 7 | 0.01926 | 0.429 | 3 | 1 |
|  | Pentose and glucuronate interconversions | bna00040 | 41 | 443 | 0.0198 | 0.093 | 21 | 0.512 |
|  | Cysteine and methionine metabolism | bna00270 | 40 | 435 | 0.02296 | 0.092 | 28 | 0.7 |
|  | Cutin, suberine and wax biosynthesis | bna00073 | 14 | 114 | 0.02318 | 0.123 | 9 | 0.643 |
|  | Taurine and hypotaurine metabolism | bna00430 | 9 | 63 | 0.02936 | 0.143 | 6 | 0.667 |
|  | Arginine and proline metabolism | bna00330 | 21 | 202 | 0.03034 | 0.104 | 12 | 0.571 |
|  | 2-Oxocarboxylic acid metabolism | bna01210 | 26 | 266 | 0.0325 | 0.098 | 13 | 0.5 |
|  | Glycosphingolipid biosynthesis - globo and isoglobo series | bna00603 | 6 | 35 | 0.03564 | 0.171 | 5 | 0.833 |
|  | Glutathione metabolism | bna00480 | 33 | 360 | 0.03711 | 0.092 | 26 | 0.788 |
|  | Endocytosis | bna04144 | 50 | 593 | 0.04336 | 0.084 | 41 | 0.82 |
|  | Tryptophan metabolism | bna00380 | 20 | 200 | 0.04618 | 0.1 | 8 | 0.4 |

Table S9 Differential expression of transcription factors (TFs) in roots of canola seedlings under salt stress.

|  | Row.names | log_2_FC | padj | Gene description |
| --- | --- | --- | --- | --- |
| SS2h | BnaA01g23940D | 3.4982 | 1.30E-05 | ethylene-responsive transcription factor 1B |
|  | BnaA01g23960D | 2.1993 | 0.000103 | ethylene-responsive transcription factor ERF098 |
|  | BnaA09g05710D | 3.6036 | 0.002021 | ethylene-responsive transcription factor ERF107 |
|  | BnaAnng29660D | 3.3374 | 0.021422 | ethylene-responsive transcription factor ERF053 |
|  | BnaC07g50590D | 4.1878 | 0.014983 | ethylene-responsive transcription factor ERF054 |
|  | BnaC09g17590D | 7.1822 | 7.73E-05 | ethylene-responsive transcription factor ERF096 |
|  | BnaCnng17120D | 2.3232 | 0.017443 | ethylene-responsive transcription factor ERF070 |
|  | BnaCnng36390D | 2.4586 | 0.003772 | ethylene-responsive transcription factor ERF106-like |
|  | BnaCnng04890D | 3.6821 | 0.001169 | ethylene-responsive transcription factor ESR2-like |
|  | BnaA02g04480D | 2.8241 | 0.007218 | ethylene-responsive transcription factor RAP2-11 |
|  | BnaA10g27750D | 3.2313 | 0.000266 | ethylene-responsive transcription factor 14 |
|  | BnaC05g02080D | 2.0768 | 0.002049 | ethylene-responsive transcription factor 9 |
|  | BnaC05g02480D | 3.4594 | 1.68E-05 | ethylene-responsive transcription factor 14 |
|  | BnaA02g13710D | 3.2550 | 8.09E-10 | transcription factor bHLH109 |
|  | BnaA08g13180D | 2.5797 | 0.016194 | transcription factor bHLH146-like |
|  | BnaC02g11970D | 3.1726 | 7.61E-11 | putative transcription factor bHLH041 |
|  | BnaC03g41710D | 2.1126 | 3.83E-05 | transcription factor bHLH123 |
|  | BnaA02g24470D | 3.1193 | 3.21E-05 | probable WRKY transcription factor 8 |
|  | BnaA03g04160D | 2.0596 | 1.47E-07 | probable WRKY transcription factor 75 |
|  | BnaA06g36020D | 2.4763 | 0.011130 | probable WRKY transcription factor 8 |
|  | BnaA09g39820D | 6.6295 | 1.30E-05 | probable WRKY transcription factor 68 |
|  | BnaA09g22810D | 3.1261 | 0.000280 | transcription factor TT8-like |
|  | BnaA09g39030D | 2.1031 | 0.032377 | transcription factor MYB41 |
|  | BnaA10g30020D | 2.2095 | 0.022045 | transcription factor MYB46 |
|  | BnaAnng35730D | 4.0370 | 1.15E-08 | transcription factor MYB14 |
|  | BnaC03g17390D | 3.4360 | 9.17E-08 | transcription factor MYB14 |
|  | BnaC08g38370D | 2.8571 | 6.99E-10 | transcription factor MYB58 |
|  | BnaC03g62890D | 3.1338 | 2.18E-08 | heat stress transcription factor A-4a |
|  | BnaC09g52680D | 3.2164 | 3.81E-11 | heat stress transcription factor B-2b |
|  | BnaCnng54110D | 5.7786 | 1.12E-30 | heat stress transcription factor B-2a-like |
|  | BnaC07g49430D | 3.9307 | 0.028383 | trihelix transcription factor GTL2 |
|  | BnaA03g17210D | 2.0150 | 0.000678 | zinc finger protein ZAT11 |
|  | BnaA09g04660D | 2.1629 | 1.80E-07 | zinc finger protein 3 |
|  | BnaC04g50110D | 2.8925 | 0.001856 | zinc finger protein ZAT4 |
|  | BnaC05g34990D | 2.1136 | 4.47E-12 | zinc finger protein BRUTUS |
|  | BnaC08g08220D | 3.2561 | 1.53E-15 | zinc finger protein ZPR1 |
|  | BnaC09g41980D | 5.0544 | 0.029166 | zinc finger protein CONSTANS-LIKE 1 |
|  | BnaCnng30730D | 3.4681 | 2.68E-15 | B-box zinc finger protein 25 |
|  | BnaA06g23510D | 2.9589 | 4.94E-05 | NAC domain-containing protein 104 |
|  | BnaA06g40380D | 3.5753 | 0.00011 | NAC domain-containing protein 96 |
|  | BnaA07g10300D | 2.3338 | 4.85E-09 | NAC domain-containing protein 13 |
|  | BnaC06g13440D | 5.9991 | 0.001413 | NAC domain-containing protein 104-like |
|  | BnaC07g13550D | 2.8724 | 8.12E-11 | NAC domain-containing protein 13 |
|  | BnaCnng30500D | 2.5921 | 0.000375 | NAC domain-containing protein 104 |
| SS24h | BnaA01g13230D | -3.4493 | 0.000805 | probable WRKY transcription factor 29 |
|  | BnaA02g24470D | 3.4410 | 6.14E-05 | probable WRKY transcription factor 8 |
|  | BnaA06g33730D | 2.2456 | 5.00E-05 | probable WRKY transcription factor 3 |
|  | BnaA06g36020D | 4.2730 | 4.16E-07 | probable WRKY transcription factor 8 |
|  | BnaA09g39820D | 4.6214 | 0.037760 | probable WRKY transcription factor 68 |
|  | BnaC02g01720D | 6.9419 | 0.000118 | probable WRKY transcription factor 26 |
|  | BnaA02g13710D | 2.8423 | 2.69E-05 | transcription factor bHLH109 |
|  | BnaC07g42240D | -5.7240 | 1.19E-10 | transcription factor bHLH27 |
|  | BnaCnng19670D | -3.7874 | 1.16E-05 | transcription factor bHLH92-like |
|  | BnaCnng60760D | -6.1176 | 0.035478 | transcription factor bHLH55 |
|  | BnaA03g39790D | -3.0685 | 0.000309 | transcription factor MYB34 |
|  | BnaAnng15260D | 2.2066 | 0.003464 | transcription factor MYB41 |
|  | BnaAnng35730D | 3.6078 | 0.000277 | transcription factor MYB14 |
|  | BnaC03g17390D | 3.0363 | 0.000759 | transcription factor MYB14 |
|  | BnaC01g38060D | 2.0841 | 0.000245 | myb family transcription factor PHL6 |
|  | BnaA05g34410D | 2.3956 | 0.000208 | transcription factor IBH1 |
|  | BnaA09g39670D | 2.8475 | 7.77E-05 | transcription factor PIF6 |
|  | BnaC04g49870D | -2.7936 | 2.45E-05 | ethylene-responsive transcription factor 13 |
|  | BnaC07g31340D | 2.2753 | 0.002778 | ethylene-responsive transcription factor ERF107 |
|  | BnaC09g12830D | -4.4457 | 0.000410 | ethylene-responsive transcription factor ERF026 |
|  | BnaC09g17590D | 8.0267 | 1.24E-06 | ethylene-responsive transcription factor ERF096 |
|  | BnaCnng05370D | 2.1925 | 0.002669 | ethylene-responsive transcription factor ERF112 |
|  | BnaC03g49530D | 2.3134 | 9.50E-05 | ethylene-responsive transcription factor ABR1 |
|  | BnaCnng09130D | -2.5929 | 0.001433 | ethylene-responsive transcription factor 13 |
|  | BnaC03g62890D | 3.5171 | 4.45E-06 | heat stress transcription factor A-4a |
|  | BnaC09g52680D | 3.4781 | 4.90E-06 | heat stress transcription factor B-2b |
|  | BnaCnng54110D | 6.8815 | 4.73E-17 | heat stress transcription factor B-2a-like |
|  | BnaA03g17210D | 2.2265 | 0.019639 | zinc finger protein ZAT11 |
|  | BnaA10g16990D | 2.0124 | 0.000431 | zinc finger protein VAR3, chloroplastic-like |
|  | BnaC03g60270D | 2.9948 | 0.031722 | B-box zinc finger protein 19 |
|  | BnaC04g50110D | 3.6651 | 0.001523 | zinc finger protein ZAT4 |
|  | BnaC05g34990D | 2.7413 | 6.93E-06 | zinc finger protein BRUTUS |
|  | BnaC08g08220D | 3.6402 | 1.31E-07 | zinc finger protein ZPR1 |
|  | BnaCnng30730D | 3.5108 | 7.96E-06 | B-box zinc finger protein 25 |
|  | BnaA06g23510D | 2.3451 | 0.007795 | NAC domain-containing protein 104 |
|  | BnaA09g06870D | 2.4735 | 3.29E-07 | NAC domain-containing protein 104 |
|  | BnaC06g12550D | 3.5327 | 2.75E-05 | NAC domain-containing protein 92 |
|  | BnaCnng30500D | 4.1519 | 4.66E-06 | NAC domain-containing protein 104 |
|  | BnaCnng60710D | -2.4902 | 0.004532 | NAC domain-containing protein 90 |
| SS72h | BnaA01g07020D | -4.5726 | 0.021463 | transcription factor bHLH27 |
|  | BnaA01g17610D | -2.6412 | 0.001522 | transcription factor bHLH147 |
|  | BnaA02g13710D | 2.8634 | 5.06E-08 | transcription factor bHLH109 |
|  | BnaA08g13180D | 3.1315 | 0.000254 | transcription factor bHLH146-like |
|  | BnaA09g30620D | -4.5268 | 0.042833 | transcription factor bHLH94 |
|  | BnaA09g42370D | -5.6279 | 2.39E-14 | transcription factor bHLH19 |
|  | BnaC02g11970D | 4.2419 | 2.01E-28 | putative transcription factor bHLH041 |
|  | BnaC04g47550D | 5.4046 | 0.013994 | transcription factor bHLH100 |
|  | BnaC05g30500D | 5.1514 | 0.029696 | transcription factor bHLH117 |
|  | BnaC07g42240D | -3.8876 | 1.07E-07 | transcription factor bHLH27 |
|  | BnaCnng19670D | -4.9565 | 5.78E-12 | transcription factor bHLH92-like |
|  | BnaCnng38450D | -4.3861 | 0.036067 | transcription factor bHLH83 |
|  | BnaCnng38290D | 2.7669 | 0.043192 | bZIP transcription factor 49 |
|  | BnaA01g13230D | -4.6967 | 8.79E-07 | probable WRKY transcription factor 29 |
|  | BnaA02g24470D | 4.1011 | 4.96E-19 | probable WRKY transcription factor 8 |
|  | BnaA03g04160D | 2.6487 | 4.79E-14 | probable WRKY transcription factor 75 |
|  | BnaA06g36020D | 4.9326 | 1.84E-20 | probable WRKY transcription factor 8 |
|  | BnaA09g55250D | -2.3499 | 9.31E-12 | probable WRKY transcription factor 69 |
|  | BnaC02g01720D | 6.6880 | 8.37E-05 | probable WRKY transcription factor 26 |
|  | BnaC02g40180D | 2.0770 | 0.000693 | probable WRKY transcription factor 50 |
|  | BnaC04g01210D | -3.8445 | 2.93E-13 | probable WRKY transcription factor 46 |
|  | BnaA01g23960D | 2.5864 | 7.51E-07 | ethylene-responsive transcription factor ERF098 |
|  | BnaA07g31860D | -3.9987 | 5.08E-09 | ethylene-responsive transcription factor ERF018 |
|  | BnaA09g05710D | 4.7859 | 3.42E-10 | ethylene-responsive transcription factor ERF107 |
|  | BnaAnng13660D | -3.3304 | 1.83E-05 | ethylene-responsive transcription factor ERF115 |
|  | BnaAnng29660D | 3.5450 | 0.003145 | ethylene-responsive transcription factor ERF053 |
|  | BnaC07g13470D | -2.7062 | 8.04E-07 | ethylene-responsive transcription factor ERF019 |
|  | BnaC07g31340D | 2.4331 | 2.16E-09 | ethylene-responsive transcription factor ERF107 |
|  | BnaC07g50590D | 4.5281 | 0.002175 | ethylene-responsive transcription factor ERF054 |
|  | BnaC09g12830D | -4.5120 | 1.14E-08 | ethylene-responsive transcription factor ERF026 |
|  | BnaC09g17590D | 8.2629 | 1.04E-08 | ethylene-responsive transcription factor ERF096 |
|  | BnaCnng17120D | 3.2279 | 5.30E-08 | ethylene-responsive transcription factor ERF070 |
|  | BnaCnng36390D | 2.9147 | 0.000200 | ethylene-responsive transcription factor ERF106-like |
|  | BnaA01g23940D | 4.5666 | 8.90E-12 | ethylene-responsive transcription factor 1B |
|  | BnaA06g01090D | 2.1699 | 9.19E-10 | ethylene-responsive transcription factor 4 |
|  | BnaA09g27330D | 2.4015 | 0.000587 | ethylene-responsive transcription factor 12 |
|  | BnaA10g27750D | 3.2417 | 0.000193 | ethylene-responsive transcription factor 14 |
|  | BnaC03g58550D | -3.0447 | 0.002244 | ethylene-responsive transcription factor 12 |
|  | BnaC04g49870D | -2.7708 | 3.04E-07 | ethylene-responsive transcription factor 13 |
|  | BnaC05g02080D | 3.1208 | 2.30E-09 | ethylene-responsive transcription factor 9 |
|  | BnaC05g02480D | 2.5667 | 0.007303 | ethylene-responsive transcription factor 14 |
|  | BnaC06g41440D | 2.1888 | 3.85E-06 | ethylene-responsive transcription factor 4 |
|  | BnaCnng09130D | -3.1339 | 9.55E-08 | ethylene-responsive transcription factor 13 |
|  | BnaA02g13730D | 6.7706 | 1.34E-11 | transcription factor MYB62 |
|  | BnaA02g30960D | -2.2430 | 3.74E-12 | transcription factor MYB78 |
|  | BnaA03g39790D | -3.4521 | 3.05E-06 | transcription factor MYB34 |
|  | BnaA10g04120D | 2.1332 | 0.000195 | transcription factor MYB13 |
|  | BnaAnng35730D | 2.9150 | 0.000145 | transcription factor MYB14 |
|  | BnaC01g20650D | 2.4604 | 0.008908 | transcription factor MYB41 |
|  | BnaC02g46060D | 5.5867 | 4.33E-05 | transcription factor MYB62 |
|  | BnaC03g17390D | 2.1695 | 0.005631 | transcription factor MYB14 |
|  | BnaC08g38370D | 3.0257 | 5.07E-19 | transcription factor MYB58 |
|  | BnaC01g38060D | 2.2917 | 4.77E-08 | myb family transcription factor PHL6 |
|  | BnaA03g00390D | -2.3201 | 1.06E-06 | trihelix transcription factor GT-3a |
|  | BnaA04g29670D | -3.0855 | 1.17E-07 | transcription factor ABA-INDUCIBLE bHLH-TYPE |
|  | BnaA09g05400D | -3.8810 | 1.59E-07 | transcription factor TCP7 |
|  | BnaC02g01170D | -2.6153 | 8.71E-10 | transcription factor TCP21 |
|  | BnaC02g42220D | -2.8314 | 0.000202 | transcription factor TCP7 |
|  | BnaC09g47560D | -2.1852 | 2.57E-07 | transcription factor TCP21 |
|  | BnaA09g18400D | 2.8933 | 1.37E-21 | nuclear transcription factor Y subunit B-2-like |
|  | BnaA09g22810D | 4.5865 | 1.57E-14 | transcription factor TT8-like |
|  | BnaC03g23880D | -4.5773 | 0.029298 | transcription factor PAR1 |
|  | BnaC03g57010D | 3.9405 | 0.044778 | transcription factor SCREAM2-like |
|  | BnaC03g60240D | -2.1135 | 7.40E-11 | transcription factor VIP1-like |
|  | BnaC07g32040D | 2.2481 | 0.000251 | GATA transcription factor 6 |
|  | BnaCnng63480D | 2.3070 | 7.00E-07 | GATA transcription factor 3 |
|  | BnaC08g15800D | 2.5642 | 0.011139 | transcription factor HRS1 |
|  | BnaC08g48600D | 2.8488 | 0.002770 | transcription factor RAX3-like |
|  | BnaC03g62890D | 4.1935 | 2.11E-20 | heat stress transcription factor A-4a |
|  | BnaC09g52680D | 2.7198 | 5.07E-09 | heat stress transcription factor B-2b |
|  | BnaCnng54110D | 5.5312 | 2.54E-34 | heat stress transcription factor B-2a-like |
|  | BnaA06g23510D | 3.7839 | 9.65E-11 | NAC domain-containing protein 104 |
|  | BnaA07g10300D | 3.5261 | 5.06E-35 | NAC domain-containing protein 13 |
|  | BnaC03g50150D | -2.5720 | 3.27E-14 | NAC domain-containing protein 82-like |
|  | BnaC06g12550D | 3.3662 | 9.39E-09 | NAC domain-containing protein 92 |
|  | BnaC06g13440D | 6.2822 | 0.000458 | NAC domain-containing protein 104-like |
|  | BnaC07g13550D | 4.0485 | 5.20E-30 | NAC domain-containing protein 13 |
|  | BnaC08g49760D | 2.0476 | 0.011498 | NAC domain-containing protein 4 |
|  | BnaCnng30500D | 3.0652 | 2.48E-05 | NAC domain-containing protein 104 |
|  | BnaA03g17210D | 2.5447 | 3.51E-09 | zinc finger protein ZAT11 |
|  | BnaA07g08490D | -2.9003 | 0.015997 | dof zinc finger protein DOF1.4 |
|  | BnaA08g25740D | -2.0859 | 6.24E-14 | zinc finger protein 5 |
|  | BnaC04g50110D | 2.4874 | 0.015728 | zinc finger protein ZAT4 |
|  | BnaC05g34990D | 2.6488 | 1.08E-27 | zinc finger protein BRUTUS |
|  | BnaC08g08220D | 3.0803 | 3.38E-29 | zinc finger protein ZPR1 |
|  | BnaCnng30730D | 2.8851 | 7.68E-10 | B-box zinc finger protein 25 |
|  | BnaCnng31430D | -2.0310 | 0.044963 | dof zinc finger protein DOF3.1 |
|  | BnaCnng51540D | -2.5232 | 7.51E-05 | dof zinc finger protein DOF1.4-like |

Table S10 Primers used for qRT-PCR analysis of genes.

| Gene ID | Primer name | Sequence (5' to 3') |
| --- | --- | --- |
| 1 | *Bn ACTIN7-L* | CGCGCCTAGCAGCATGAA |
|  | *Bn ACTIN7-R* | GTTGGAAAGTGCTGAGAGATGCA |
| 2 | *BnaC05g04530D-F* | TTCTCCTCCGCCTACTTATTAC |
|  | *BnaC05g04530D-R* | CCAACTCCTCACACTCCTTAA |
| 3 | *BnaC08g44820D-F* | TTGAACTCGGCTGTGCTA |
|  | *BnaC08g44820D-R* | CGTCTGGATCTGGCTCTT |
| 4 | *BnaA09g49870D-F2* | GAGACCTCTACAGCCTCAATC |
|  | *BnaA09g49870D-R2* | ATGGCGACTGCTCTAACG |
| 5 | *BnaCnng72260D-F* | TTAGTGATGGTGTTGGCTAC |
|  | *BnaCnng72260D-R* | GGTGTTGTGAGGGAAGATAA |
| 6 | *BnaA06g37960D-F* | TCTCCTGTTGGTAACTACTTCA |
|  | *BnaA06g37960D-R* | TGTGATGCTCTTGACTCCT |
| 7 | *BnaC04g46630D-F* | CAACGCAAGCACAAGATTC |
|  | *BnaC04g46630D-R* | GTAAGCAACCCTTCCACTC |
| 8 | *BnaC04g22390D-F* | GTGGATGGGAATAAGACTCT |
|  | *BnaC04g22390D-R* | ATCAGACGAGCAACGATT |
| 9 | *BnaA06g08310D-F* | AATTCTCGGAGACGCTTC |
|  | *BnaA06g08310D-R* | GAAACCAAACGGTCCTAAAG |
| 10 | *BnaC09g51060D-F* | GATGAGCCACTACACAGAA |
|  | *BnaC09g51060D-R* | TTGCCAAGAACCGAATGA |
| 11 | *BnaA01g13540D-F* | CCTCAACAGAGAAAGCAGAAC |
|  | *BnaA01g13540D-R* | GCGTAGCCGTAAGGATTATTG |
| 12 | *BnaA10g15160D-F* | GCGTACTCATCGGTTAAGC |
|  | *BnaA10g15160D-R* | TTGGTCTCTGAAGGCATCT |

Table S11 Genes involved in plant hormone signal transduction in canola roots under salt stress.

|  |  | Row.names | log_2_FoldChange | pvalue | Gene description |
| --- | --- | --- | --- | --- | --- |
| 2 h | Plant hormone signal transduction | BnaA01g23940D | 3.50 | 0.000001 | ethylene-responsive transcription factor 1B |
|  |  | BnaA07g38130D | 4.62 | 3.07E-17 | abscisic acid receptor PYL9 |
|  |  | BnaAnng25820D | 2.06 | 0.009107 | ethylene response sensor 2 |
|  |  | BnaC01g18020D | 2.90 | 1.82E-11 | protein phosphatase 2C 56-like |
|  |  | BnaC01g19430D | 4.22 | 6.85E-28 | indole-3-acetic acid-amido synthetase GH3.5 |
|  |  | BnaC04g22040D | 2.40 | 5.4E-11 | auxin-responsive protein SAUR36 |
|  |  | BnaC05g00620D | 2.16 | 0.000185 | abscisic acid receptor PYL9 |
|  |  | BnaC05g11370D | 5.92 | 0.000224 | auxin-responsive protein IAA34 |
|  |  | BnaCnng68710D | 3.56 | 0.000001 | abscisic acid receptor PYL10 |
|  | MAPK signaling pathway - plant | BnaA01g23940D | 3.50 | 0.000001 | ethylene-responsive transcription factor 1B |
|  |  | BnaA07g38130D | 4.62 | 3.07E-17 | abscisic acid receptor PYL9 |
|  |  | BnaAnng25820D | 2.06 | 0.009107 | ethylene response sensor 2 |
|  |  | BnaC01g18020D | 2.90 | 1.82E-11 | protein phosphatase 2C 56-like |
|  |  | BnaC05g00620D | 2.16 | 0.000185 | abscisic acid receptor PYL9 |
|  |  | BnaCnng68710D | 3.56 | 0.000001 | abscisic acid receptor PYL10 |
|  | Phosphatidylinositol signaling system | BnaA09g35480D | 2.09 | 0.000002 | phosphoinositide phospholipase C 7 |
| 24 h | Plant hormone signal transduction | BnaA07g38130D | 5.82 | 1.69E-12 | abscisic acid receptor PYL9 |
|  |  | BnaA08g01770D | 2.11 | 0.000102 | auxin-responsive protein IAA18-like |
|  |  | BnaAnng25820D | 3.21 | 3.58E-06 | ethylene response sensor 2 |
|  |  | BnaC01g18020D | 3.03 | 2.99E-13 | protein phosphatase 2C 56-like |
|  |  | BnaC01g19430D | 4.91 | 3.00E-19 | indole-3-acetic acid-amido synthetase GH3.5 |
|  | MAPK signaling pathway - plant | BnaA07g38130D | 5.82 | 1.69E-12 | abscisic acid receptor PYL9 |
|  |  | BnaAnng25820D | 3.21 | 3.58E-06 | ethylene response sensor 2 |
|  |  | BnaC01g18020D | 3.03 | 2.99E-13 | protein phosphatase 2C 56-like |
|  |  | BnaC07g15270D | 2.11 | 6.00E-07 | catalase-1 |
|  |  | BnaC08g49010D | 2.20 | 2.21E-05 | 1-aminocyclopropane-1-carboxylate synthase-like protein 1 |
| 72 h | Plant hormone signal transduction | BnaA01g23940D | 4.57 | 2.23E-13 | ethylene-responsive transcription factor 1B |
|  |  | BnaA06g09800D | 2.33 | 0.002615 | auxin-responsive protein IAA34 |
|  |  | BnaA07g38130D | 4.33 | 6.83E-16 | abscisic acid receptor PYL9 |
|  |  | BnaAnng25820D | 3.24 | 2.59E-06 | ethylene response sensor 2 |
|  |  | BnaC01g18020D | 4.04 | 7.08E-35 | protein phosphatase 2C 56-like |
|  |  | BnaC01g19430D | 2.56 | 8.41E-10 | indole-3-acetic acid-amido synthetase GH3.5 |
|  |  | BnaC03g45470D | 3.34 | 1.53E-05 | pathogenesis-related protein 1 |
|  |  | BnaC04g22040D | 2.40 | 9.12E-12 | auxin-responsive protein SAUR36 |
|  |  | BnaC05g00620D | 2.82 | 7.64E-09 | abscisic acid receptor PYL9 |
|  |  | BnaC05g11370D | 5.14 | 0.007906 | auxin-responsive protein IAA34 |
|  |  | BnaCnng68710D | 3.86 | 1.55E-07 | abscisic acid receptor PYL10 |
|  | MAPK signaling pathway - plant | BnaA01g23940D | 4.57 | 2.23E-13 | ethylene-responsive transcription factor 1B |
|  |  | BnaA07g38130D | 4.33 | 6.83E-16 | abscisic acid receptor PYL9 |
|  |  | BnaAnng25820D | 3.24 | 2.59E-06 | ethylene response sensor 2 |
|  |  | BnaC01g18020D | 4.04 | 7.08E-35 | protein phosphatase 2C 56-like |
|  |  | BnaC03g45470D | 3.34 | 1.53E-05 | pathogenesis-related protein 1 |
|  |  | BnaC05g00620D | 2.82 | 7.64E-09 | abscisic acid receptor PYL9 |
|  |  | BnaC08g49010D | 2.92 | 1.16E-22 | 1-aminocyclopropane-1-carboxylate synthase-like protein 1 |
|  |  | BnaCnng68710D | 3.86 | 1.55E-07 | abscisic acid receptor PYL10 |
